# Exploring the Landscape of Heterocyclic Quinones for Redox Flow Batteries

**DOI:** 10.1021/acsaem.3c02223

**Published:** 2023-12-28

**Authors:** Rajesh B. Jethwa, Dominic Hey, Rachel N. Kerber, Andrew D. Bond, Dominic S. Wright, Clare P. Grey

**Affiliations:** Yusuf Hamied Department of Chemistry, University of Cambridge, Lensfield Road, Cambridge CB2 1EW, U.K.

**Keywords:** quinones, heterocycles, redox flow, battery, in situ, anolyte, aqueous

## Abstract

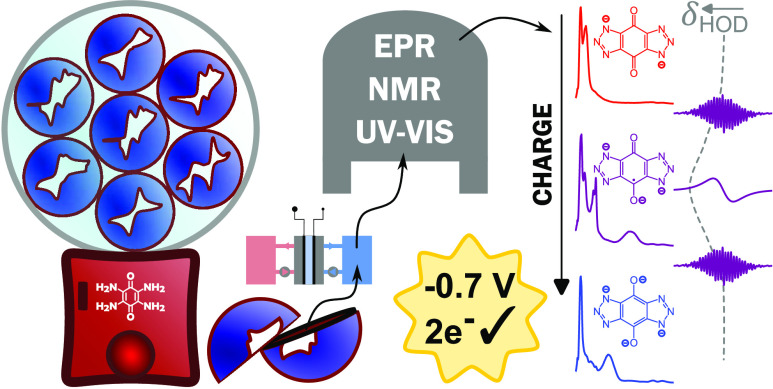

Redox flow batteries (RFBs) rely on the development of cheap, highly soluble, and high-energy-density electrolytes. Several candidate quinones have already been investigated in the literature as two-electron anolytes or catholytes, benefiting from fast kinetics, high tunability, and low cost. Here, an investigation of nitrogen-rich fused heteroaromatic quinones was carried out to explore avenues for electrolyte development. These quinones were synthesized and screened by using electrochemical techniques. The most promising candidate, 4,8-dioxo-4,8-dihydrobenzo[1,2-*d*:4,5-*d*′]bis([1,2,3]triazole)-1,5-diide (−0.68 V(SHE)), was tested in both an asymmetric and symmetric full-cell setup resulting in capacity fade rates of 0.35% per cycle and 0.0124% per cycle, respectively. In situ ultraviolet-visible spectroscopy (UV–Vis), nuclear magnetic resonance (NMR), and electron paramagnetic resonance (EPR) spectroscopies were used to investigate the electrochemical stability of the charged species during operation. UV–Vis spectroscopy, supported by density functional theory (DFT) modeling, reaffirmed that the two-step charging mechanism observed during battery operation consisted of two, single-electron transfers. The radical concentration during battery operation and the degree of delocalization of the unpaired electron were quantified with NMR and EPR spectroscopy.

## Introduction

**Figure 1:**
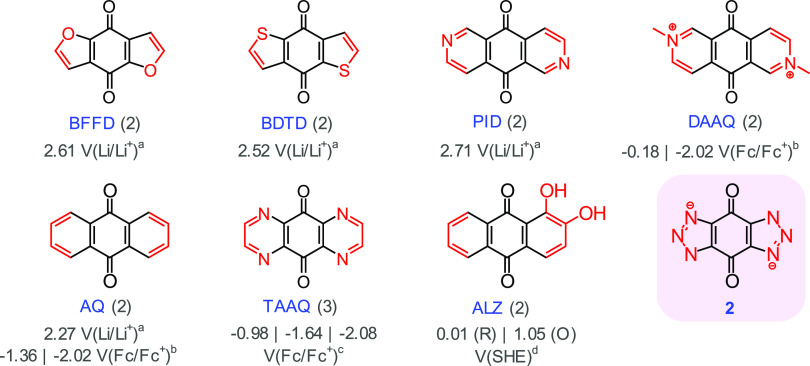
Examples of heteroaromatic quinone-derived systems are reported in the literature. The number of electrons involved in redox reactions are included in parentheses. BFFD = benzo[1,2-*b*:4,5-*b*′]difuran-4,8-dione, BDTD = benzo[1,2-*b*:4,5-*b*′]dithiophene-4,8-dione, PID = pyrido[3,4-*g*]isoquinoline-5,10-dione,^23^ DAAQ = 2,7-dimethyl-5,10-dioxo-5,10-dihydropyrido[3,4-*g*]isoquinoline-2,7-diium,^25^ AQ = anthracene-9,10-dione,^23,24^ TAAQ = pyrazino[2,3-*g*]quinoxaline-5,10-dione,^26^ ALZ = 1,2-dihydroxyanthracene-9,10-dione,^27^**2** = 4,8-dioxo-4,8-dihydrobenzo[1,2-*d*:4,5-*d*′]bis([1,2,3]triazole)-1,5-diide.^28,29^ The purple box indicates the molecule studied in this paper. ^a^Reported first-stage discharge voltages vs Li/Li^+^ in 1 M LiPF_6_/EC:DMC (1:1).^23^^b^Reported redox potentials vs Fc/Fc^+^ in 0.1 M TBAPF_6_/MeCN.^25^^c^Reported redox potentials vs Fc/Fc^+^ in 0.1 M TBABF_4_/dimethyl sulfoxide (DMSO).^26^^d^Reported anode and cathode potentials vs standard hydrogen electrode (SHE).^27^ (R) denotes reduction, while (O) denotes oxidation.

Within the domain of carbonyl-based electrolytes, much of the organic redox flow battery (RFB) literature explores higher-order quinones due to their greater chemical stability.^1^ Anthraquinones (AQs) offer a greater degree of charge delocalization and higher chemical stability compared to benzoquinone (BQ) alternatives,^1^ and so it not surprising that this class of molecules has been the most extensively explored in both nonaqueous and aqueous electrolytes.^1^ Perhaps the most well-known examples are those reported by the Aziz group who explored the effects of different pendant functional groups on the solubility and stability of the AQ-based electrolytes for long-term battery performance.^2−7^ Modification of anthraquinone and naphthoquinone frameworks with solubilizing groups has been explored extensively,^1,8−13^ but other electrolytes that operate on the same principle of fused-ring systems for greater chemical and electrochemical stability have also been developed, both within RFBs and within the broader battery literature. For example, annulated rings have been used to prevent degradation by electrophilic or nucleophilic addition to the quinone during cycling.^14^ Heteroaromatic molecules are often explored due to their redox properties, e.g., tetrathiofulvalene-,^15,16^ phenazine-,^17−19^ or quinoxaline-based electrolytes,^20−22^ or for the ability to modify the charge mobility, energy gaps, and intermolecular interactions.^23^ One such example can be seen in the study of fused heteroaromatics for organic electrodes in lithium-ion batteries (LIBs),^23,24^ where the electrochemical properties of a series of fused heteroaromatics, including bis-furan (BFFD, Figure 1), bis-thiophene (BDTD, Figure 1), and bis-pyridine (PID, Figure 1) quinone derivatives were studied and compared to AQ (Figure 1).^23^

Fused heteroaromatics provide a means of introducing positively or negatively charged sites into a molecule, which is a strategy often used to increase the solubility in aqueous media. For example, dimethylation of pyrido[3,4-*g*]isoquinoline-5,10-dione (PID), to form 2,7-dimethyl-5,10-dioxo-5,10-dihydropyrido[3,4-*g*]isoquinoline-2,7-diium (DAAQ, 0.25 V(SHE),^25^Figure 1) was explored in the area of hydrogen peroxide production and was shown to greatly improve solubility compared to AQ, the redox reactions being shifted to more positive values by the greater electron-withdrawing effect of the pyridinium sites.^25^ Heteroaromatics can also provide a greater ability to store electrons by the introduction of additional redox processes. One such example is pyrazino[2,3-*g*]quinoxaline-5,10-dione (TAAQ, −0.58 V(SHE), Figure 1) which undergoes three single-electron reductions: two to reduce the quinone to its hydroquinone analogue, and a further reduction to form a nitrogen radical within the flanking heteroaromatic rings.^26^ For this reason, fused heteroaromatic electrolytes have been proposed for symmetric RFBs.^30^ Moreover, a symmetric conventional battery with an aqueous electrolyte has already been demonstrated using Alizarin (ALZ, 1,2-dihydroxyanthracene-9,10-dione, Figure 1) with a cell voltage of 1.04 V, where the two quinone rings account for the redox processes at each electrode.^27^ However, while fused heteroaromatics have seen greater exploration in conventional batteries, these compounds have not been as widely explored within the flow battery literature.

**Scheme 1:**
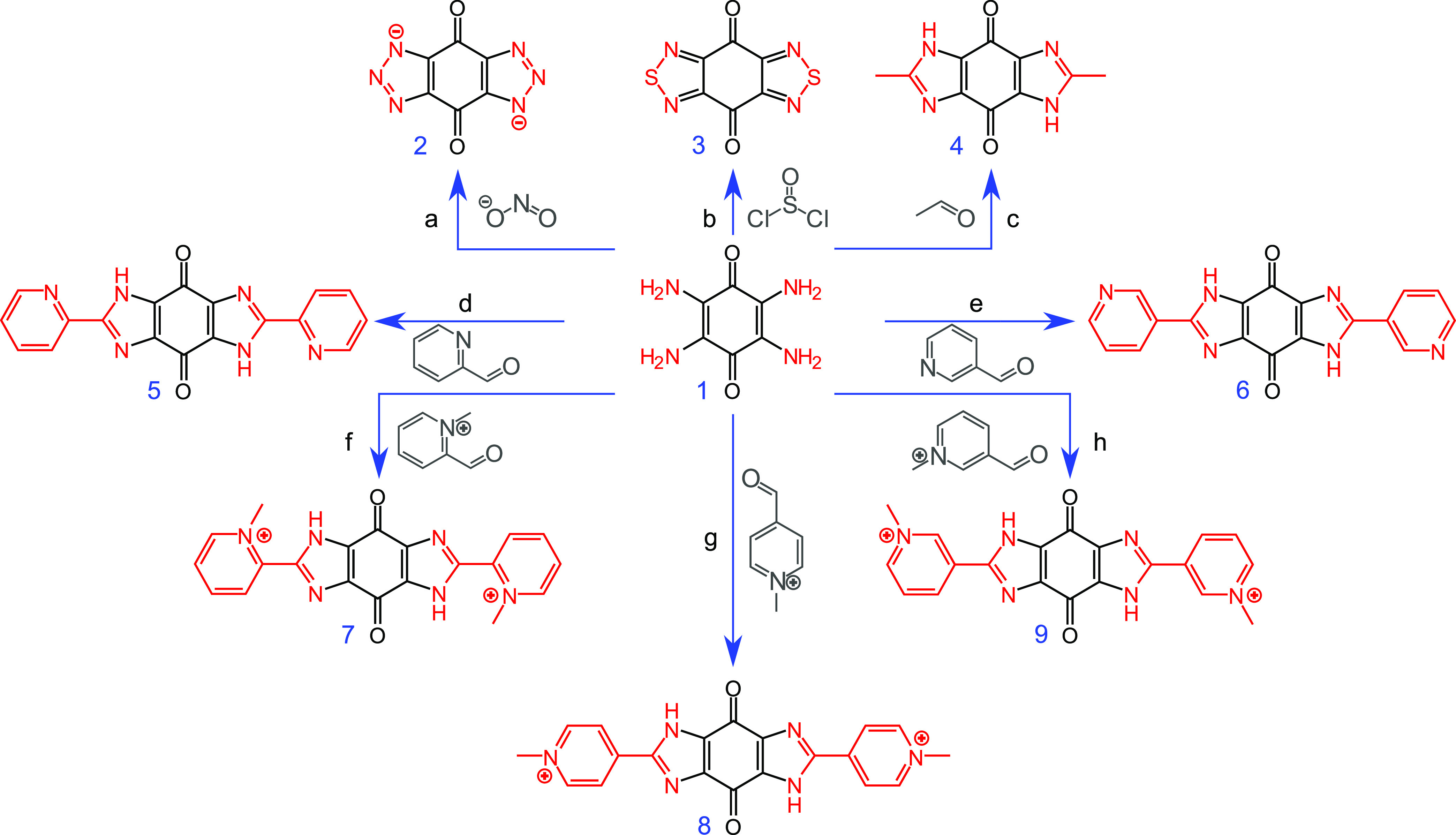
General Synthetic Routes from Tetra-Amino-Benzoquinone (**1**) to Obtain the Different Species Explored in This Work (a) Sodium nitrite, acetic acid to form **2**, (b) thionyl chloride, Δ to form **3**, (c) acetaldehyde, Δ to form **4**, (d) 2-pyridine carboxaldehyde, tosic acid, Δ to form **5**, (e) 3-pyridine carboxaldehyde, Δ to form **6**, (f) 2-pyridinium carboxaldehyde iodide, Δ to form **7**, (g) 4-pyridinium carboxaldehyde iodide, Δ to form **8**, (h) 3-pyridinium carboxaldehyde, Δ to form **9**. The solubility data for these compounds is presented in Table S2.

Herein, the intention was to expand the scope of such compounds through the principle of umpolung (polarity inversion). Therefore, tetra-amino-BQ (**1**, Scheme 1)^31−33^ was chosen as the starting point. The electrochemical behavior of **1** has previously been reported in nonaqueous media (*N*,*N*′-dimethylformamide, DMF),^28^ where it was shown to be unsuitable for aqueous applications. However, compound **2** (Figure 1, Scheme 1), with a motif more in-line with the aforementioned literature examples (Figure 1), was identified as a more promising species for use in water-based systems.^29^

The reported electrochemistry of **2** in a nonaqueous environment (DMF) showed that the degree of protonation influenced both the degree of separation between the redox processes, and the redox potential.^28^ Additionally, in a basic aqueous environment, the half-wave potentials of **2** in the presence of different cations (Li^+^, Na^+^, K^+^, and tetrabutylammonium (TBA^+^)) were reported to depend on the ionic activity of the cation.^29^ This ability to influence the redox potential by the choice of the cation could theoretically be a simple means to further fine-tune the voltage of a RFB. In the case of **2**, larger, less coordinating cations lower the redox potentials, while smaller, more coordinating cations raise the redox potential.^29^ The solubility in various alkaline media was reported as >0.442 M in 1 M LiOH, 0.035 M in 1 M KOH, and 0.4 mM in 1 M NaOH.^29^ With the potential for two-electron storage and a solubility surpassing 0.4 M, **2** has a theoretical capacity of >23.6 A h·l^–1^ (in LiOH) but so far its full-cell performance and long-term electrochemical stability have not been investigated.^8^

Alternative species to **2** were also considered in this work to extend the scope of heteroaromatic compounds considered for RFBs. Benzothiadiazole has been used as an anolyte in nonaqueous RFBs,^34^ and with the inclusion of an electron-donating methoxy group, the reduction potential was shown to be cathodically shifted.^35^ Bis-imidazolyl-quinones (like **4**, Scheme 1) have been investigated as cyanide sensors in which the cyanide anion hydrogen bonds to the imidazolic proton, decreasing the redox potential by increasing the electron density on the heterocyclic nitrogen.^31^ However, to our knowledge, neither the bis-thiadiazolyl-quinone nor bis-imidazolyl-quinones (**3**, **4**, Scheme 1) have yet been considered for redox flow batteries. Additionally, like **2**, the imidazole framework (**4**) is expected to be fully deprotonated at a high pH but comes with the ability to introduce additional organic units into the electrolyte framework by virtue of the higher valency of carbon, i.e., replacing the methyl group in **4** with other moieties. These additional units may provide added benefits, either in the form of increased solubility or through the introduction of additional redox sites, e.g., metal complexation (to **5** and **6**), or pyridinium fragments (**7**, **8**, and **9**) for viologen-like redox and/or to promote solubility at neutral pH. The additional redox sites could enhance the efficacy of the electrolyte or facilitate a symmetric RFB arrangement. The former would increase the number of electrons that are stored or given up, thereby increasing the energy density of the electrolyte as an anolyte or catholyte, respectively. However, the latter would be achieved by introducing a redox reaction that would function in the opposite manner to that of the quinone itself, i.e., an oxidizable moiety on an anolyte scaffold or vice-versa.

In this work, asymmetric and symmetric galvanostatic cycling between predetermined potential limits (galvanostatic cycling with potential limitation, GCPL) with in situ spectroscopic analysis (UV–Vis, NMR, and EPR) are carried out to gain insight into the states of charge of the molecule as well as the stability of the system over time. Density functional theory (DFT) was used to support the results obtained by different spectroscopies used in this study. Cyclic voltammetry (CV) was also used to identify the stability of **2** with respect to the supporting electrolyte over time by collecting cycles continuously over an extended period. **2** demonstrated a low reduction potential of −0.68 V(SHE), a high stability against hydroxide ions, and a slow capacity fade rate in the symmetric (0.0124% per cycle) flow battery experiment. Alternative electrolyte motifs using the scaffold of **1** were also tested in addition to **2**. **3** and **4** were chosen as target candidates, replacing the central nitrogen of **2** with a sulfur atom or a carbon atom, respectively. Compounds **5**–**9** (Scheme 1) were also synthesized and tested for their electrochemical performance to screen a series of heteroaromatic quinones to gain insight into which motifs should be further explored.

## Electrochemical Screening

**Figure 2:**
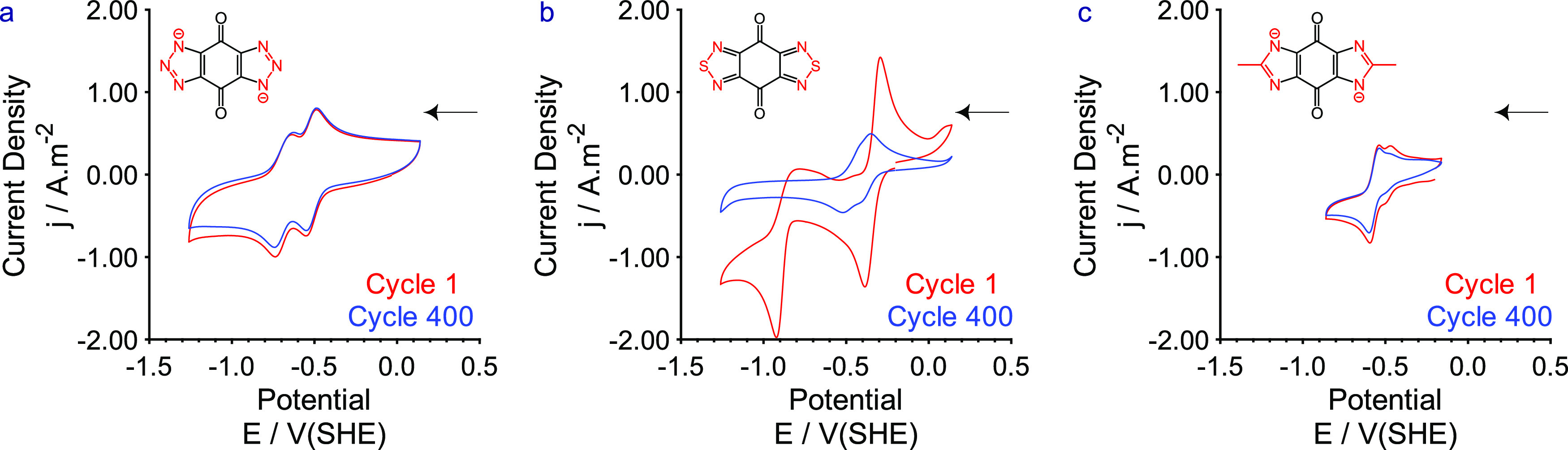
Cyclic voltammograms from cycles 1 (red) and 400 (blue) of 1 mM solutions of **2** (a), **3** (b), and **4** (c) in basic aqueous (D_2_O) media ((a) 1 M LiOH, (b, c) 1 M KOH). Note: the results with LiOH are presented here for (a) rather than KOH. The scan rate was 20 mV s^–1^, scanning toward negative potentials first (as indicated by the black arrow). A glassy-carbon electrode (3 mm diameter) was used alongside a coiled platinum-wire counter electrode and a mercury–mercury oxide reference electrode. The experiments were carried out at 25 °C.

Figure 2 shows CV cycles 1 and 400 of the three species **2**–**4** in basic media. As can be seen from the figure, the replacement of the central nitrogen has a significant impact on the electrochemical activity and stability of the compounds investigated. As qualitatively similar electrochemistry for **2** in the presence of either LiOH or KOH was reported in ref (29), CV of **2** (Figure 2a) was carried out using 1 M solutions of LiOH, as this was the medium in which the highest solubility (>0.442 M cf. 0.035 M in 1 M KOH) had been stated by Bunzen et al.^29^ The performance matched the previously reported behavior, with two slightly overlapping redox processes and clearly defined peaks for both oxidations and reductions. *E*_1/2_ potentials of −0.52 and −0.68 V(SHE) were observed with the lower potential redox couple being quasi-reversible in nature (see Figures S3 and S4). No significant changes were observed in the electrochemical profile or currents recorded in the experiment over 2000 cycles (see Figure S3) indicating reasonable stability of the molecule with regard to hydroxide as the supporting electrolyte.

**3** was clearly an unsuitable anolyte in basic aqueous media: while initially presenting a pair of redox couples at −0.34 and −0.86 V(SHE) (Figure 2b), rapid degradation was observed to occur in the presence of hydroxide. Over the course of the subsequent 56 cycles, the intensity of the current of the redox couple at −0.86 V(SHE) rapidly diminished, and the redox couple at −0.34 V(SHE) split into an overlapping set of redox processes centered around −0.43 V(SHE) with poorly defined oxidation and reduction peaks. Both processes decreased in current intensity over time as the solution changed color (see Figure S10) and eventually became colorless. As the control sample, upon which no electrochemistry had been carried out, also became colorless over time, the degradation of **3** in basic media appears to be chemical rather than electrochemical. Nucleophilic attack at either the imine-like carbon or at the sulfur by hydroxide may have ensued, resulting in ring-opening or desulfurisation.^36^ Nonaqueous conditions may be more advantageous for this compound but were not investigated here.

Cyclic voltammetry of **4** (Figure 2c) demonstrated that it was more stable than **3**, but it was a worse candidate electrolyte than **2**. In addition to a lower peak current density, **4** also initially presents two redox processes, with the loss of the minor redox process over the course of the CV experiment with a concomitant broadening (see Figure S11) in the peak-to-peak separation of the major redox process at −0.57 V(SHE). This process is higher in potential than the redox processes observed in **2** and would therefore result in a lower cell voltage overall.

**Figure 3:**
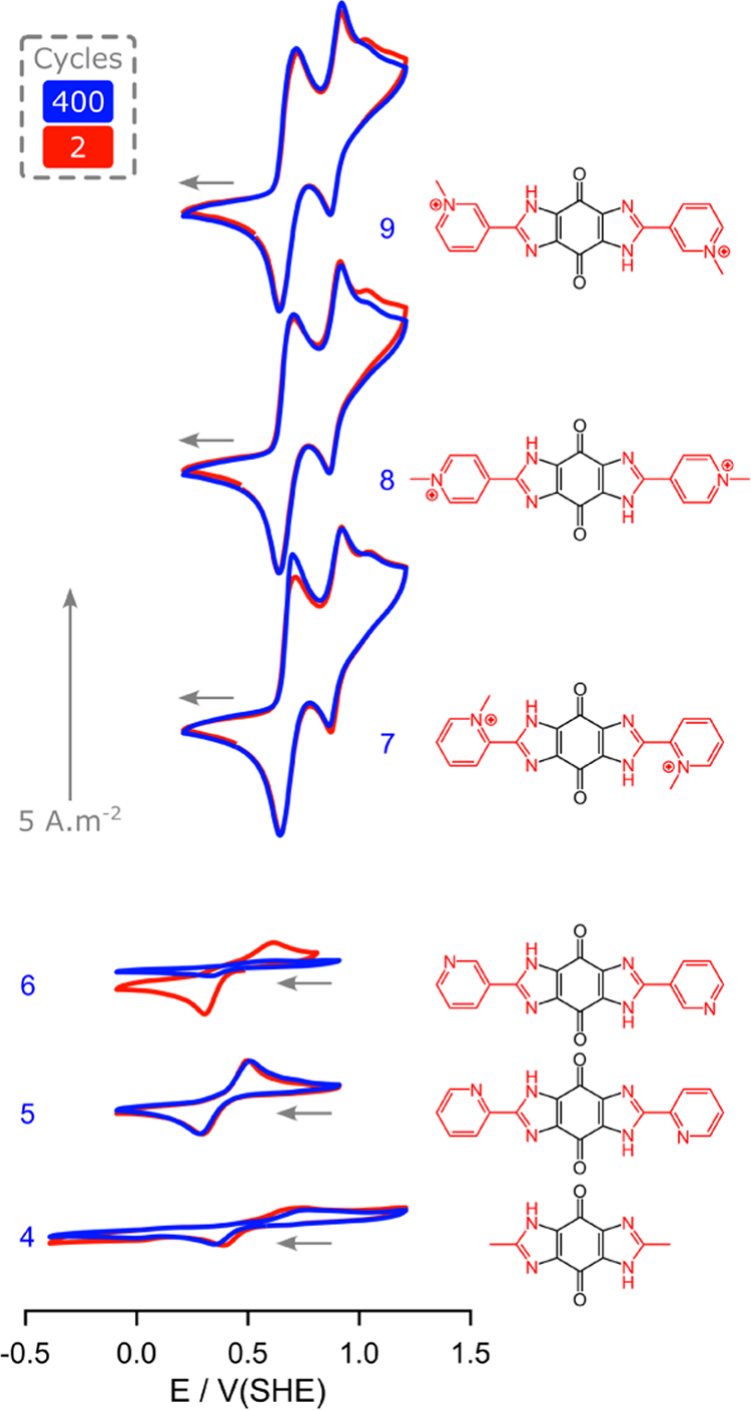
Cyclic voltammograms from cycles 2 and 400 of (bottom-to-top) 1 mM solutions of **4**–**9** in acidic (1 M HCl) aqueous (D_2_O) media. The scan rate was 20 mV s^–1^, scanning toward negative potentials first (as indicated by the gray arrow). A glassy-carbon electrode (3 mm diameter) was used alongside a coiled platinum-wire counter electrode and a silver–silver chloride reference electrode. The experiments were carried out at 25 °C.

Regrettably, while compounds **5**–**9** were synthesized, these compounds were found to be insoluble under alkaline conditions. For this reason, their electrochemical performance was investigated under acidic conditions (Figure 3).

From the voltammetry in Figure 3, compounds **7**–**9** appear promising in acidic media, though acidity reintroduces system-level issues of corrosion and a requirement for an acid-stable catholyte. However, the greater concern for these systems is their overall poor solubility (<20 mM, see Table S2). Further optimization of these frameworks is required to enhance their feasibility for flow battery systems, ideally to facilitate neutral or basic pH battery systems.

## Asymmetric Cells

Based on the initial CV and solubility screening, the study of **2** was continued in asymmetric and symmetric full-cell RFB arrangements.

The battery performance of **2** was assessed in a 5 cm^2^ lab-scale cell by using Nafion 212 as the cation-exchange membrane and baked carbon paper as the electrode material. As the solubility of protonated **2** was reported to be greatest in LiOH,^29^ this was chosen as the supporting electrolyte with D_2_O being used to facilitate both in situ and postmortem ex situ NMR analysis of the solutions.

**Figure 4:**
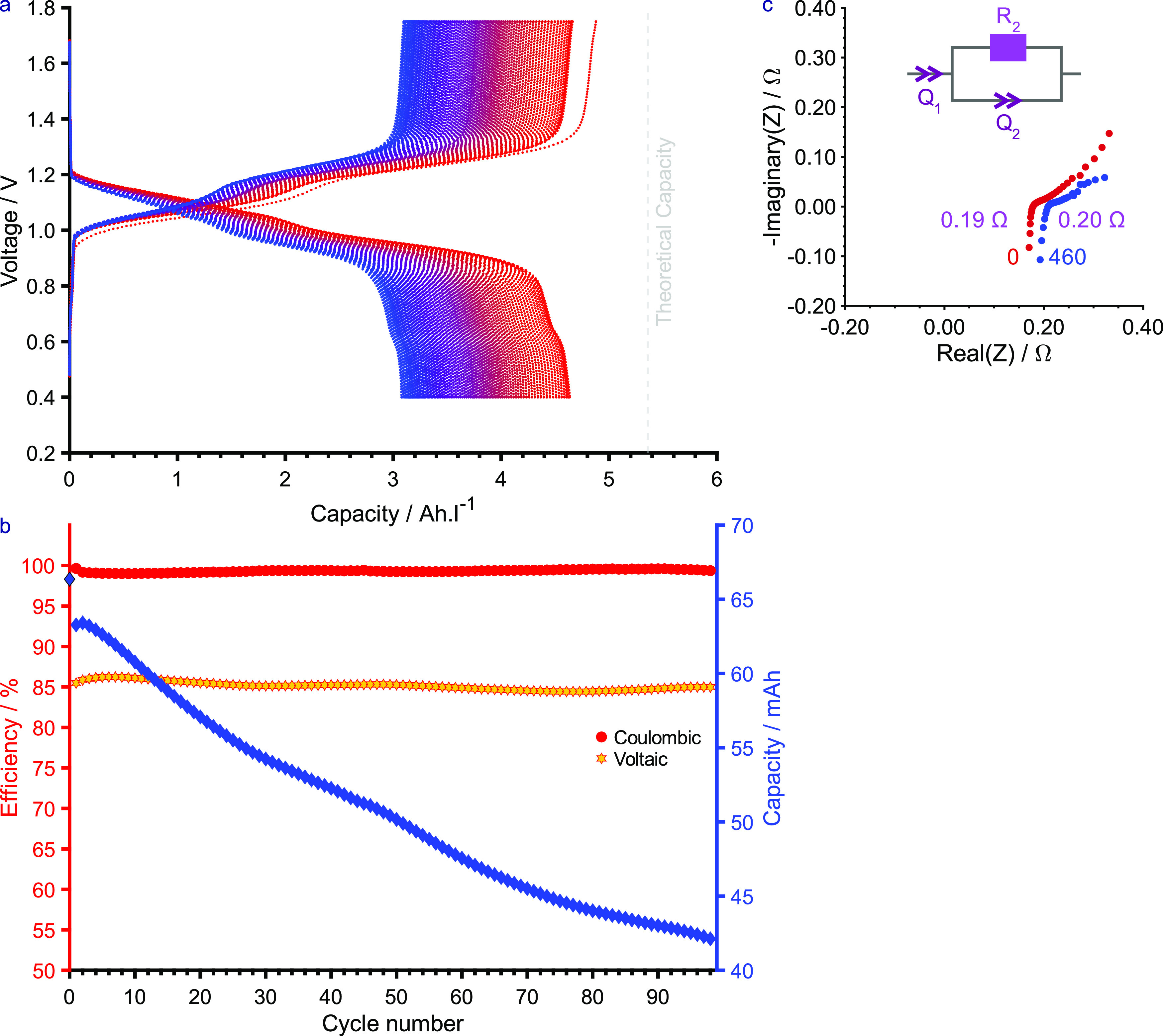
Lab-scale RFB performance of **2** against potassium ferrocyanide in D_2_O with 1 M LiOH added as the supporting electrolyte at ambient temperature under an inert nitrogen atmosphere. The battery was operated between 0.40 and 1.75 V at a current of 200 mA (40 mA·cm^–2^). (a) Voltage vs capacity, (b) Coulombic efficiency and Voltaic efficiency vs cycle number and capacity vs cycle number, (c) electrochemical impedance spectroscopy taken before (0, red) and after 460 cycles (blue) of battery cycling. The equivalent circuit shown was used to fit the data and the resistances, corresponding to *R*_2_, are denoted in purple. Note: The overall energy efficiency, a product of both Coulombic and Voltaic efficiencies, closely mirrors the trajectory of Voltaic efficiency since the Coulombic efficiency is greater than 99%.

Figure 4a,b shows the full-cell RFB performance over the first 100 cycles (Figure S26 for the extended cycling data). In the first cycle, 91% of the theoretical capacity was achieved, dropping to 87% in the second cycle. The capacity fade was 0.22 mA h per cycle (*R*^2^ = 0.9727, 0.35% per cycle) with a Coulombic efficiency (CE) of 99.332 ± 0.017% and a Voltaic efficiency (VE) of 85.14 ± 0.05%. The overall energy efficiency (EE) was therefore 84.57 ± 0.06%, as a product of both CE and VE, with its evolution closely mirroring that of VE, as CE remained >99% during cycling.

The rapid capacity fade observed was attributed to crossover, as evidenced by membrane fouling (Figure S29) and an increase in impedance (Figure 4c). Crossover causes capacity loss as the anolyte or catholyte is lost to the opposing tank.^37^ While symmetric systems can be rebalanced as the species on each side are the same, for an asymmetric system this is not possible and cross-contamination may lead to irreversible degradation of the catholyte or anolyte in the opposing environment.^37,38^ There was no significant change in the volumes of the two tanks after cycling, suggesting that there was no significant bias for solvent to move from one tank to the other, but significant discoloration of the initially colorless membrane was observed upon postmortem analysis (Figure S29) indicating uptake and, most likely, passage of **2**.

Membrane selectivity is typically determined by three factors: physical, electrostatic, and Donnan exclusion.^1,38,39^ Diffusion, migration, and electroosmotic drag have previously been identified to be the most dominant mechanisms for crossover in an anion-exchange membrane,^37^ and recent work on cation-exchange membranes has highlighted that while increasing the size of the molecule and charge can reduce crossover, it is the latter that has the greatest influence.^38^ It might therefore be surprising that either **2** or its reduced form—both of which contain multiple negative charges—were able to discolor the pretreated and similarly negatively charged, poly(perfluorosulfonic acid) cation-exchange membrane.^40,41^ However, other examples of similarly negatively charged compounds crossing over during battery operation have been presented previously,^42−46^ and it has been suggested that once the channels become saturated with the redox species of interest then crossover is possible.^46^ Alternatively, the membrane pretreatment protocol (as described in ref (2), see the Supporting Information (SI)) may also have been partly responsible. As-bought Nafion is acidic, but for high pH systems, it typically requires ion exchange and consumption of the protons so as not to result in unwanted pH changes. It has been suggested that to activate Nafion for alkaline media, it should be heated with peroxide and then soaked in a dilute solution of the supporting electrolyte,^2,40,46^ however, recent reports have also suggested that an overnight soak may be sufficient.^38,47^ In this regard, it is possible that the use of peroxide may have resulted in damage to the membrane’s channels and/or pore-structure enabling a greater rate of crossover than would otherwise have been observed.

Nonetheless, the loss of active material by crossover is likely to explain the fade rate observed in Figure 4a,b. This is further supported by postmortem NMR analysis of the electrolyte tanks (see Figure S30). The ^1^H NMR of both the anolyte and catholyte shows several signals where no protons are expected for either compound. The signals may arise from synthetic impurities or the degradation of **2**. The ^13^C NMR spectrum for the anolyte tank exhibited one major peak and several small peaks, likely from synthetic impurities (see Figure S48), where only two peaks were expected in total. Similarly, the catholyte tank exhibited two peaks where only one was expected. While this could be due to the lower sensitivity of ^13^C NMR, at the very least, the presence of similar peaks in both tanks suggests that crossover took place. Cyclic voltammetry of a mixed-electrolyte system over time (Figure S8) demonstrated loss of electrochemical activity suggesting that crossover, followed by deactivation or degradation of either **2** or hexacyanoferrate may explain the observed capacity fade in Figure 4.

## Ultraviolet–Visible Spectroscopy

The battery scale performance of **2** discussed above suffers from issues regarding irreversible electrochemical plateaus at high voltage (∼1.7 V, see the Supporting Information), loss of material to the membrane and crossover, and a lower than ideal Coulombic efficiency. To identify if **2** itself was responsible for these properties, further investigations were carried out using in situ UV–Vis, NMR, and EPR spectroscopy during battery operation.

**Figure 5:**
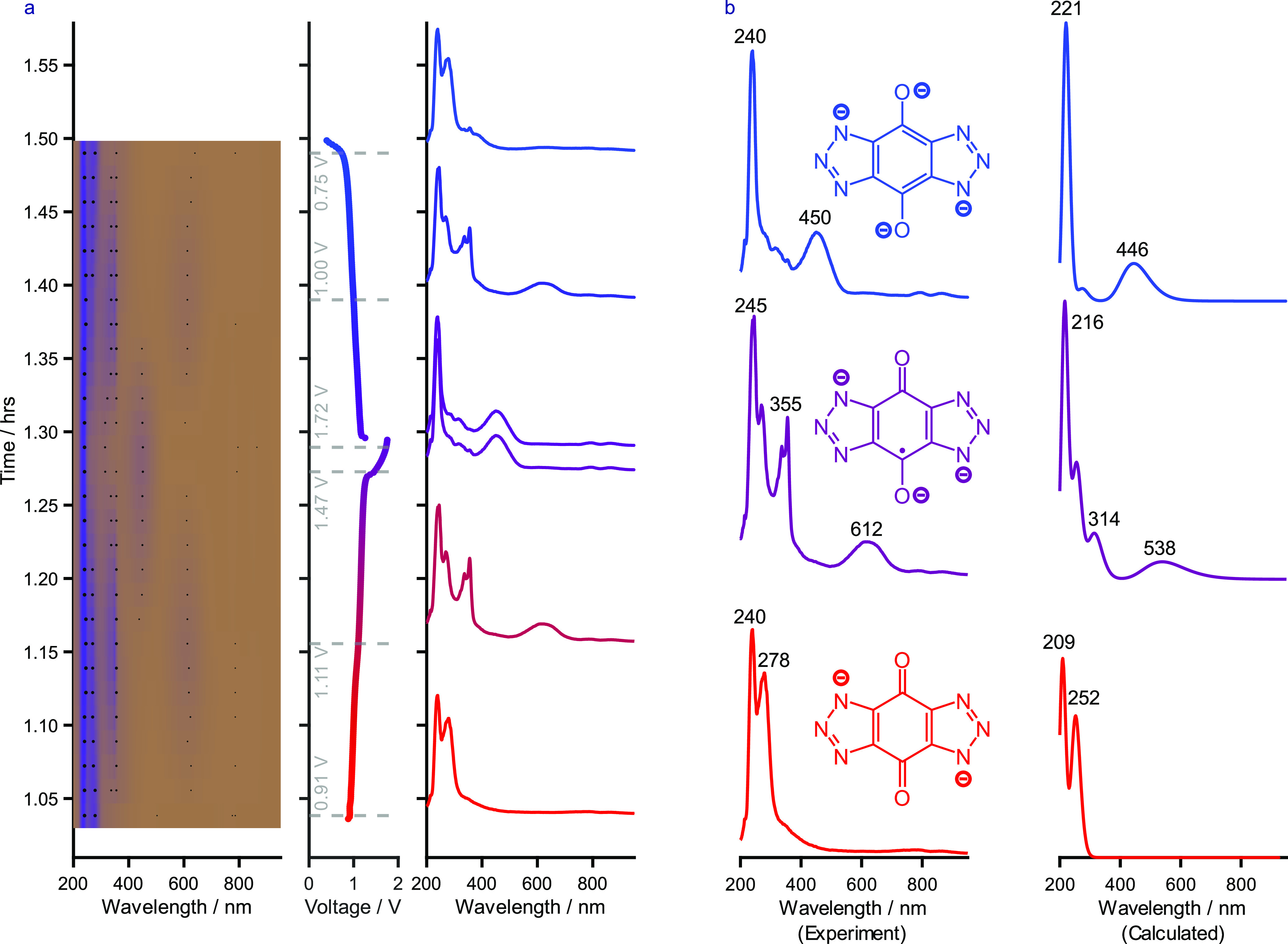
(a) In situ UV–Vis from a lab-scale RFB of **2** against potassium ferrocyanide in D_2_O with 1 M LiOH added as the supporting electrolyte at ambient temperature under an inert nitrogen atmosphere. The battery was operated between 0.40 and 1.75 V at a current of 20 mA (4 mA·cm^–2^). The left graph displays the data as a heatmap over time (black dots are added as a guide to the eye), the middle graph displays the electrochemical data over time, and the right graph displays the UV–Vis spectra extracted at the time points denoted by the gray dashed lines shown on the electrochemical profile. The relevant voltages have also been added adjacent to the gray markers. (b) Experimental (left) and calculated (right) ultraviolet–visible spectra of **2** in its oxidized (red), singly reduced (purple), and doubly reduced states (blue). The calculated spectra were produced at the CAM-B3LYP/TZVP level of theory (TD-DFT, 200 states, 50:50 (triplet/singlet)).

Figure 5a shows how the UV–Vis behavior of **2** changed during the charge and discharge (Table 1). As **2** was charged from its quinoidal state (oxidized) to its semiquinoidal state (singly reduced) during the first plateau, the color of the solution changed from a deep-green color to a deep blue. The major change observed is the growth of a broad peak at 612 nm as well as a sharp peak at 355 nm. As the molecule is reduced further during the second plateau to its hydroquinoidal state (doubly reduced), the solution became yellow. The peak at 612 nm was lost, and a broad peak at 451 nm was observed instead. Additionally, the peak structure between 250 and 350 nm was lost, and a new peak at 315 nm was observed.

**Table 1:** Peaks Observed in the Ultraviolet–Visible Spectrum of **2** during Battery Operation

molecular state	experimental peaks/nm (normalized intensity/%)	calculated peaks/nm (normalized intensity/%)
quinone	240.12 (100), 278.37 (81)	209 (72), 252 (51)
semiquinone	245.11 (100), 269.5 (66), 355.43 (62), 612.41 (14)	216 (100), 255(42), 314 (17), 538 (6)
hydroquinone	240.12 (100), 314.96 (21), 354.88 (17), 451.20 (28)	221 (100), 274 (5), 446 (14)

Figure 5a shows no difference between the spectrum recorded before and after the irreversible third plateau at 1.7 V. This suggests that the cause of this capacity is not due to a chemical change in the molecule but due to consumption of charge through an alternative, unwanted process e.g., hydrogen evolution.^44,48^

The general features of the DFT-derived and experimental UV–Vis spectra (Figure 5b) are similar for the electrochemical species present. However, the finer structure (as evidenced by the purple, middle-left spectrum) was not fully observed in the simulated spectrum, e.g., in the semiquinoidal state, which may be caused by the lower accuracy of the calculations when it comes to describing radicals. Overall, UV–Vis spectroscopic studies combined with DFT calculations served to support the proposed mechanism of two single-electron transfers during the voltammetric and galvanostatic experiments. A clear intermediate was observed, and the third plateau (not always observed) was identified to be the result of water reduction,^44^ or a similar parasitic capacity loss.

## NMR and EPR Spectroscopy

**Figure 6:**
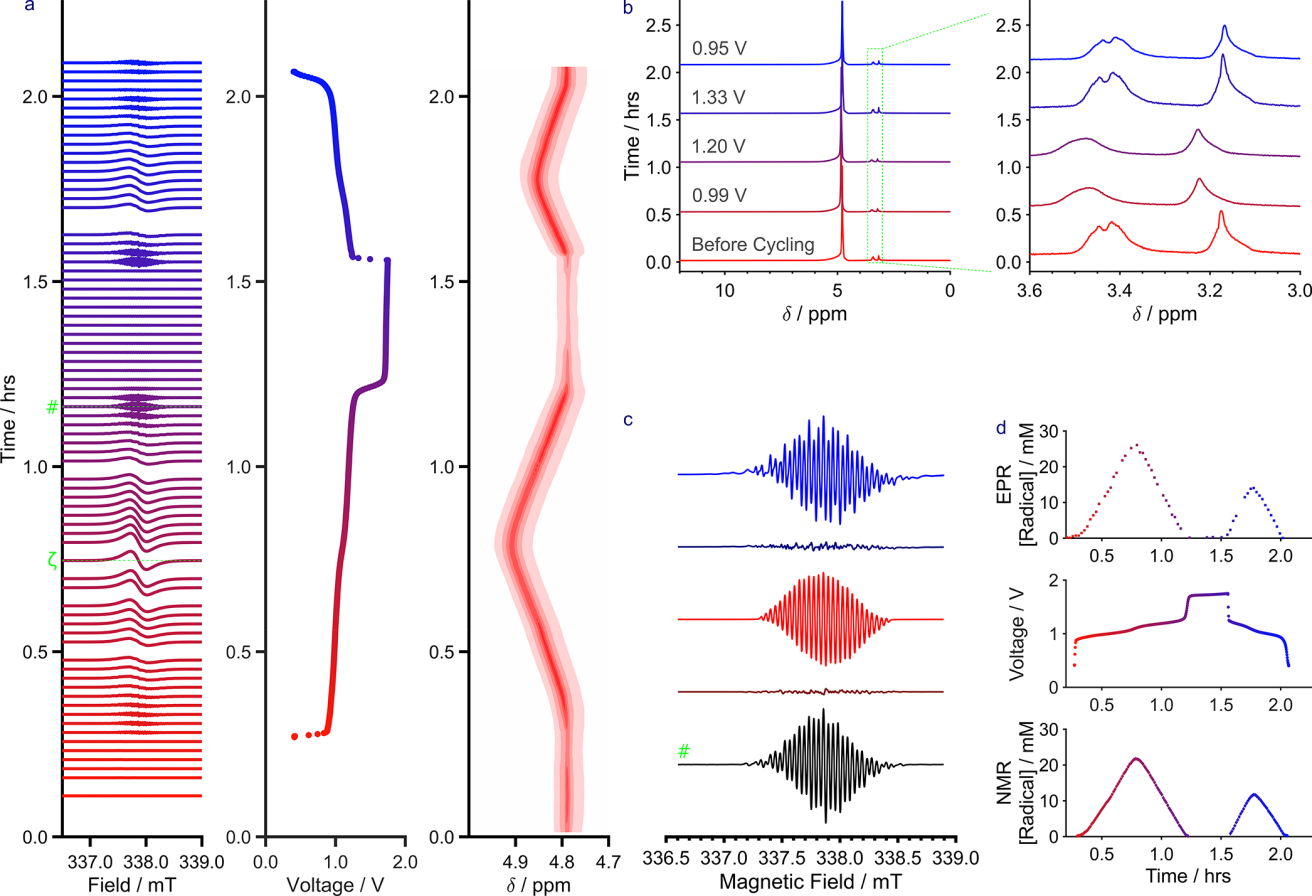
(a) In situ NMR/EPR from a lab-scale RFB of **2** against potassium ferrocyanide in D_2_O with 1 M LiOH added as the supporting electrolyte at ambient temperature under an inert nitrogen atmosphere. The battery was operated between 0.40 and 1.75 V at a current of 30 mA (6 mA·cm^–2^). Left to right: the graphs display the EPR spectra, the GCPL electrochemical performance, and the NMR (298 K, 300.13 MHz) signal of the water peak over time. The green ζ and # labels denote where slices of the EPR spectra were extracted and modeled in the SI, and (c), respectively, (b) in situ ^1^H NMR spectra selected at regular intervals spanning the duration of the first charge and discharge cycle plotted separately for clarity. Zoomed-in spectra are also provided corresponding to the impurity peaks observed at 3–3.6 ppm. (c) Normalized EPR spectra extracted at the end of charge (#, just before end of the second plateau). The experimental spectra (black) are compared to a simulated spectrum under an isotropic tumbling regime (red) and under the fast-motion regime (blue) with an anisotropic *g*-tensor ([2.0025 2.0024 2.0028]) and with slower tumbling (10^–7.98^ s). The residuals for both simulations are shown underneath the simulated spectra. (d) Proportion of radicals calculated from in situ NMR (bottom) and EPR data (top).

As highlighted in Figure 5, the first charging plateau forms the radical trianionic semiquinone, so we investigated the electron paramagnetic resonance (EPR) behavior of the system under battery operation to gain insight into delocalization of the unpaired electron. Figure 6a shows the EPR spectra alongside the GCPL profile and ^1^H NMR shift of the water peak. As discussed previously, the change in shift of the latter is ascribed to a bulk magnetic susceptibility or BMS shift, which is a measure of the change in radical concentration in the solution (this is the basis of the Evans method of determining the volume susceptibility of a liquid).^44,46,48−51^ Three plateaus are observed during the charge profile, and two are observed during discharge. As before, the first two are assigned to the two single-electron reduction processes of **2** (as ferrocyanide is correspondingly oxidized), while the third is attributed to water reduction.^44^ No changes in either the NMR (Figure 6a,b) or EPR spectra (Figure 6a) were observed during this third plateau (as also seen for the UV–Vis in Figure 5), and no changes were observed between the NMR spectra before and after the first charge and discharge (Figure 6b), with only water and impurity peaks being observed during the in situ experiment. The ex situ ^1^H NMR data taken after battery cycling (Figure S41) showed only a few very weak peaks in the baseline in addition to those observed from the in situ experiment. However, these peaks are of a much greater intensity in the catholyte tank, which suggests that crossover followed by degradation via either interaction with hexacyanoferrate or due to the much higher potentials on the positive electrode may have taken place. This may also have occurred during the asymmetric cycling discussed above (Figure 4), although the additional peaks in the ex situ data in Figure S30 are of a much lower intensity than those observed in Figure S41, despite the greater number of cycles, which suggests that the difference in purity between the samples used may be the major deciding factor. This may explain the enhanced capacity fade rate during the in situ experiment (Figure S36), although this may also be attributed to the capacity-consuming third plateau. Nonetheless, as before, the presence of similar peaks in the NMR spectra of both tanks is a clear sign of crossover.

Figure 6a also shows that as the battery was charged and radical species began forming, the EPR trace demonstrated several complex hyperfine interactions. A concomitant broadening and shifting of the NMR signals (BMS shift) are also observed in Figure 6b as the concentration of radicals increases. However, as the concentration of radicals increased, the EPR profile simplified as the spectra were affected by both the concentration and electron–electron exchange between radical species, which resulted in spectral broadening. The reduction in hyperfine coupling and increase in the intensity of the EPR signal continued until the end of the first plateau, before beginning to diminish. As the second plateau progressed, the radical species were quenched (semiquinone to hydroquinone) and the EPR signal underwent the opposite transformation, decreasing in intensity but increasing in complexity over the duration of the plateau. The low-concentration EPR trace was best resolved toward the end of the second plateau, where the radical concentration was low but there were sufficient radicals present to provide a good signal-to-noise ratio.

Modeling of the low-concentration signal (#, Figure 6a) where the hyperfine splitting could be resolved suggested that the unpaired electron was coupled to six different nitrogen atoms with distinct hyperfine coupling constants (Figure 6c, red, *g* = 2.0030, A = 5.81, 3.51, 2.24, 1.36, 1.14, 1.00 MHz, see Table S11). Fits with fewer numbers of distinct N atoms gave poorer simulations of the experimental spectrum, contrary to what might be expected from symmetry arguments, which would predict either two or four distinct N atoms. Moreover, the simulated result does not match the result predicted from DFT performed on the fully deprotonated triply charged anion where one would expect **2** distinct hyperfine values in a ratio of 2:1 (see Figure S36). The fact that six distinct hyperfine values are observed suggests that the system is much more complex than it initially appears and that perhaps asymmetric protonation (e.g., protonation of one triazole ring) occurs (see Figure S37). A small improvement to the fit was obtained when accounting for *g*-anisotropy (Figure 6c, blue), with DFT suggesting a *g*-anisotropy of [−137.2 3032.3 3968.1] ppm relative to the free electron (Table S9). This suggests that the *g*-values along two axes are similar in magnitude while the third is distinct. Fitting, starting from an initial asymmetry of [2.00299 2.00300 2.00301] resulted in a final anisotropy of [2.0025 2.0024 2.0028] which demonstrated a similar trend to the *g*-anisotropy values predicted from DFT of the radical trianion (see Table S13 for more details). An even better fit to the spectrum is obtained if tumbling (rotation of the anion) in the intermediate regime is accounted for (Figure 6c, blue). However, while we appreciate that the fit to this spectrum may not be unique, we stress that attempts to fit the spectra with a simpler model (two or even four distinct N atoms) were not successful, strongly suggesting that protonation/interaction with Li^+^, and its role in lowering the symmetry of the anion/creating different N-environments needs to be considered (see SI for further details).

Attempts to model the broad spectrum observed at intermediate states of charge where a high concentration of radicals is present could not be fit with the same hyperfine coupling constants, and the smaller peak-to-peak separation could not be reproduced with a simple model. The broadening is likely a result of intermolecular interactions between the radicals, the exchange interactions broadening the signal, and washing out the hyperfine interactions (i.e., resulting in self-decoupling).

Direct double-integration of the EPR spectra and the change in bulk magnetic susceptibility from the change in the ^1^H NMR shift of the water, as outlined in a previous work,^50^ allow the radicals to be quantified (Figure 6d). This yielded an approximate value of 87% for the maximum concentration of radicals in the sample during the first charge cycle. Capacity was lost during the third plateau at 1.7 V, and consequently, the maximum radical concentration was reduced during the subsequent discharge. This could be because of an increase in pH from the loss of hydrogen, via the hydrogen evolution reaction (HER), which might affect the solubility or stability of **2**, or may be due to the catholyte tank becoming charge limiting during discharge due to overconsumption of electrons from the ferrocyanide tank.

## Symmetric Cell

Having explored the full-cell performance and behavior of **2**, a symmetric RFB cell experiment was also carried out to identify the capacity fade rate and Coulombic efficiency in the absence of the catholyte. A potassium ferrocyanide solution was used to charge up a solution of **2** before the catholyte was replaced with a fresh (oxidized) tank of **2**, against which the charged (reduced) tank could be run.

**Figure 7:**
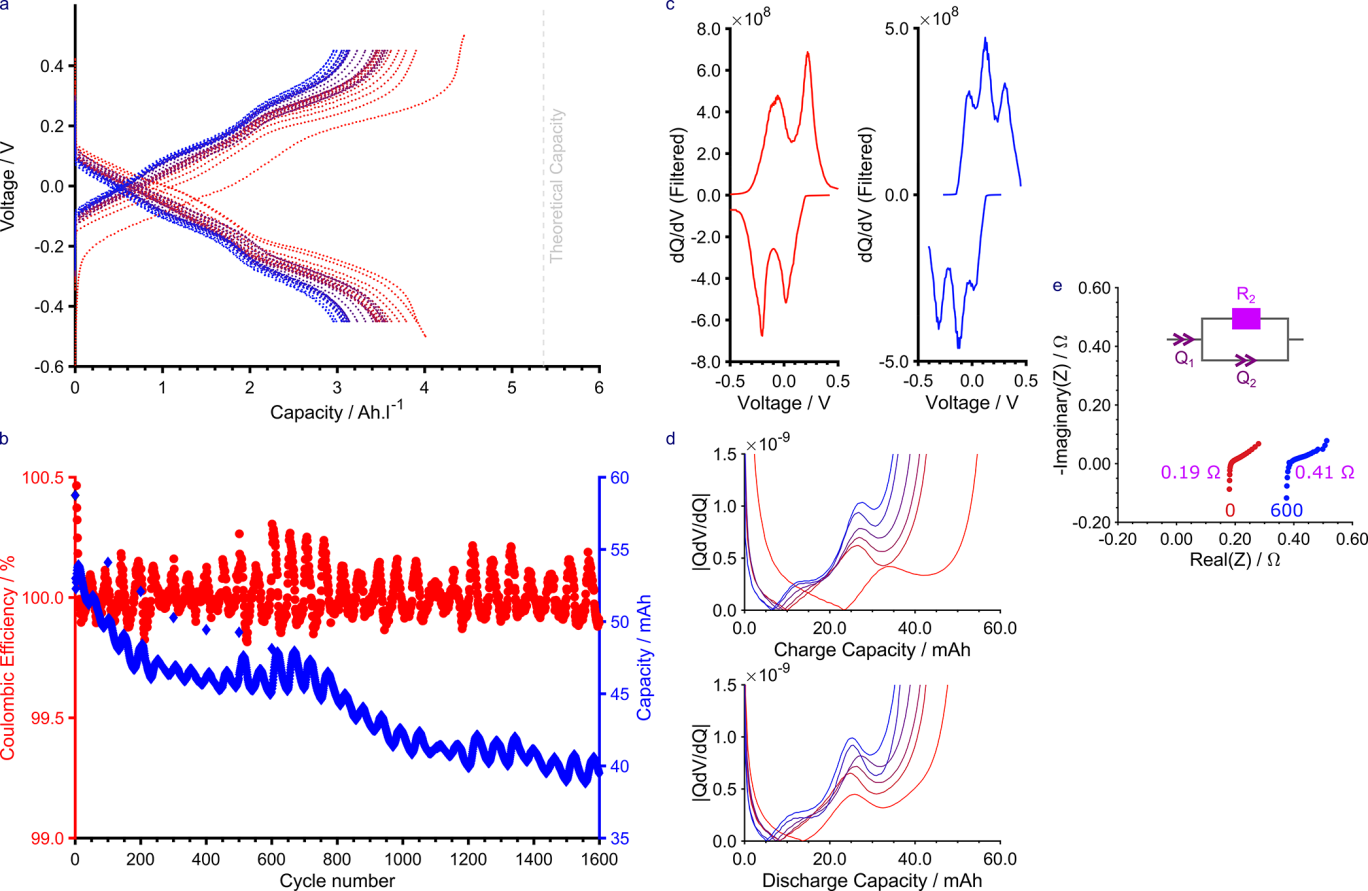
Lab-scale symmetric RFB performance of **2** in D_2_O with 1 M LiOH added as the supporting electrolyte at ambient temperature under an inert nitrogen atmosphere. The battery was operated between −0.45 and 0.45 V at a current of 200 mA. For clarity, only every 50th cycle is shown. (a) Voltage vs capacity. (b) Coulombic efficiency vs cycle number and capacity vs cycle number. (c) d*Q*/d*V* from the first cycle while the bottom-right shows the d*Q*/d*V* from the last cycle. (d) *Q *d*V*/d*Q* during charge (above) and discharge (below) against capacity for cycles 1, 300, 600, 900, 1200, and 1500. (e) Electrochemical impedance spectroscopy taken before (0) and after 600 cycles of battery cycling. The equivalent circuit shown was used to fit the data, and the resistances, corresponding to *R*_2_, are denoted in purple.

Figure 7a shows the galvanostatic cycling of the symmetric cell. The first cycle achieved 83% of the theoretical capacity, while the second reached 75%. The capacity fade rate, averaged over all of the cycles, was found to be 0.00659 mA h per cycle (0.0124% per cycle) with a CE of 99.98 ± 0.02% (Figure 7b). A diurnal cycle was observed to be present in the data with the capacity and CE oscillating over the course of ∼24 h. It is unclear whether this was caused by the temperature or light, but the former is more likely to be responsible. The increase in CE in comparison to the asymmetric cell (Figure 4) performance may be because of reduced crossover or may suggest that ferrocyanide may have been at least partly responsible for this previous lowering in efficiency (see Figure S13).

d*Q*/d*V* analysis was carried out (Figure 7c) on the data which showed that at the beginning of the experiment, two processes are involved during charge and discharge (Figure 7c, left). However, by the end of cycling, three processes were observed (Figure 7c, right). The third process grows after ∼300 cycles and gradually becomes the prominent plateau of the three during both oxidation and reduction. Moreover, during discharge, the hydroquinone-to-semiquinone transition (HQ-to-SQ, first plateau) diminishes in intensity as the second plateau becomes more intense.

As crossover in a symmetric cell experiment does not lead to permanent capacity fade, the cause of the overall capacity fade in this experiment is likely to be attributed to two factors: cell imbalance and degradation of the active material. The first argument is evident from the d*Q*/d*V* plot calculated from the final cycle, which, accounting for the Ohmic overpotential, suggests that there is a process that occurs at 0 V. This is further observed through the *Q* d*V*/d*Q* analysis (Figure 7d), which demonstrates not only a decrease in capacity over time but also the growth of an additional electrochemical transition point in the later cycles. The electrochemical process that develops over time could be the result of a change in the molecular structure of **2** and the formation of a redox-active degradation product, but as this process is at 0 V, a cell imbalance is more likely to be responsible.

Some active material loss to the membrane was evident from the postmortem analysis of the cell (see Figure S43) which showed discoloration and fouling of the membrane, corresponding to an increase in impedance (Figure 7e). Due to this contamination, the resistance of the cell increased over time, as the ionic conductivity of the membrane was reduced by pores and channels becoming blocked. However, this alone would not lead to the capacity fade observed (Δ*iR* = 0.044 V, ΔCapacity ≈ 0.04 A h·l^–1^). The capacity fade could be due to imbalances in the cell chemistry (as highlighted above), resulting in slippage in the voltage profiles. Although, degradation of the electrolyte may also be present. Postmortem NMR analysis of both tanks (Figure S45) showed several low-concentration peaks in the ^1^H NMR (where no signals are expected). As before, these minor peaks may be due to residual impurities from the synthesis of **2**, as one would expect much more intense peaks if significant molecular degradation were occurring over the course of cycling. Only a single peak was observed in the ^13^C NMR, however, and this may again be attributable to the low natural abundance of this nucleus. Analysis of the hexacyanoferrate tank used to initially charge the symmetric cell exhibited only one signal in the ^1^H NMR (where none was expected) suggesting again that the additional peaks observed previously (see Figure S30) from asymmetric cycling could have originated from **2** and its impurities crossing over. Any degradation product that was present in the system was therefore likely to be present in lower concentrations than the synthetic impurities, which were themselves also present only in very low concentrations.

Overall, the improved capacity retention and CE during symmetric cycling imply that hexacyanoferrate may enhance any degradation or deactivation of **2** (as suggested by Figures S30 and S41), which in turn further emphasizes the need for more robust and selective membranes to facilitate electrolyte exploration work and isolate degradation in the absence of crossover. In this regard, use of more selective membranes based on polymers of intrinsic microporosity (PIMs)^40,52−54^ may be advisable for any future exploratory work on these compounds.

## Conclusions

A series of nitrogen-rich heterocyclic quinones was synthesized from tetra-amino-benzoquinone and characterized. Sodium 4,8-dioxo-4,8-dihydrobenzo[1,2-*d*:4,5-*d*′]bis([1,2,3]triazole)-1,5-diide (**2**), 4*H*,8*H*-benzo[1,2-*c*:4,5-*c*′]bis([1,2,5]thiadiazole)-4,8-dione (**3**), and 2,6-dimethylbenzo[1,2-*d*:4,5-*d*′]diimidazole-4,8(1*H*,5*H*)-dione (**4**) were investigated in basic media. Of these three frameworks, **2** appeared to be the most promising, followed by **4**, while **3** was found to be incompatible with basic media. Pyridine (2,6-di(pyridin-2-yl)benzo[1,2-*d*:4,5-*d*′]diimidazole-4,8(1*H*,5*H*)-dione (**5**) and 2,6-di(pyridin-3-yl)benzo[1,2-*d*:4,5-*d*′]diimidazole-4,8(1*H*,5*H*)-dione (**6**)) and pyridinium-incorporating compounds (2,2′-(4,8-dioxo-1,4,5,8-tetrahydrobenzo[1,2-*d*:4,5-*d*′]diimidazole-2,6-diyl)bis(1-methylpyridin-1-ium) diiodide (**7**), 4,4′-(4,8-dioxo-1,4,5,8-tetrahydrobenzo[1,2-*d*:4,5-*d*′]diimidazole-2,6-diyl)bis(1-methylpyridin-1-ium) diiodide (**8**) and 3,3′-(4,8-dioxo-1,4,5,8-tetrahydrobenzo[1,2-*d*:4,5-*d*′]diimidazole-2,6-diyl)bis(1-methylpyridin-1-ium) diiodide (**9**)) were also synthesized and evaluated in the hope of introducing beneficial electrolyte functionalities. However, due to their insolubility in basic media, acidic media was used to assess their electrochemical performance. The pyridinium-incorporating compounds (**7**–**9**) performed well at the CV scale but owing to their poor solubility (at <50 mM) much greater optimization of the framework is needed before any further investigations can be carried out. The most promising compound, **2**, was tested as a potential RFB electrolyte in a lab-scale flow battery against potassium ferrocyanide. This is the first study of **2**, or any related heterocyclic quinone, as an anolyte in aqueous RFBs. **2** has a low reduction potential of −0.68 V(SHE), high stability against hydroxide ions in the long-term CV experiments, and capacity fade rates of 0.35% and 0.0124% per cycle in the asymmetric and symmetric RFB experiments, respectively. Capacity fade was attributed to crossover and cell imbalance, although molecular degradation may also have contributed.

In situ UV–Vis and EPR spectroscopy, supported by DFT calculations, of **2** confirmed the species being formed as first the radical trianion (first plateau), then the fully reduced hydroquinone analogue (second plateau) during the two one-electron reduction processes during cycling. EPR, in tandem with NMR (via the change in bulk magnetic susceptibility of the water resonance), also allowed quantification of the peak-radical concentration (87%) during the first charge cycle. Importantly, no changes were observed in either the in situ NMR, EPR, or UV–Vis spectra to indicate molecular degradation during any of the charge plateaus. Based on this, the capacity-consuming third plateau at 1.7 V was attributed to the electrolysis of the solvent. Modeling of the EPR spectrum at low concentrations suggested that the radical, **2**^–•^, was capable of delocalizing across all six nitrogen atoms, which may be important for stability.

During battery cycling, visual discoloration of the initially colorless membrane along with an increase in cell-impedance and gradual loss of conductivity occurred, as both **2** and its breakdown products became trapped during passage through the membrane. Similar peaks were observed from postmortem ^1^H and ^13^C NMR of both catholyte and anolyte tanks suggesting that the crossover through the membrane was responsible for the capacity fade in this system, leading to membrane fouling (and enhanced degradation of **2** on the catholyte side). However, NMR analysis of the electrolyte tanks was inconclusive as to the nature of the molecular degradation due to both the presence of low-concentration impurities from the synthesis of **2** and the absence of any reporter nuclei in the molecular framework. As such, further work regarding identifying and mitigating any potential degradation pathways of **2** is still required, and ^13^C or ^15^N enrichment of **2** may aid in providing a clearer answer, as any degradation product that was present in the system herein was likely to be present in lower concentrations than the synthetic impurities. Currently, these motifs still require further synthetic exploration to increase their solubility, and most likely stability, before they can be considered viable for industrial flow battery applications. In this direction, further work regarding cell-level optimization is also needed, not only with regards to identifying more selective membranes, but also with regards to exploring alternative supporting electrolyte salts, additives, and battery cycling protocols to ensure that the maximum capacity can be achieved. For example, exploring the effect of cations on **2** may lead to a decreased capacity fade rate, as tetra-methyl-ammonium was successfully shown to do for 2,6-dihydroxyanthraquinone.^55^ Nevertheless, this work represents the first foray into synthetic design for redox flow batteries using this family of anolytes.

## Supporting Information Available

Additional experimental details, materials, methods, and calculated structure coordinates (PDF)
DW_B2_0304 (CIF)

## Supplementary Material

ae3c02223_si_001.pdf

ae3c02223_si_002.cif
