# Supplementary material for: Exploring the Landscape of Heterocyclic Quinones for Redox Flow Batteries
Source: ACS Appl Energy Mater. 2023 Dec 28;7(2):414–26. doi: 10.1021/acsaem.3c02223 (PMC10806605; doi:10.1021/acsaem.3c02223)
Supplement: Supplementary file 1 — ae3c02223_si_001.pdf [file ae3c02223_si_001.pdf]

Supporting Information: Exploring the Landscape of Heterocyclic Quinones for Redox Flow Batteries

Rajesh B. Jethwa,[a,b] Dominic Hey,[a] Rachel N. Kerber,[a,c] Andrew D. Bond,[a]

Dominic S. Wright,[a] and Clare P. Grey*[a]*

[a]Yusuf Hamied Department of Chemistry, University of Cambridge, Lensfield Road, Cambridge, CB2 1EW, UK

[b] Present Address: Institute of Science and Technology Austria, Am Campus 1, Klosterneuberg, Niederösterreich, 3400, Austria

[c] Present Address: Johnson Matthey Technology Centre, Blounts Ct Rd, Sonning Common, Reading, RG4 9NH, United Kingdom

Corresponding Authors:

Clare P. Grey: cpg27@cam.ac.uk

Dominic S. Wright: dsw1000@cam.ac.uk

Synthetic Procedures

The syntheses described in this work were carried out in a fume-hood or under an inert-atmosphere where necessary. For inert-atmosphere work, a Schlenk line (double manifold) was used under either nitrogen or argon. The inert gas was dried over activated silica gel, activated molecular sieves, and potassium hydroxide. The Schlenk flasks were evacuated to ~10 Pa and refilled with an inert atmosphere three times before charging. The glassware was cleaned in a potassium hydroxide/isopropanol bath and a hydrochloric acid/water bath, followed by rinses with deionised water and distilled acetone. The glassware was then pre-dried at 100 °C before use. Any liquid reagents and solvents were added through a rubber septum (Suba-Seal®) using a purged and dried syringe while the flask was under a positive pressure of inert gas. The flasks were pre-filled with solids where possible, and any subsequent addition was carried out rapidly under a positive pressure of inert gas.

Elemental Analysis

Microanalysis of carbon, nitrogen, and hydrogen was carried out on an Exeter CE-440 Elemental Analyser. The samples provided were accurately weighed by the analyst prior to measurement.

X-ray Diffractometry

X-ray data were collected on a Bruker D8-QUEST diffractometer, equipped with an Incoatec IμS Cu microsource (λ = 1.5418 Å) and a PHOTON-III detector operating in shutterless mode. Crystals were mounted on a MiTeGen crystal mount using inert polyfluoroether oil and the analysis was carried out under an Oxford Cryosystems open-flow N_2 Cryostream. The control and processing software was Bruker *APEX3*. The diffraction images were integrated using *SAINT* in *APEX3*, and a multi-scan correction was applied using *SADABS*. Structures were solved using *SHELXT*,[1] and refined using *SHELXL*.[2]

Mass Spectroscopy

Via the departmental service, samples were analysed using a Waters LCT Premier spectrometer. Electrospray ionisation was used to generate a charge on the sample, and a time-of-flight probe was used.

NMR Spectroscopy

Samples were prepared in deuterated solvents (used as received) in 528-PP NMR tubes. Spectra were recorded on a Bruker 400 MHz Neo Prodigy Spectrometer (400.13 MHz for ^{1}H, 100.61 MHz for ^{13}C), a Bruker 400 MHz Avance III HD Spectrometer (400.03 MHz for ^{1}H,

100.59 MHz for ^{13}C), or on a Bruker 600 MHz Avance Spectrometer (600.13 MHz for ^{1}H, 150.90 MHz for ^{13}C). The chemical shifts, δ ppm, for ^{1}H and ^{13}C NMR spectra are calibrated to the lock-solvent, relative to tetra-methyl-silane (TMS). All other nuclei were reported relative to ^{1}H using the standard absolute frequency ratio. The chemical shifts from residual solvents were assigned to the known literature values.[3]

IR Spectroscopy

Infrared spectroscopy was carried out on a Perkin-Elmer Spectrum One FTIR Spectrometer fitted with a Perkin-Elmer ATR sampling accessory. The crystal was cleaned with acetone and ethanol, and a background was run prior to measurement.

Electrochemistry

Note: D_2O, rather than H_2O, was used as the solvent throughout all electrochemical studies to enable NMR analysis without interference of a large solvent peak and to keep the solvent consistent with the galvanostatic cycling data presented in this work.

Cyclic Voltammetry

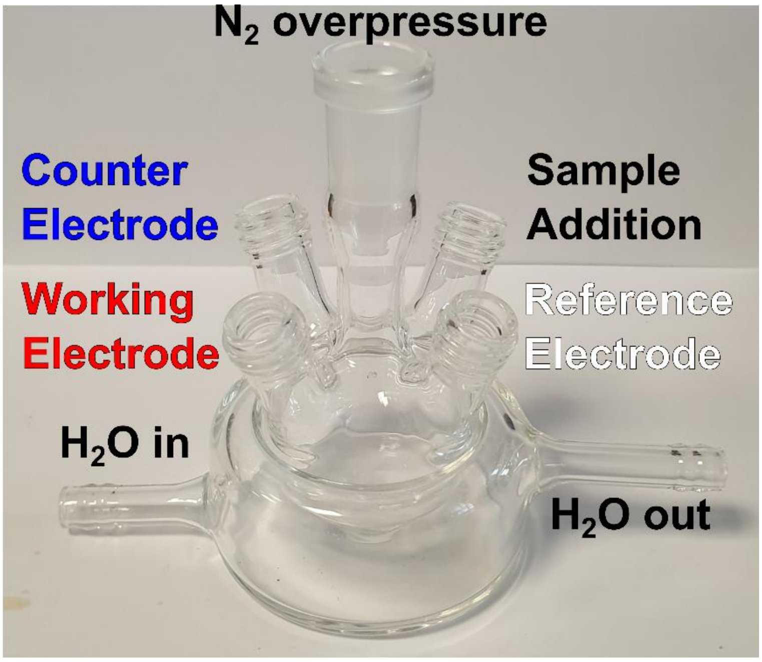

Figure S 1: Image of the in-house designed aqueous CV cell.

Cyclic voltammetry (CV) experiments were carried out using three-electrode cells designed in-house. Aqueous measurements were carried out using the cell shown in Figure S 1. A Thermo Haake DC50-K75 circulator was used to ensure that the voltametric cell was at 25 °C and GL14 screw threads and caps, in combination with rubber O-rings, were used to ensure a tight seal

around the electrodes. A rubber septum (suba-seal®) was used to close the remaining opening, and for addition and removal of samples under inert atmosphere. The cell was flushed with nitrogen prior to measurement and kept under an overpressure of the inert gas during measurement using the ground-glass opening at the top of the cell. The nitrogen was dried over a desiccant column before use.

All voltametric experiments used a glassy carbon working electrode embedded in a PEEK housing (Biologic A-012744, 3 mm) and a coiled platinum counter electrode (1.00 mm diameter). The glassy carbon was polished using 0.05 µm polishing alumina paste (Biologic A-001050) on alumina polishing pads (Biologic A-001040), and where necessary using 1 µm polishing diamond paste (Biologic A-002054) on diamond polishing pads (A-001041) with Millipore deionised water. 3x 100 cycles of figure-eight[4] were used to polish the working electrode, with intermediate sonication steps of one second duration followed by drying under nitrogen gas. The platinum was purchased from Sigma-Aldrich and cleaned using nitric acid and deionised water before use.

Biologic SP-150, VSP 200, and Ivium Vertex potentiostats were used, with the proprietary software, for the experiments described herein.

Electrolyte and supporting electrolyte solutions were prepared under inert atmosphere, avoiding the use of vacuum grease where possible, prior to measurement. A background CV was run on the supporting electrolyte solution to ensure that it was sufficiently free from contamination prior to use. 20 mVs^{-1} was chosen as the comparison scan-rate, providing a compromise between duration of the experiment and sufficient time to allow relaxation and separation of the different redox processes. Cyclic voltammetry was run over long time periods where possible to investigate the stability of the analyte against the supporting electrolyte fully, and to observe if alternative detrimental processes, such as electrode deposition, occurred. Buffered systems were not used to keep the supporting electrolyte systems as simple as possible, and optimisation of pH was outside the scope of this work. Moreover, where multiple redox processes were observed, voltametric isolation and investigation of individual redox couples was not carried out.

Galvanostatic Cycling

Galvanostatic cycling (two-electrode setup) was used as it is the standard method by which flow battery electrolytes or battery electrodes are tested. Under the application of a current, the voltage of the battery changes and any redox processes are presented as plateaus or slopes in

the voltage profile of the system. These can further be identified by taking the derivative of the charge with respect to the voltage and plotting this against the voltage, i.e., dQ/dV vs. V. In this work, a typical charging current of 200 mA (40 mA.cm^{-2}) was used for all battery performance tests, i.e., 200 mA current was applied during charge and -200 mA current was applied during discharge up to the limiting potentials of the experiment. Varied current experiments were also carried out on promising samples, varying the current from low–to–high before returning to a lower, previously tested current. Note that where impedance data was collected during these experiments, measurements were taken during the discharged state.

Redox Flow Battery (RFB) Setup

The anodised aluminium 5 cm^2 active-area flow battery was purchased from Scribner Associates (Figure S 2). Gold-plated copper plates were compressed against serpentine-patterned flow plates made from ultrahigh-purity graphite. Viton gaskets (0.7 mm) were then used to seal the cell with a 2 Nm torque applied to each bolt. The anodised aluminium was prone to corrosion in basic solution so direct contact was avoided by careful insertion of the inlet and outlet tubing through the Viton O-rings.[5]

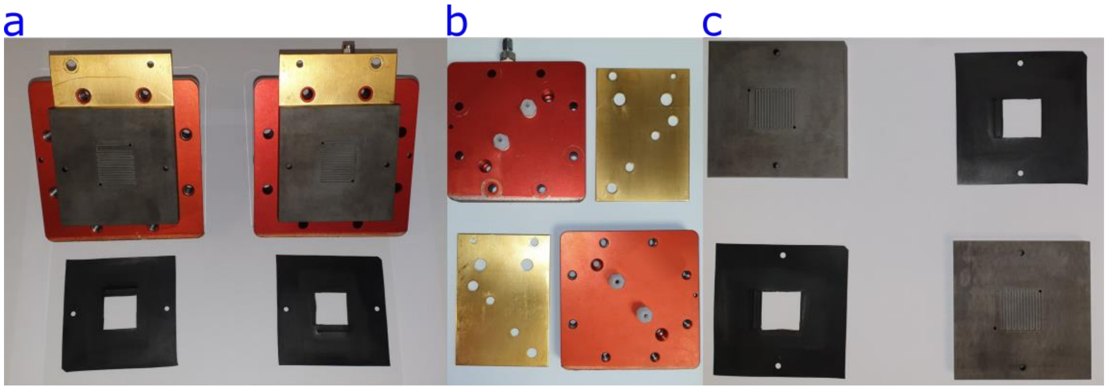

Figure S 2: Redox flow battery purchased from Scribner Associates. (a) Photo of each half of the stack, (b) Photo of the anodised aluminium endplates and the gold-plated copper current collectors, (c) Photo of the serpentine-flow field containing ultrahigh-purity graphite electrodes and the Viton gaskets.

Custom-made glass Schlenk flasks were used to house the electrolyte. The J. Young's tube joints were used to ensure a closed system under inert atmosphere without silicon grease contamination and for ease of isolation of the tanks after cycling, especially where the species may be prone to rapid discharge or degradation in the presence of oxygen. The design of the tanks also allowed easy handling under inert atmosphere on a double-manifold.

The solutions were cycled using peristaltic pumps (MasterFlex® L/S® 0775-10, Cole-Parmer). Perfluoro-alkoxy-alkane (IDEX, PFA Natural 1/16" OD, 0.040" ID) tubes were used in

conjunction with Chem-durance® L/S® Bio tubing for the peristaltic pumps to transport the electrolyte solutions. Polyether-ether-ketone (PEEK) fittings (Diba, Omni-Lok™, Inv. Cone, and barbed adapters) were used to connect the tubing together whilst maintaining an air-tight seal.

Carbon Paper Pre-treatment

Three layers of pre-treated SGL Carbon Gmbh Sigracet® GDL-39AA- Carbon (FuelCellStore) were used on each side of the RFB cell stack. The carbon felt was pre-treated by heating in air at 400 °C for 24 hours.[6]

Nafion 212 Pre-treatment

Nafion NRE-212 membranes (0.05 mm thick, >= 0.92 meq.g^{-1} exchange capacity, 31175-20-9) were used for all flow battery runs. The membranes were pre-treated by heating in deionised water (80 °C, 20 mins) before soaking in hydrogen peroxide solution (5%, 35 mins). The membranes were then stored in a stock solution (0.1 M) of the supporting electrolyte at room temperature.[6]

In situ setups

EPR

A benchtop EPR spectrometer (MS5000, Magnettech) was positioned outside of the 5 G line of an accompanying NMR spectrometer. The electrolyte solution was pumped through the flow battery, then through the EPR magnet and then either back to the electrolyte reservoir or onwards to the NMR magnet. The flow direction was from bottom-to-top and a flat EPR cell (E4503, Magnettech) was used to minimise heating of the sample by microwave irradiation. The cell was orientated in the EPR resonator so that the magnetic field strength was maximised, and the electric field was minimised. An in-house PEEK adapter was used to connect the EPR cell to the PFA (1/16”) transfer tubing. The volume of the cell in the excitation region of the microwave was 0.03 cm^3 (2.00 x 0.50 x 0.03 cm^3) and a residence time of 0.13 s was observed at a 13.6 ml.min^{-1} flow rate.[7] The experiments were carried out using a Biologic SP-150 potentiostat.

Simulation of the EPR spectra was carried out using Easyspin.[8]

NMR

A custom-made NMR sampling tube (14 cm length, 10 mm OD, medium wall) was placed inside a micro-imaging probe (Bruker 2.5). The electrolyte solution was pumped either directly from the flow cell or from the EPR spectrometer (as described above), flowed from bottom–

to–top to ensure sufficient filling. The inlet and outlet were connected to two PFA tubes (1/16” OD, 0.5 mm ID, 3 m). The electrolyte volume inside the sampling tube was 7.3 cm^3 with a detection volume of 1.57 cm^3 (3 cm detection length of the probe). Pseudo-2D NMR experiments were performed by direct excitation (90° pulse, $t_{acquisition}$ = 1.5 s).[5,7] The experiments were carried out using a Biologic SP-150 potentiostat.

Coupled EPR and NMR

When the EPR and NMR setups are combined, at a flow rate of 13.6 $ml.min^{-1}$, the timings are detailed in Table S 1.[7]

Table S 1: Time taken for the solution to move from one component to the other in the coupled in-situ EPR and NMR setup.[7]

Start	End	Time / s
Reservoir	Battery	3
Battery	EPR detection region	3
EPR detection region	NMR detection region	29
NMR detection region	Reservoir	29
Reservoir	Reservoir	64

UV-Vis

An in-house UV-Vis setup was used. A 100 µm optical path length flow cell (Starna Scientific, 45/TC/Q/0.1, nominal volume = 0.040 ml) was connected in the flow path of the anolyte. A deuterium-tungsten-halogen light source (DH-Mini Ocean Optics UV-Vis-NIR Fiber Optic Light Source) was then focused on the sample through a quartz UVFS Plano-Convex lens (Thor Labs, focal length = 40 mm). The transmitted light was collected using an Avantes SensLine spectrophotometer (AvaSpec-HS2048XL-EVO) and High OH UV-Vis optical fibers (Avantes, diameter = 200 µm) were used to transport and collect the light. The electrolyte was pumped at a speed of 80 rpm (25 $ml.min^{-1}$).

Potentiostatic Electrochemical Impedance Spectroscopy

Impedance spectroscopy was carried out under the potentiostatic regime for both voltametric experiments and galvanostatic cycling experiments. The experiments were carried out averaging across two measurements using an oscillation of amplitude 10 mV (V_{rms} ~ 7.07 mV) and a wait period of 0.1 s. The sweep range was from 200 kHz to 100 mHz with six points per decade in logarithmic spacing. The experiments were carried out using a Biologic VSP-200 potentiostat.

DFT Calculations

Density functional theory (DFT) calculations were carried out using the in-house Odyssey HPC cluster on the Gaussian 16 code.[9] Molecular models were taken from single-crystal X-ray diffraction results where possible or an initial estimate was made based on the chemical structure. The models were geometrically initially optimised at the PBE[10,11]/TZVP[12,13] level of theory with empirical dispersion (GD3).[14] The optimised geometry was then used as an initial estimate for further optimisation at the B3LYP[15]/TZVP level of theory, again with dispersion corrections. The optimised structure was then taken as an initial estimate for calculation with implicit solvation (PCM)[16] at the B3LYP/TZVP level of theory. The optimised structure was then used for single-point calculations to estimate the energy change upon solvation, ΔE_{solv} (SMD implicit solvation model)[17] and to estimate the NMR chemical shifts if necessary. Frequencies were checked to ensure that the structures had successfully reached the ground state (as opposed to a transition state or saddle point). The Gibbs Free Energies were then used to estimate the reduction potentials by conversion using the Nernst Equation.[18] As the strength of DFT is in identifying trends, rather than for calculating absolute values, these potentials were used to estimate how changes in the chemical structure may affect the experimental observations.

The hybrid functional B3LYP was chosen due to its wide and established use for organic molecules. The TZVP basis set was chosen as a compromise between accuracy and cost.

The EPR-III[19] basis set was used in combination with B3LYP for predicting the hyperfine coupling constants of semi-quinoidal species and CAM-B3LYP[20] was used for time dependent (TD)[21–27] DFT to predict the UV-Vis spectra of species.

Summary

Table S 2: Summary of potentials, diffusivities, solubilities, and energy densities of the species explored in this chapter. † denotes that this is attributed to this work while the use of red, green and blue fonts denote the use of acidic, neutral or basic solutions, respectively.

Species	Potential / V(SHE)	Solvent	Diffusivity / 10^{-6} cm^2s^{-1}	Solubility / mM	Theoretical Capacity / $Ah.l^{-1}$	Reference
**1**	0.43, -0.85	DMF	/	/	/	[28]
**2$_{Li}$**	-0.516, -0.681 (-0.542, -0.688)	H_2O	7	(>442)	(>23.6[29])	†([30])
**2$_{Na}$**	-0.550, -0.713	H_2O	/	0.4	0.02	[30]
**2$_{K}$**	-0.559, -0.718	H_2O	/	35	1.87	[30]
**2$_{TMA}$**	-0.484, -0.732	H_2O	7	/	/	†
**2$_{TPA}$**	-0.545, -0.853	H_2O	2	/	/	†
**2$_{TBA}$**	-0.628, -0.826	H_2O	/	/	/	[30]
**2$_{Li/TPA}$**	-0.528, -0.787	H_2O	3	/	/	†
**3**	-0.53, -1.15, -1.56 (-0.34, -0.86)	DMSO (H_2O)	/	/	/	†
**4**	-0.57 (0.56)	H_2O (H_2O)	0.4 (0.2)	26.5	1.42	†
**5**	0.40	H_2O	/	1.2	0.06	†
**6**	0.48	H_2O	/	0.6	0.03	†
**7**	0.67, 0.89 (-0.36, -0.57)	H_2O (H_2O)	90	13.5	0.72	†
**8**	0.67, 0.89 (-0.36, -0.54)	H_2O (H_2O)	40	17.1	0.91	†
**9**	0.68, 0.89 (-0.31)	H_2O (H_2O)	50	4.8	0.26	†

DFT Exploration of Structures

Table S 3: The corresponding reduction potentials and FMOs for each of the compounds studied using DFT methods (B3LYP/ TZVP).

	Structure	Reduction Potentials / V vs H_2	(R)HOMO / $kJ.mol^{-1}$	(O)LUMO / $kJ.mol^{-1}$
**1**	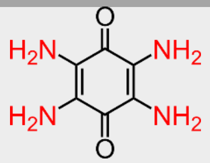	-0.04	-498.1	-300.4
$\mathbf{2}^{2-}$	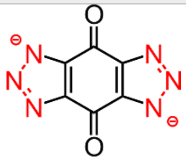	-0.33	-404.6	-235.1
$\mathbf{2}_{\mathbf{H}}$	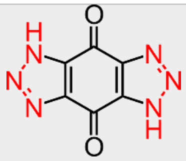	-0.07	-523.9	-347.8
$\mathbf{2}_{\mathbf{Li}}$	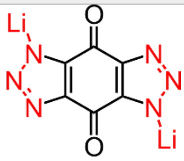	-0.19	-460.2	-330.7
$\mathbf{2}_{\mathbf{Na}}$	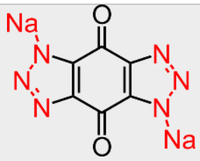	-0.25	-431.8	-268.4
$\mathbf{2}_{\mathbf{K}}$	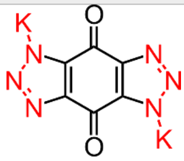	-0.29	-428.9	-254.0
$\mathbf{2}_{\mathbf{TMA}}$	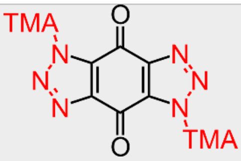	-0.25	-430.7	-222.6
$\mathbf{2}_{\mathbf{TEA}}$	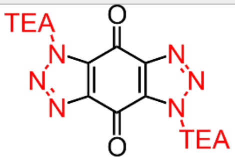	-0.20	-429.9	-263.0

	Structure	Reduction Potentials / V vs H_2	(R)HOMO / kJ.mol^{-1}	(O)LUMO / kJ.mol^{-1}
**2_{TPA}**	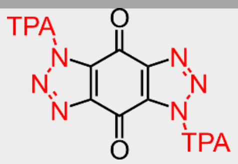	-0.22	-432.4	-276.5
**3**	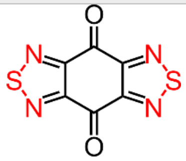	-0.03	-542.1	-356.7
**4**	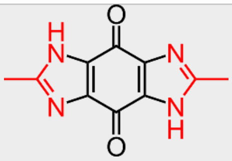	0.12	-505.0	-322.5
**4_{MeImid}**	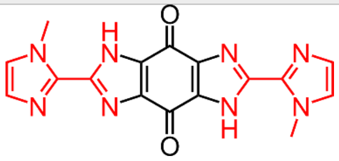	0.13	-514.2	-332.2
**5**	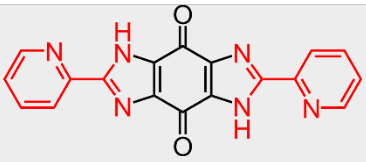	0.13	-515.8	-332.9
**6**	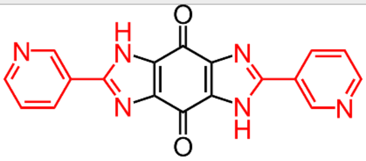	0.13	-520.2	-337.0
**8**	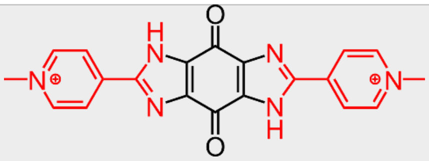	0.20	-551.2	-366.7
**8_{4Me}**	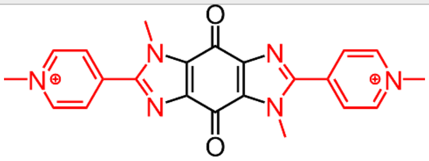	0.13	-536.0	-355.6
**8_{6Me}**	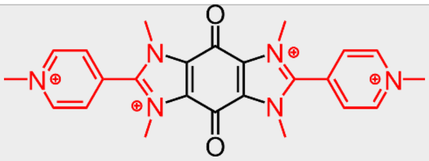	0.27	-677.5	-451.5

	Structure	Reduction Potentials / V vs H_2	(R)HOMO / $kJ.mol^{-1}$	(O)LUMO / $kJ.mol^{-1}$
**8_{0Me}**	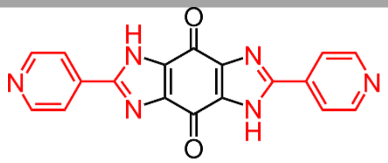	0.15	-525.1	-335.8
**7**	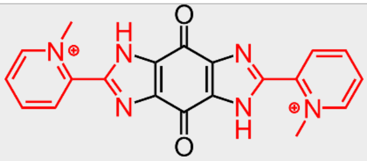	0.17	-557.4	-371.5
**9**	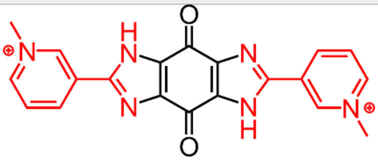	0.21	-543.8	-359.6

Cyclic Voltammetry

Basic Media

Sodium 4,8-dioxo-4,8-dihydrobenzo[1,2-d:4,5-d']bis([1,2,3]triazole)-1,5-diide (**2**)

*Table S 4: Half-wave potentials and peak-to-peak separations for the redox processes of **2** in basic aqueous media with various cations. The values denoted with an asterisk refer to those reported in reference [30].*

Cation	E_1 / V(SHE)	ΔE_1 / mV	E_2 / V(SHE)	ΔE_2 / mV	E_1-E_2 / mV
**Li^+**	-0.542* |-0.516	57* | 63	-0.688* | -0.681	86* | 122	146 | 165
**Na^+**	-0.550*	57*	-0.713*	88*	163
**K^+**	-0.559*	57*	-0.718*	106*	159
**TMA^+**	-0.484	95	-0.732	321	248
**TPA^+**	-0.545	83	-0.853	208	308
**TBA^+**	-0.628*	58*	-0.826*	86*	198
**$Li^+_{0.5}TPA^+_{0.5}$**	-0.528	76	-0.787	167	259

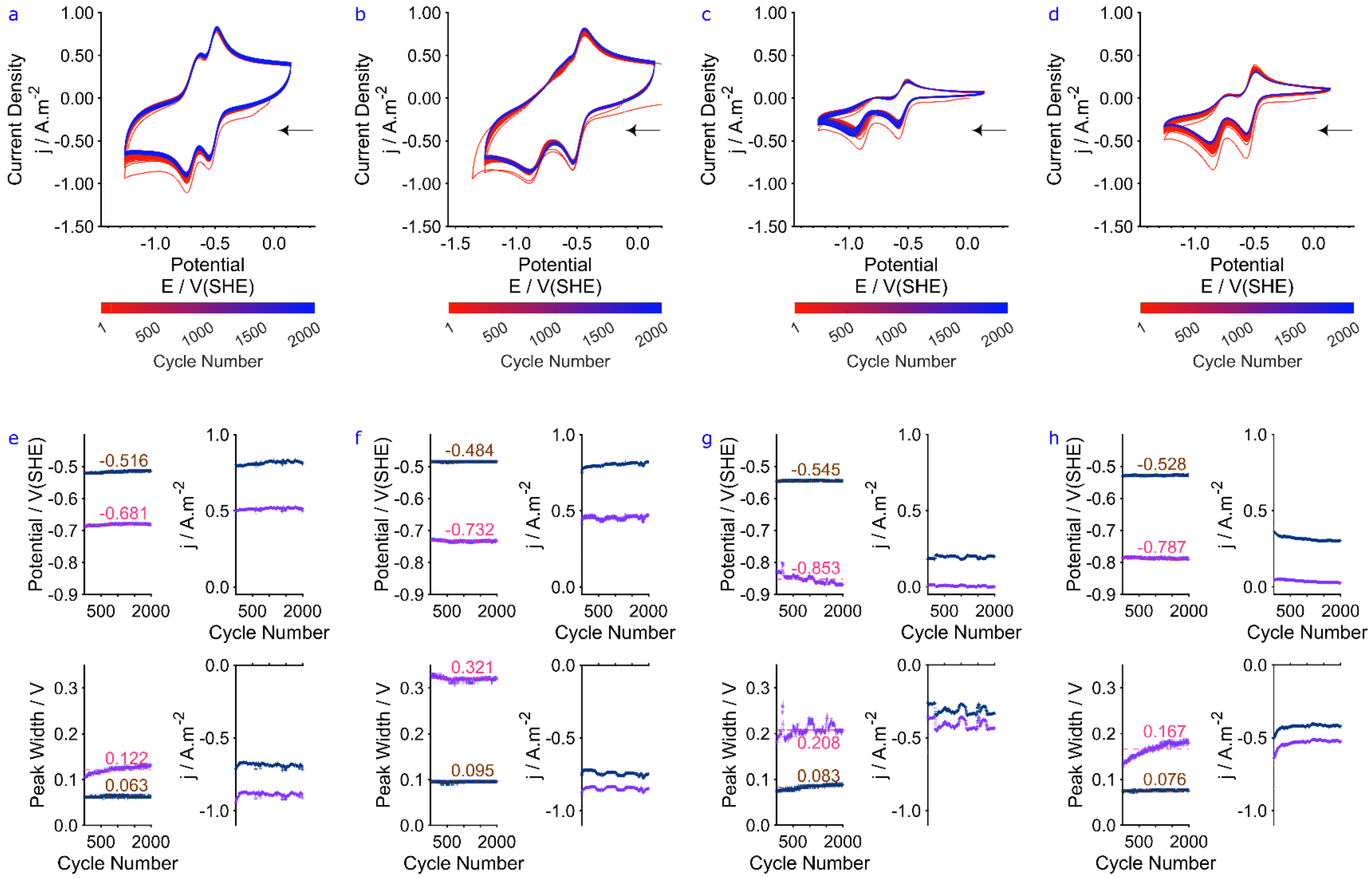

*Figure S 3: Cyclic voltammograms of a 1 mM solution of **2** in heavy water (D_2O) with (a) 1 M LiOH (b) 1 M TMAOH (c) 1 M TPAOH (d) 0.5 M LiOH, 0.5 M TPAOH as the supporting electrolyte. The scan rate was 20 mVs^{-1}, scanning towards negative potentials first. The experiment was carried out at 25 °C using a 3 mm diameter glassy carbon working electrode, coiled platinum wire reference electrode and a mercury-mercury oxide (1 M NaOH) reference electrode. By tracking the peaks, the average half-wave potential and peak-width were calculated for the different alkaline solutions and the raw currents extracted (e) 1 M LiOH (f) 1 M TMAOH (g) 1 M TPAOH (h) 0.5 M LiOH, 0.5 M TPAOH.*

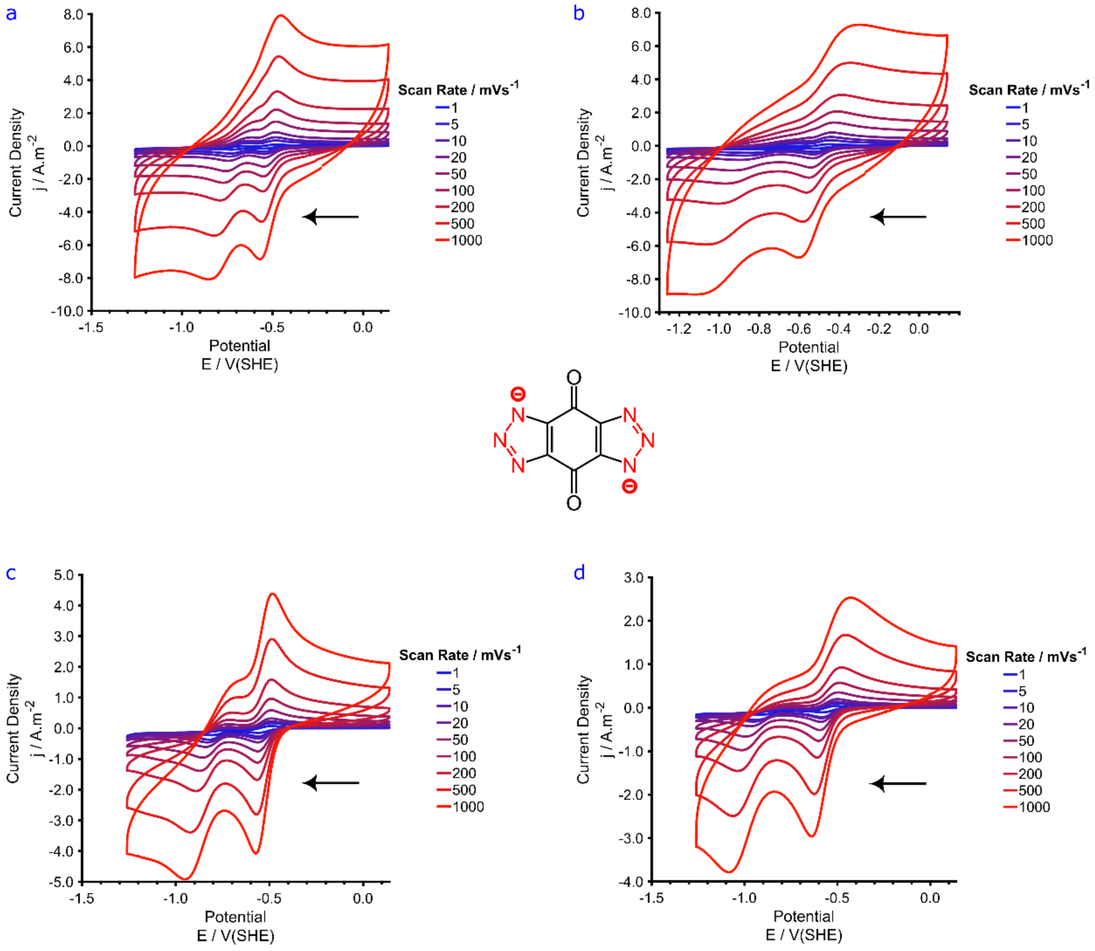

*Figure S 4: Cyclic voltammograms of a 1 mM solution of **2** in D_2O with (a) 1 M LiOH (b) 1 M TMAOH (c) 0.5 M LiOH and 0.5 M TPAOH (d) 1 M TPAOH as the supporting electrolyte. A range of scan-rates were used, scanning towards negative potentials first. The experiment was carried out at 25 °C using a 3 mm diameter glassy carbon working electrode, coiled platinum wire reference electrode and a mercury-mercury oxide (1 M NaOH) reference electrode.*

*Table S 5: Diffusivities of **2** calculated from the Randles–Ševčík and Levich analyses. The | for the Randles–Ševčík results denotes the different values calculated for the high (left) or low (right) potential redox events.*

**Cation**	**Diffusivity (Randles–Ševčík) / 10^{-7} cm^{2}s^{-1} (R^2)**	**Diffusivity (Levich) / 10^{-6} cm^{2}s^{-1} (R^2)**
**Li^+**	7 (0.9840)	8.28 (0.9788)
**TMA^+**	7 (0.9899)	7.95 (0.9898)
**TPA^+**	2 (0.9988)	1.31 (0.9842)
**$Li^+_{0.5}TPA^+_{0.5}$**	3 (0.9996)	

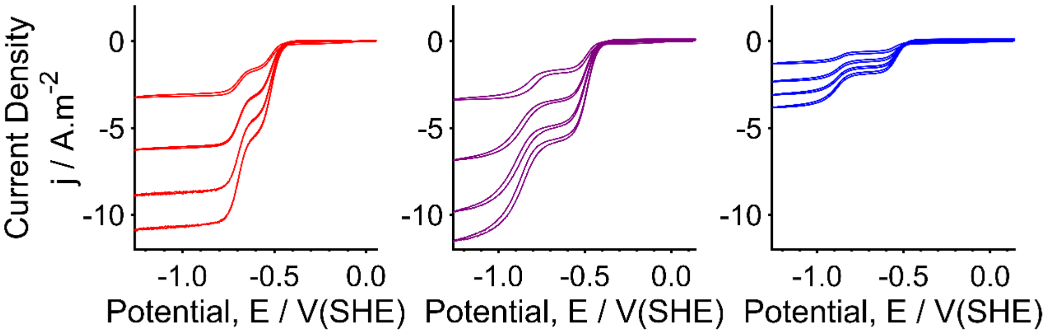

*Figure S 5: Cyclic voltammograms of **2** using a rotating-disk electrode a scan-rate of 1 mVs^{-1} in (red, left) 1 M LiOH, (purple, middle) 1 M TMAOH and (blue, right) 1 M TPAOH in D_2O with angular velocities of 100, 400, 800 and 1200 rpm.*

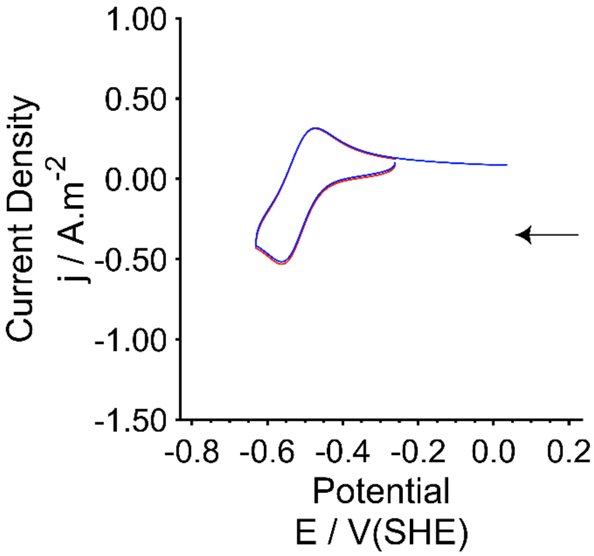

*Figure S 6: Cyclic voltammograms of a 1 mM solution of **2** in heavy water (D_2O) with 1 M LiOH as the supporting electrolyte. The scan rate was 20 mVs^{-1}, scanning towards negative potentials first. The experiment was carried out at 25 °C using a 3 mm diameter glassy carbon working electrode, coiled platinum wire reference electrode and a mercury-mercury oxide (1 M NaOH) reference electrode. The first redox peak was isolated by amending the vertex voltages. For clarity, only the first (red) and 400th cycle (blue) are shown.*

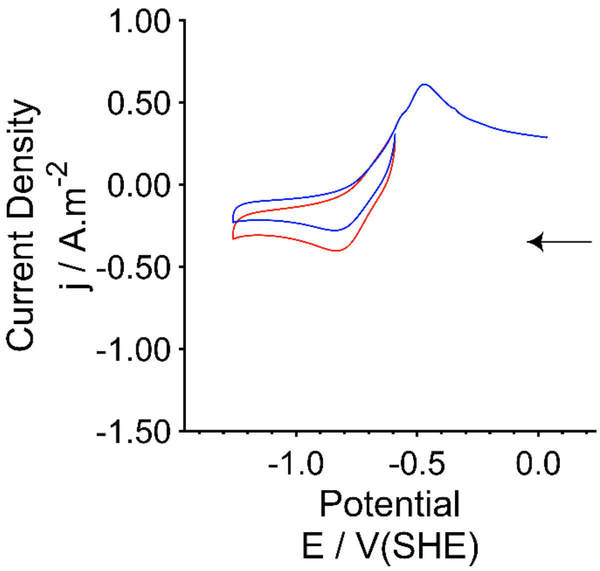

Figure S 7: Cyclic voltammograms of a 1 mM solution of ***2*** *in heavy water (D_2O) with 1 M LiOH as the supporting electrolyte. The scan rate was 20 mVs^{-1}, scanning towards negative potentials first. The experiment was carried out at 25 °C using a 3 mm diameter glassy carbon working electrode, coiled platinum wire reference electrode and a mercury-mercury oxide (1 M NaOH) reference electrode. The second redox peak was isolated by amending the vertex voltages. For clarity, only the first (red) and 400th cycle (blue) are shown.*

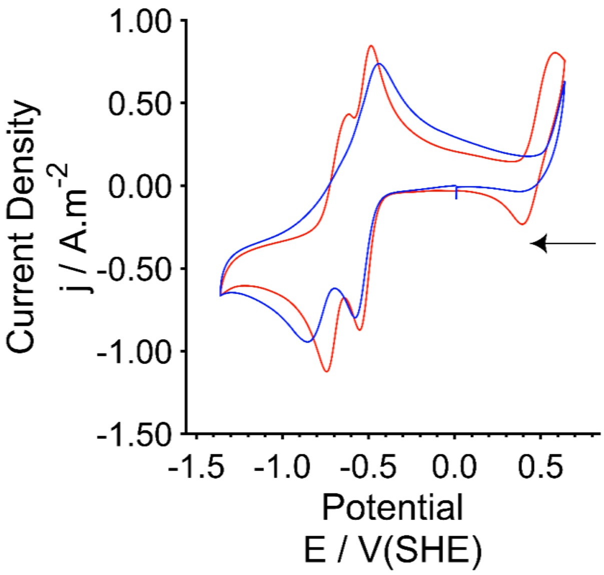

*Figure S 8: Cyclic voltammograms of a 1 mM solution of both **2** and potassium ferrocyanide in heavy water (D_2O) with 1 M LiOH as the supporting electrolyte. The scan rate was 20 mVs^{-1}, scanning towards negative potentials first. The experiment was carried out at 25 °C using a 3 mm diameter glassy carbon working electrode, coiled platinum wire reference electrode and a mercury-mercury oxide (1 M NaOH) reference electrode. For clarity, only the second (red) and 400th cycle (blue) are shown.*

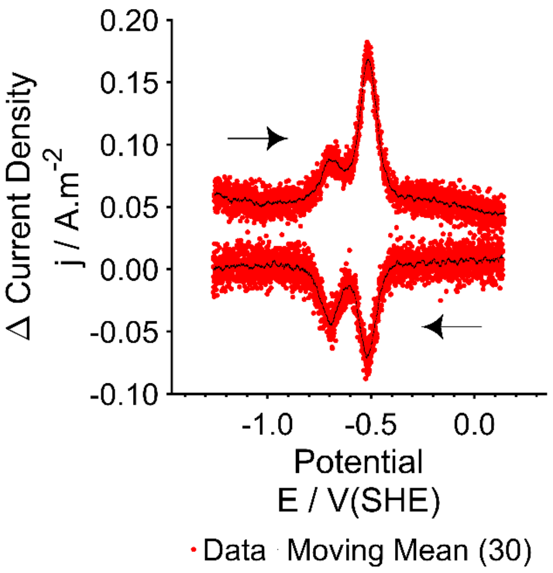

*Figure S 9: Differential Pulse Voltammetry of a 1 mM solution of **2** in heavy water (D_2O) with 1 M LiOH as the supporting electrolyte. The scan rate was 1 mVs^{-1}, scanning towards negative potentials first. The experiment was carried out at 25 °C using a 3 mm diameter glassy carbon working electrode, coiled platinum wire reference electrode and a mercury-mercury oxide (1 M NaOH) reference electrode. A step time of 500 ms was used with a -0.5 mV step height. The pulse width was 100 ms with a pulse height of 2.5 mV. A moving mean across 30 points is added as a guide to the eye.*

4H,8H-benzo[1,2-c:4,5-c']bis([1,2,5]thiadiazole)-4,8-dione (**3**)

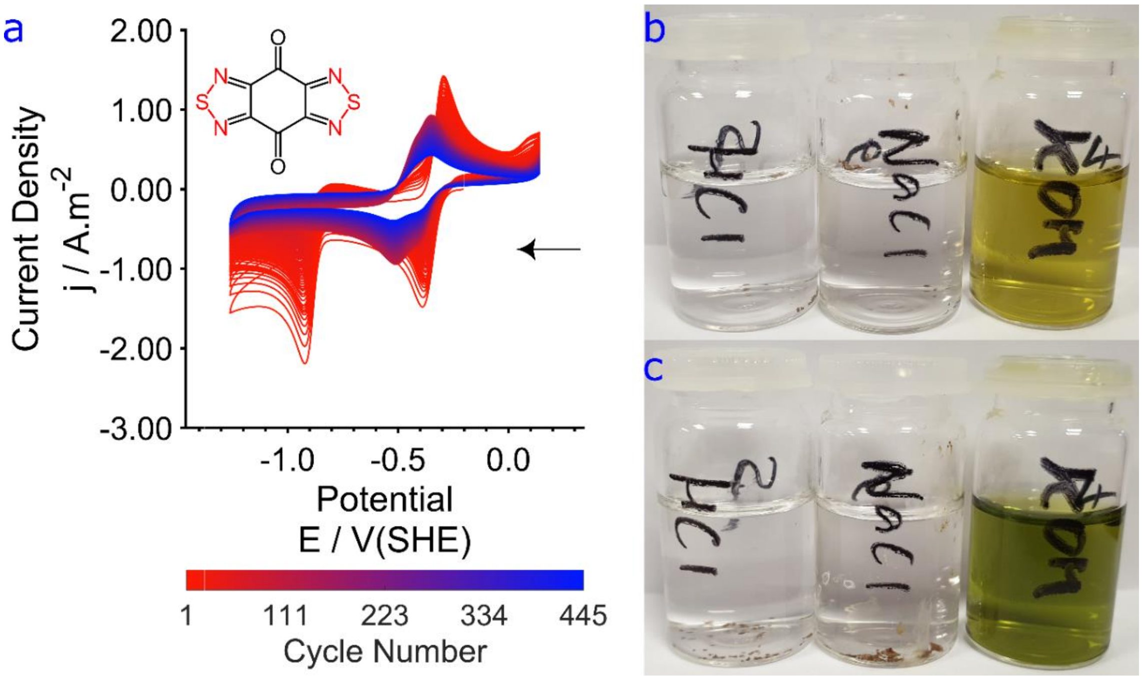

*Figure S 10: (a) Cyclic voltammograms of a 1 mM solution of **3** in D_2O with 1 M potassium hydroxide (KOH) as the supporting electrolyte. The scan rate was 20 mVs^{-1}, scanning towards negative potentials first. The experiment was carried out at 25 °C using a 3 mm diameter glassy carbon working electrode, coiled platinum wire reference electrode and a mercury-mercury oxide (1 M NaOH) reference electrode (b) Image of a sample (freshly prepared) of **3** in 1 M hydrochloric acid (HCl), 1 M sodium chloride (NaCl) and 1 M KOH (c) Image of a sample (after a few minutes) of **3** in 1 M HCl, 1 M NaCl and 1 M KOH.*

2,6-dimethylbenzo[1,2-d:4,5-d']diimidazole-4,8(1H,5H)-dione (**4**)

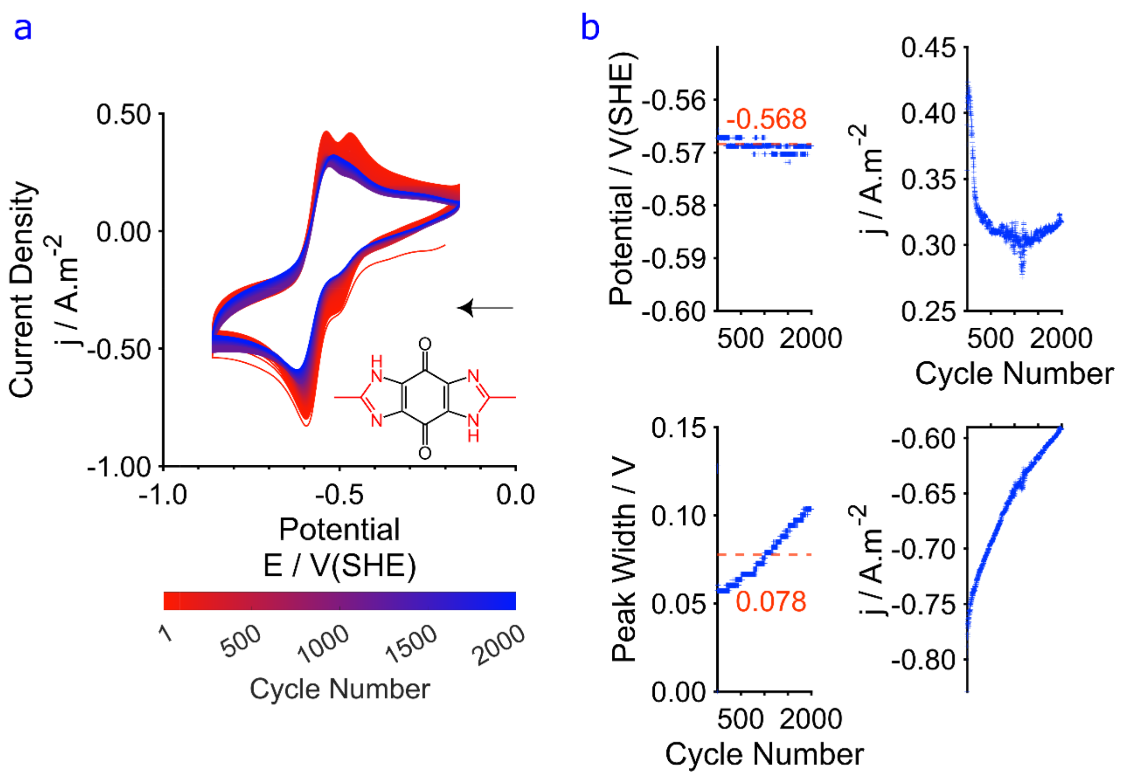

*Figure S 11: (a) Cyclic voltammograms of a 1 mM solution of **4** in D_2O with 1 M KOH as the supporting electrolyte. The scan rate was 20 mVs^{-1}, scanning towards negative potentials first. The experiment was carried out at 25 °C using a 3 mm diameter glassy carbon working electrode, coiled platinum wire reference electrode and a mercury-mercury oxide (1 M NaOH) reference electrode. (b) By tracking the peaks, the average half-wave potential and peak-width were calculated, and the raw currents were extracted.*

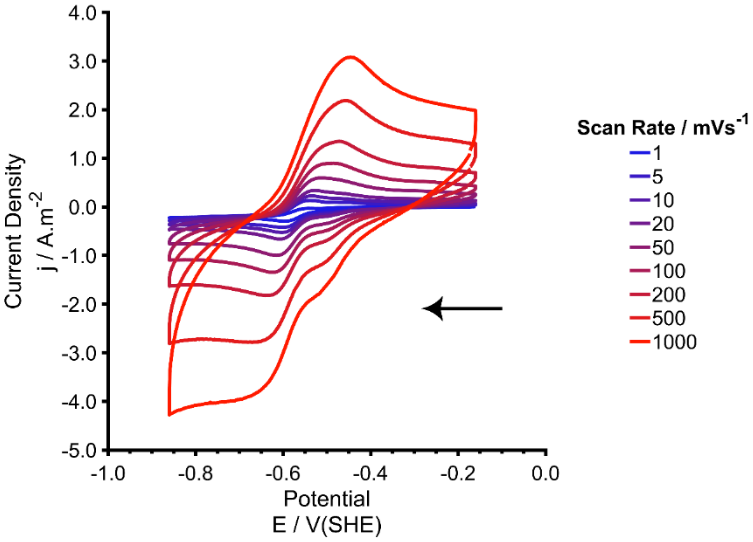

*Figure S 12: Cyclic voltammograms of a 1 mM solution of **4** in D_2O with 1 M KOH as the supporting electrolyte. A range of scan-rates were used, scanning towards negative potentials first. The experiment was carried out at 25 °C using a 3 mm diameter glassy carbon working electrode, coiled platinum wire reference electrode and a mercury-mercury oxide (1 M NaOH) reference electrode.*

Potassium Ferrocyanide

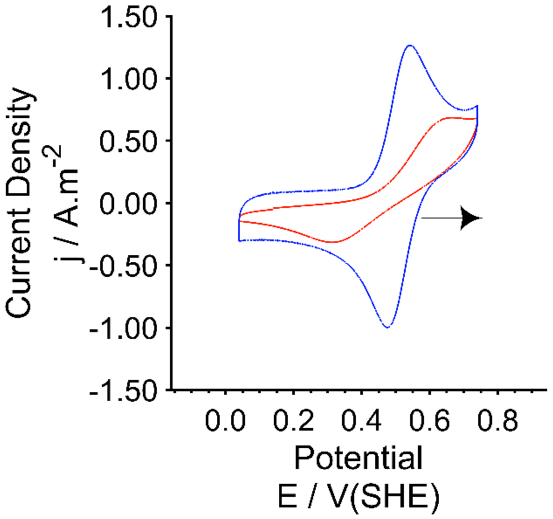

Figure S 13: Cyclic voltammograms of a 1 mM solution of potassium ferrocyanide in heavy water (D_2O) with 1 M LiOH as the supporting electrolyte. The scan rate was 20 mVs^{-1}, scanning towards positive potentials first. The experiment was carried out at 25 °C using a 3 mm diameter glassy carbon working electrode, coiled platinum wire reference electrode and a mercury-mercury oxide (1 M NaOH) reference electrode. For clarity, only the second (red) and 4000th cycle (blue) are shown.

Acidic Media

Neutral Molecules

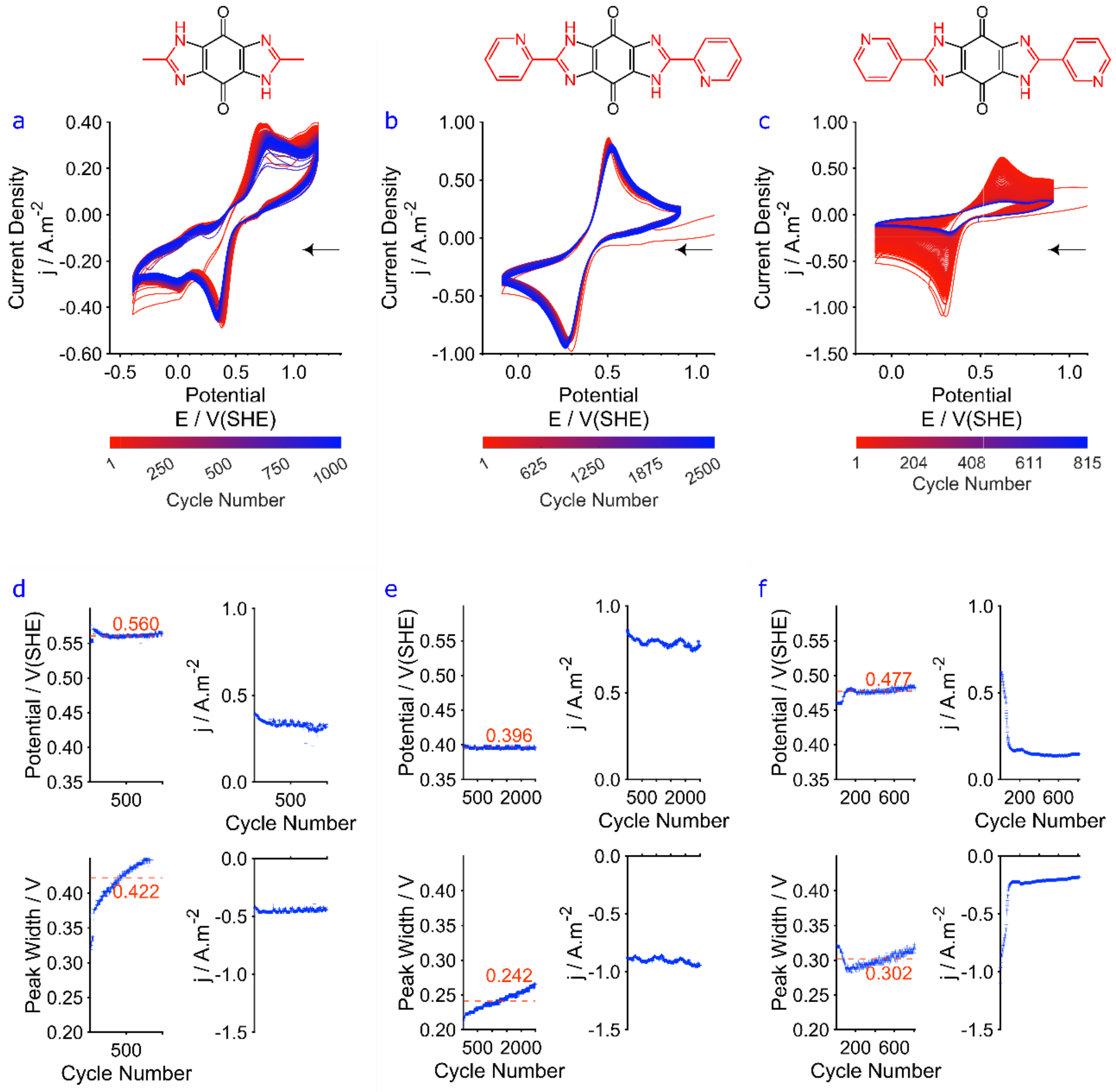

Figure S 14: Cyclic voltammograms of a 1 mM solution of (a) ***4****, (b)* ***5****, (c)* ***6*** *in D_2O with 1 M HCl as the supporting electrolyte. The scan rate was 20 mVs^{-1}, scanning towards negative potentials first. The experiment was carried out at 25 °C using a 3 mm diameter glassy carbon working electrode, coiled platinum wire reference electrode and a silver-silver chloride (3 M NaCl) reference electrode. By tracking the peaks, the average half-wave potential and peak-width were calculated, and the raw currents were extracted for (d)* ***4****, (e)* ***5*** *and (f)* ***6****.*

*Table S 6: Diffusivity of **4** calculated from Randles–Ševčík analyses.*

**Species**	**Diffusivity (Randles–Ševčík) / 10^{-7} cm^2s^{-1} (R^2)**
	Red
**4**	2 (0.9980)

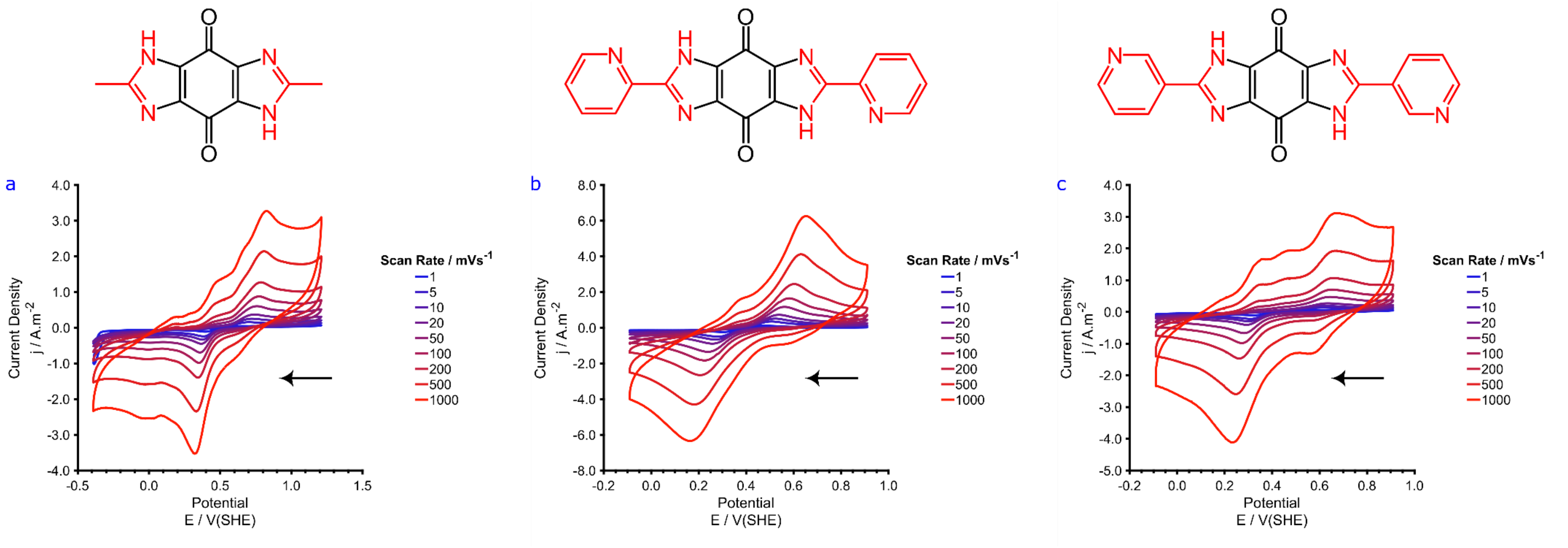

Figure S 15: Cyclic voltammograms of a 1 mM solution of (a) ***4****, (b)* ***5****, (c)* ***6*** *in heavy water (D_2O) with 1 M hydrochloric acid (HCl) as the supporting electrolyte. A range of scan-rates were used, scanning towards negative potentials first. The experiment was carried out at 25 °C using a 3 mm diameter glassy carbon working electrode, coiled platinum wire reference electrode and a silver-silver chloride (3 M NaCl) reference electrode.*

Pyridinium Salts

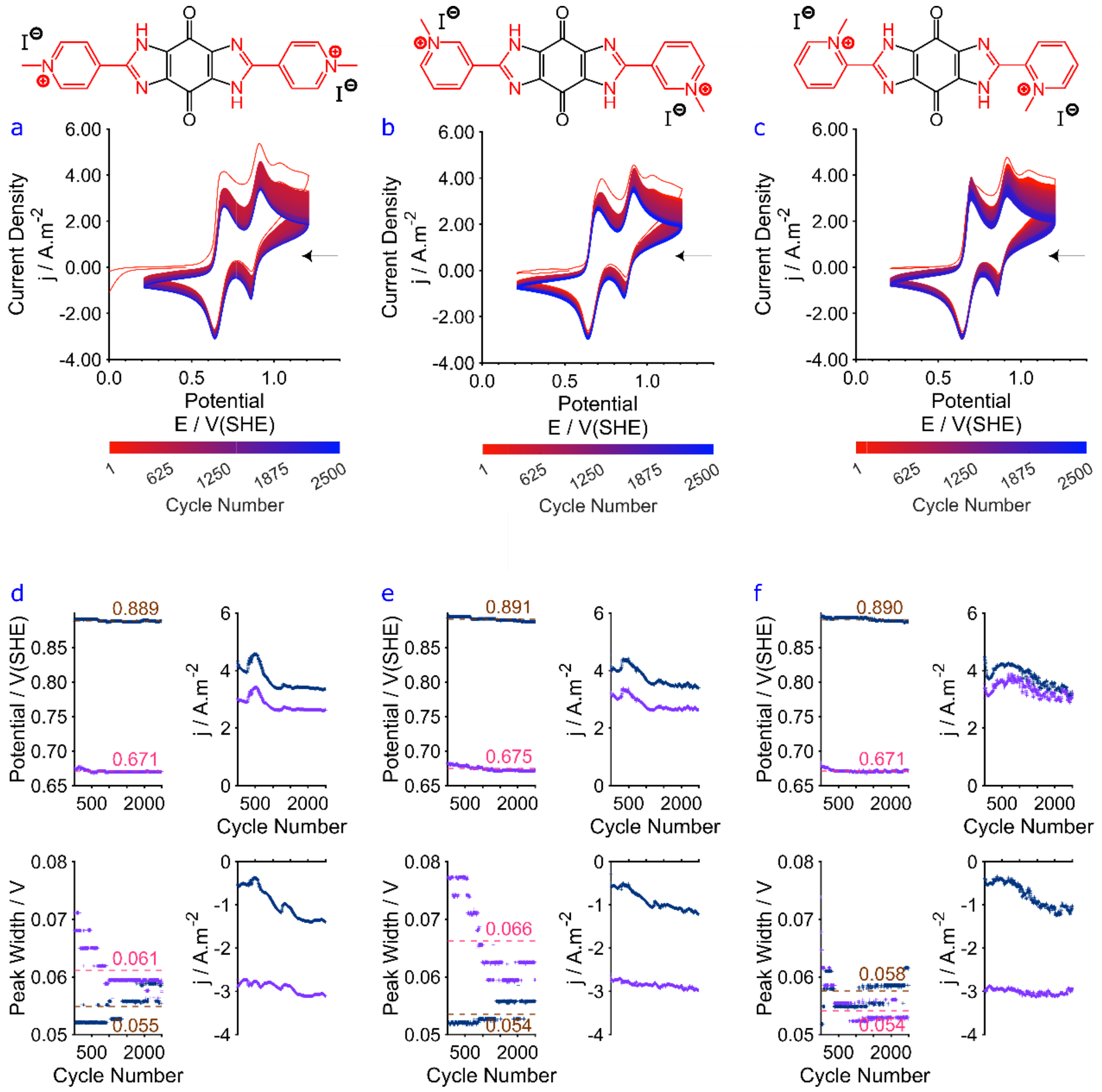

*Figure S 16: Cyclic voltammograms of a 1 mM solution of (a) **8**, (b) **9**, (c) **7** in heavy water (D_2O) with 1 M hydrochloric acid (HCl) as the supporting electrolyte. The scan rate was 20 mVs^{-1}, scanning towards negative potentials first. The experiment was carried out at 25 °C using a 3 mm diameter glassy carbon working electrode, coiled platinum wire reference electrode and a silver-silver chloride (3 M NaCl) reference electrode. By tracking the peaks, the average half-wave potential and peak-width were calculated, and the raw currents were extracted for (d) **8**, (e) **9** and (f) **7**.*

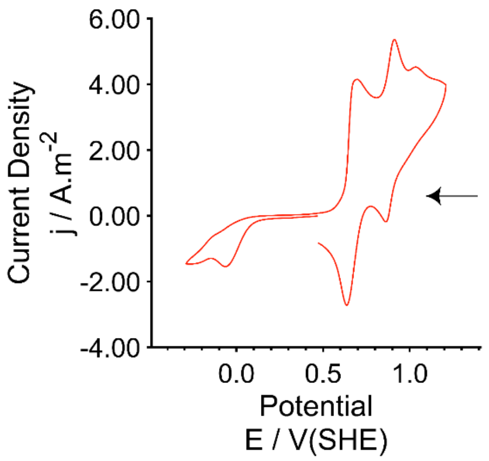

*Figure S 17: Cyclic voltammogram of a 1 mM solution of **8** in heavy water (D_2O) with 1 M hydrochloric acid (HCl) as the supporting electrolyte. The scan rate was 20 mVs^{-1}, scanning towards negative potentials first. The experiment was carried out at 25 °C using a 3 mm diameter glassy carbon working electrode, coiled platinum wire reference electrode and a silver-silver chloride (3 M NaCl) reference electrode.*

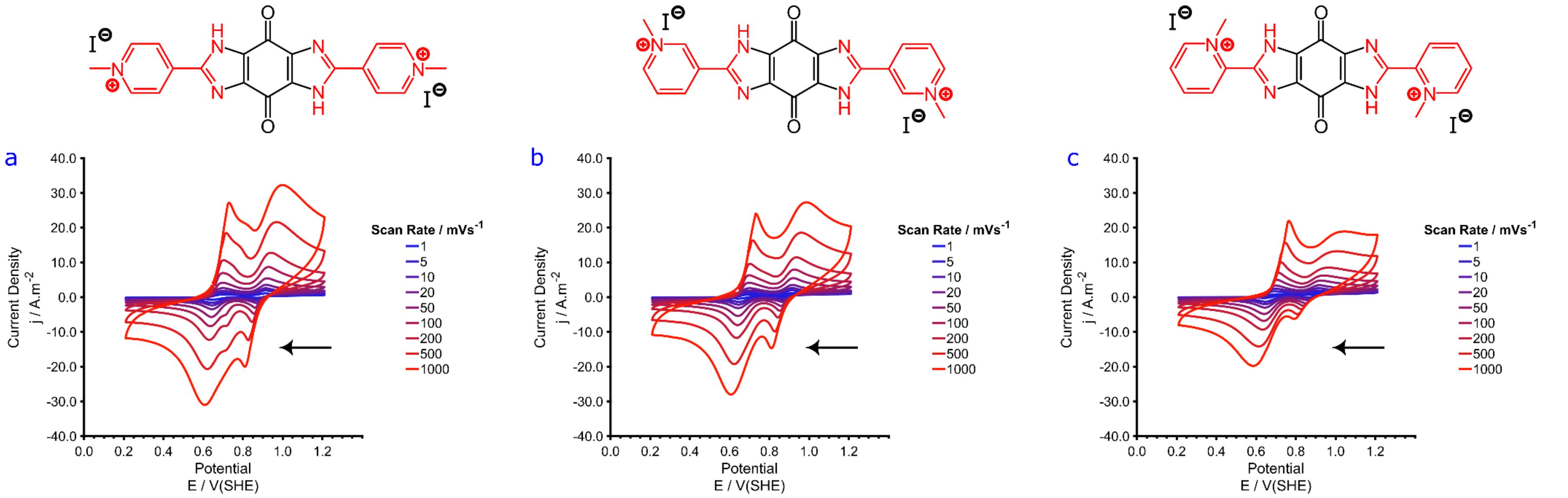

Figure S 18: Cyclic voltammograms of a 1 mM solution of (a) ***8****, (b)* ***9****, (c)* ***7*** *in heavy water (D_2O) with 1 M hydrochloric acid (HCl) as the supporting electrolyte. A range of scan-rates were used, scanning towards negative potentials first. The experiment was carried out at 25 °C using a 3 mm diameter glassy carbon working electrode, coiled platinum wire reference electrode and a silver-silver chloride (3 M NaCl) reference electrode.*

*Table S 7: Diffusivities of **8**, **9** and **7** calculated from the Randles–Ševčík. The | for the Randles–Ševčík results denotes the different values calculated for the high (left) or low (right) potential redox events.*

Species	Diffusivity (Randles–Ševčík) / 10^{-6} cm^2s^{-1} (R^2)
**8**	4 (0.9788)
**9**	5 (0.9964)
**7**	9 (0.9977)

Neutral Media

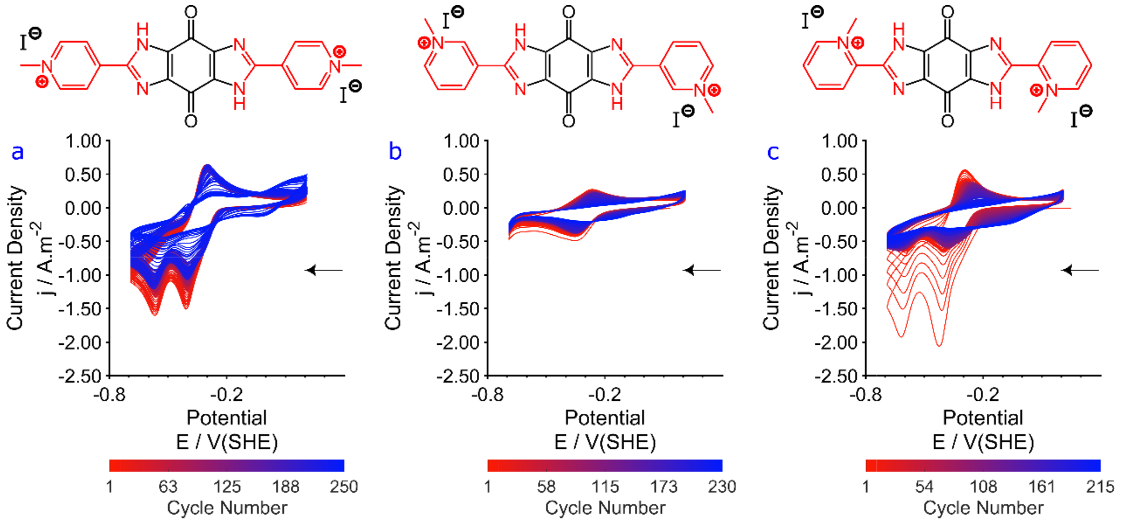

*Figure S 19: Cyclic voltammograms of a 1 mM solution of (a) **8**, (b) **9**, (c) **7** in heavy water (D_2O) with 1 M sodium chloride (NaCl) as the supporting electrolyte. The scan rate was 20 mVs^{-1}, scanning towards negative potentials first. The experiment was carried out at 25 °C using a 3 mm diameter glassy carbon working electrode, coiled platinum wire reference electrode and a silver-silver chloride (3 M NaCl) reference electrode.*

Non-aqueous Media

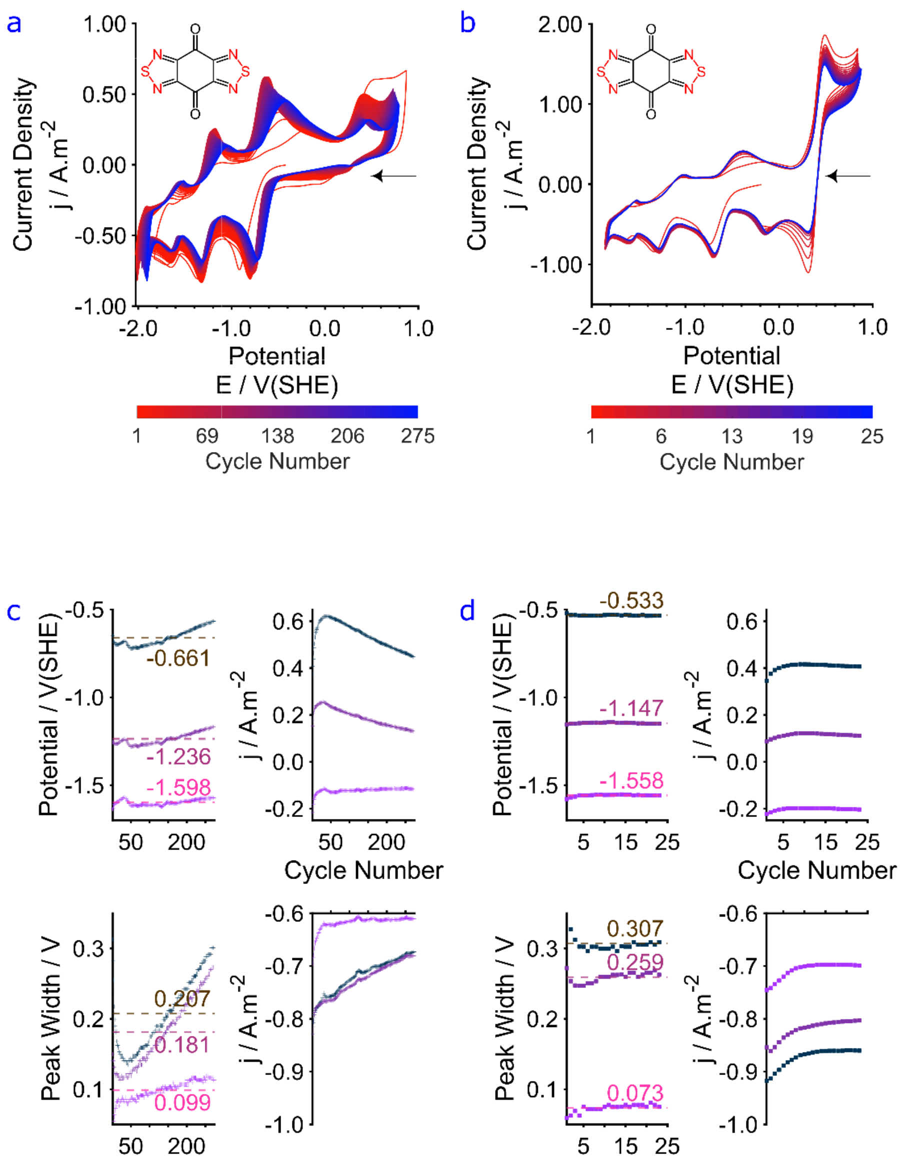

*Figure S 20: Cyclic voltammograms of a 1 mM solution of **3** in dry, degassed DMSO with 100 mM tetra-n-butyl ammonium hexafluorophosphate (TBAPF$_6$) as the supporting electrolyte. The scan rate was 20 mVs^{-1}, scanning towards negative potentials first at room temperature within a nitrogen-filled glovebox. A 3 mm diameter glassy carbon working electrode, coiled platinum wire and silver wire pseudo-reference was used. The internal standard was ferrocene. (a) shows the initial behaviour with a low concentration of ferrocene present in the solution while (b) shows the behaviour with additional ferrocene having been added to the solution (c, d) By tracking the peaks, the average half-wave potential and peak-width were calculated, and the raw currents were extracted.*

Asymmetric Cells

Run 1

Specific area resistance (two Nafion 212 membranes) = 1.595 ± 0.016 Ωcm^2 (charge: 1.497 ± 0.014 Ωcm^2, discharge: 1.69 ± 0.03 Ωcm^2)

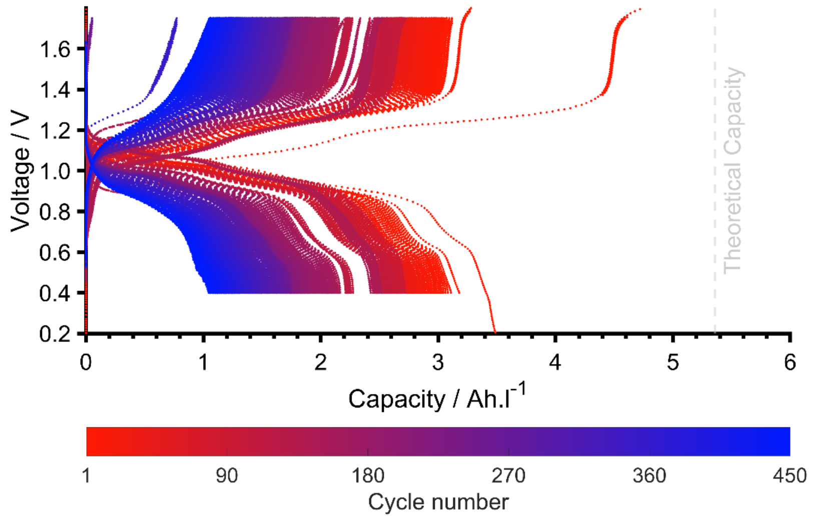

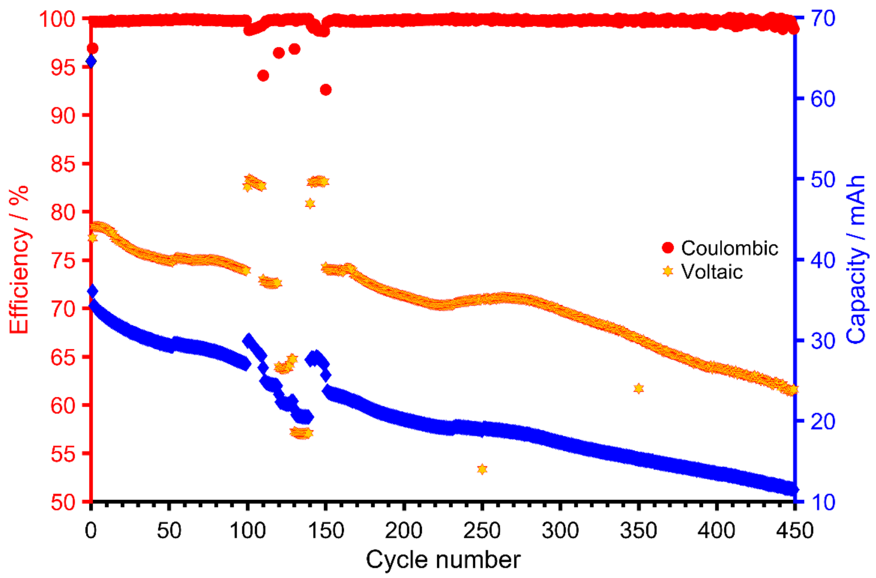

*Figure S 21: Lab-scale battery performance of **2** against potassium ferrocyanide in D_2O with 1 M LiOH added as the supporting electrolyte at ambient temperature under an inert nitrogen atmosphere. The battery was operated between 0.40 and 1.75 V at a current of 200 mA. Limiting tank: 100 mM **2**, 11.0 ml; non-limiting tank:150 mM $K_4Fe(CN)_6.3H_2O$, 37.5 mM $K_3Fe(CN)_6$, 37.8 ml. Two layers Nafion 212 pre-soaked in 0.1 M LiOH. Flow rate = 80 rpm (50 ml.min^{-1}). Carbon paper (3x per side, Sigracet GDL-39AA) pre-baked at 400 °C for 24 hrs was used as the electrode. Cycles 101-150 were carried out under different charging currents.*

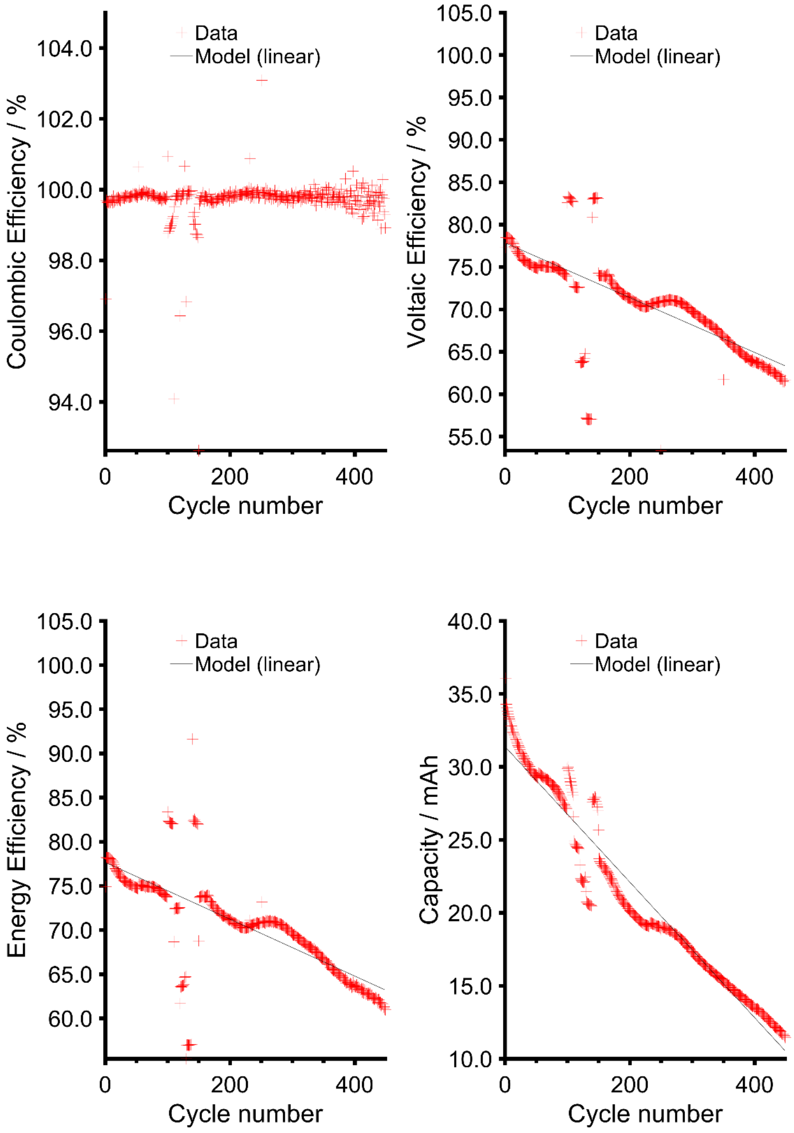

Figure S 22: (Top left) Coulombic efficiency plotted against cycle number with a linear regression line, (Top right) Voltaic efficiency plotted against cycle number with a linear regression line, (Bottom left) energy efficiency plotted against cycle number with a linear regression line, (Bottom right) capacity plotted against cycle number with a linear regression line. Cycles 100-150 were carried out under different charging currents.

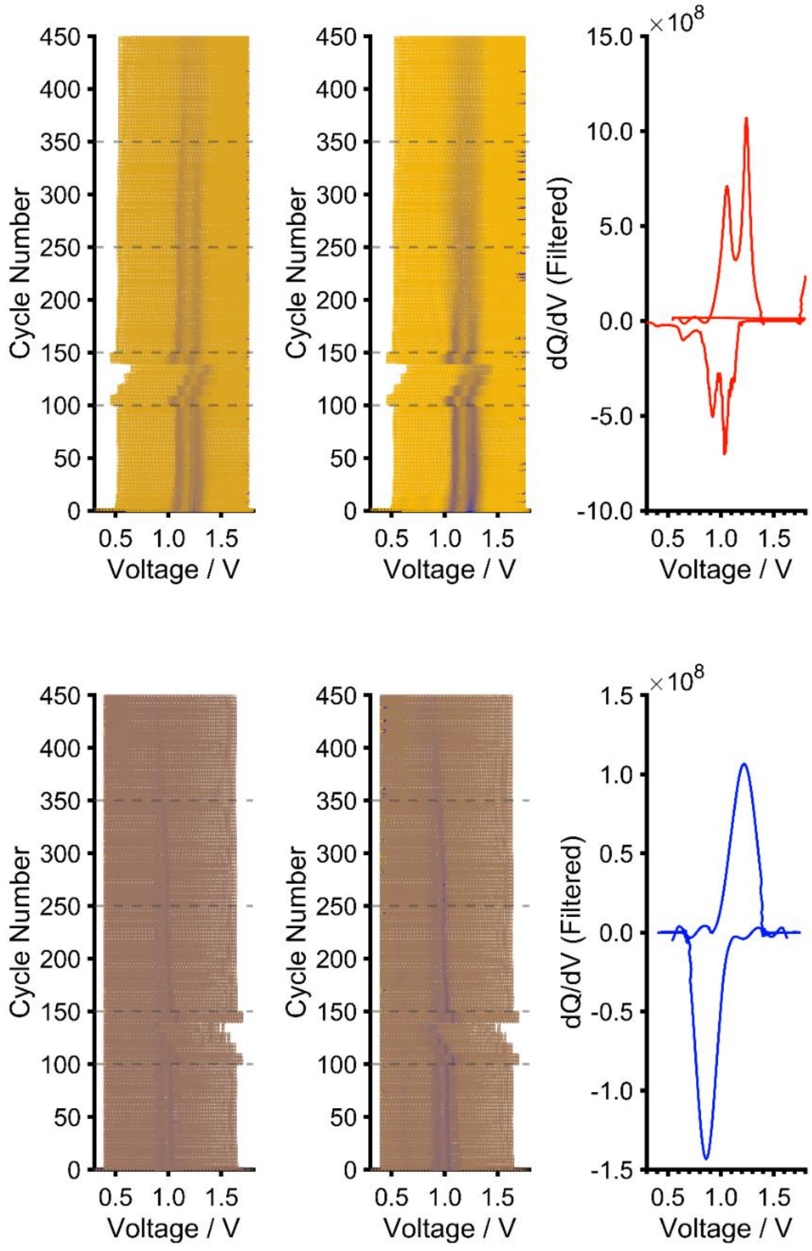

*Figure S 23: dQ/dV data corresponding to the battery performance of **2** against potassium ferrocyanide in D_2O with 1 M LiOH added as the supporting electrolyte at ambient temperature under an inert nitrogen atmosphere. Limiting tank: 100 mM **2**, 11.0 ml; non-limiting tank:150 mM $K_4Fe(CN)_6.3H_2O$, 37.5 mM $K_3Fe(CN)_6$, 37.8 ml. Two layers Nafion 212 pre-soaked in 0.1 M LiOH. Flow rate = 80 rpm (50 $ml.min^{-1}$). Carbon paper (3x per side, Sigracet GDL-39AA) pre-baked at 400 °C for 24 hrs was used as the electrode. The left graphs reflect the numerically calculated values from the raw data, the middle graphs show the data smoothed with a Savitzky-Golay filter (top: charge, bottom: discharge). Top-right shows the dQ/dV from the first cycle while the bottom-right shows the dQ/dV from the last cycle. Cycles 101–150 were carried out under different charging currents.*

Table S 8: Coulombic and Voltaic efficiencies and capacity fade rates for the full-cell (crude) system over the range of current densities tested in Figure S 24 over 10 cycles.

Current Density / $mA.cm^{-2}$	Coulombic Efficiency / %	Voltaic Efficiency / %	Capacity fade / $mAh.cycle^{-1}$ (R^2)
**20**	99.00 ± 0.06	82.99 ± 0.09	0.243 (0.997)
**40**	99.78 ± 0.04	72.62 ±0.02	0.0524 (0.919)
**60**	99.9 ± 0.1	64.1 ± 0.2	-0.00829 (0.020)
**80**	99.90 ± 0.03	57.06 ± 0.02	0.0247 (0.893)
**20**	98.91 ± 0.09	83.13 ± 0.02	0.0874 (0.607)

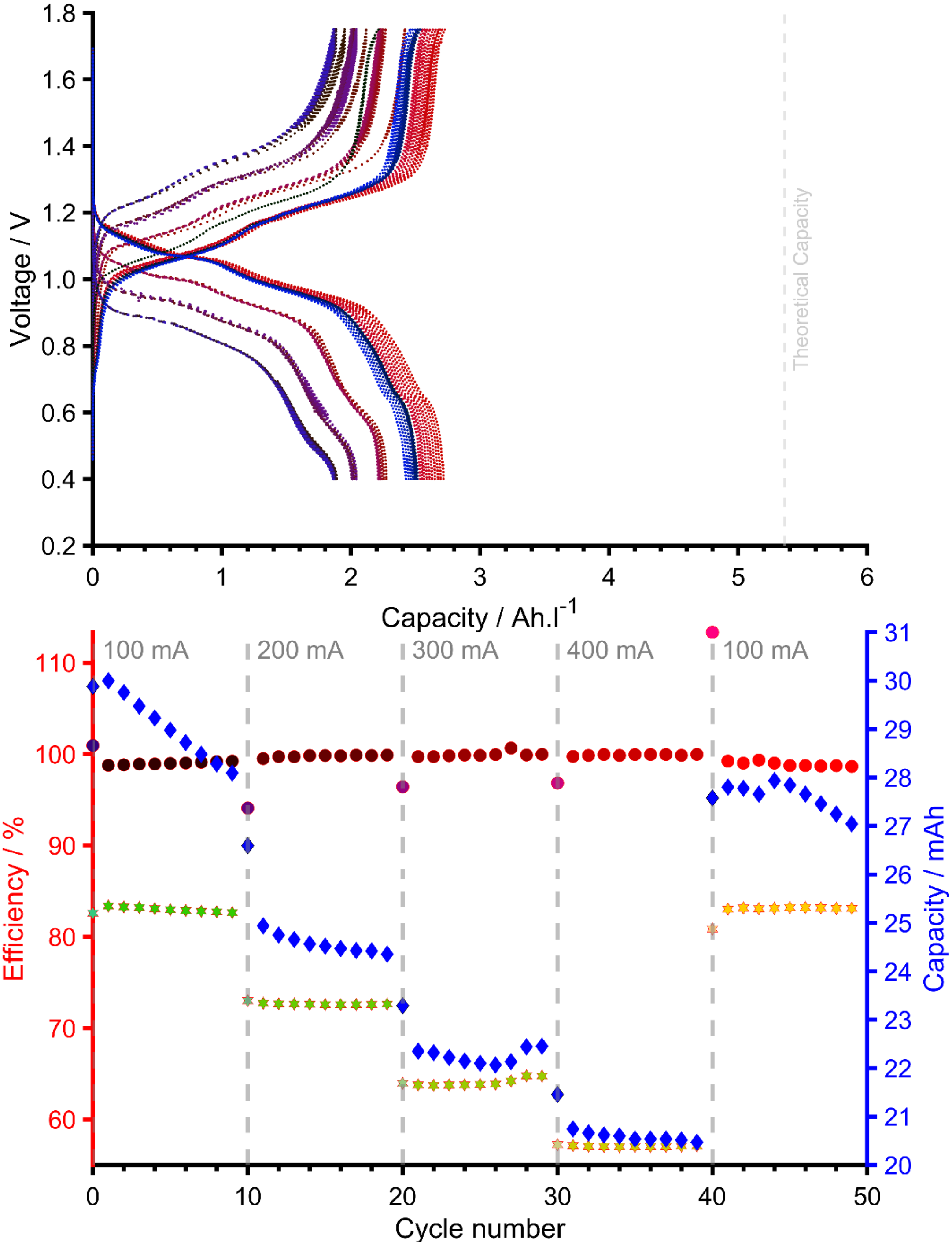

Figure S 24: Battery performance of ***2*** *against potassium ferrocyanide in D_2O with 1 M LiOH added as the supporting electrolyte at ambient temperature under an inert nitrogen atmosphere under different charging current densities. Limiting tank: 100 mM* ***2****, 11.0 ml; non-limiting tank:150 mM $K_4Fe(CN)_6.3H_2O$, 37.5 mM $K_3Fe(CN)_6$, 37.8 ml. Two layers Nafion 212 pre-soaked in 0.1 M LiOH. Flow rate = 80 rpm (50 ml.min^{-1}). Carbon paper (3x per side, Sigracet GDL-39AA) pre-baked at 400 °C for 24 hrs was used as the electrode. 10 cycles were run at 20, 40, 60, 80 and 20 mA.cm^{-2} after 100 cycles at 40 mA.cm^{-2}. The red hued circles represent Coulombic efficiency while the green-yellow stars represent Voltaic efficiency.*

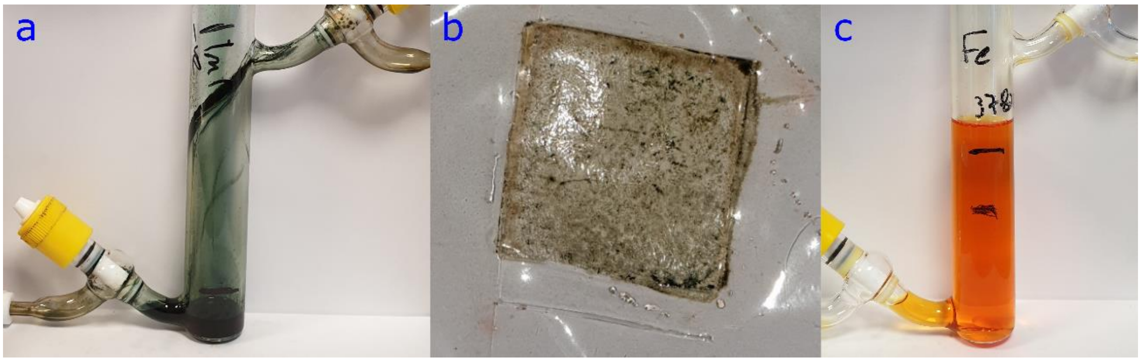

Figure S 25: Photographs of the (a) anolyte tank, (b) Nafion 212 membranes and (c) catholyte tank after cycling for the crude material full-cell test. The black marker lines denote the volumes in the tanks during battery operation (lower line) and tank isolation (upper line) and act as a qualitative guide to volume change.

Run 2

Specific area resistance (two Nafion 212 membranes) = 1.0622 ± 0.013 Ωcm^2 (charge: 1.0122 ± 0.011 Ωcm^2, discharge: 1.112 ± 0.03 Ωcm^2).

Table S 9: Coulombic and Voltaic efficiencies and capacity fade rates for the full-cell (recrystallised) system over the range of current densities tested in Figure S 27 over 10 cycles.

Current Density / mA.cm^{-2}	Coulombic Efficiency / %	Voltaic Efficiency / %	Capacity fade / mAh.cycle^{-1} (R^2)
**10**	94 ± 2	87 ± 2	1.0448 (0.7937)
**20**	99.35 ± 0.02	87.466 ± 0.004	0.0134 (0.9791)
**40**	99.65 ± 0.04	79.35 ± 0.02	0.0044 (0.6871)
**60**	99.758 ± 0.006	72.21 ± 0.04	0.0039 (0.7913)
**80**	99.83 ± 0.03	66.12 ± 0.05	0.0027 (0.5123)
**20**	99.44 ± 0.03	87.93 ± 0.03	-0.0096 (0.7725)

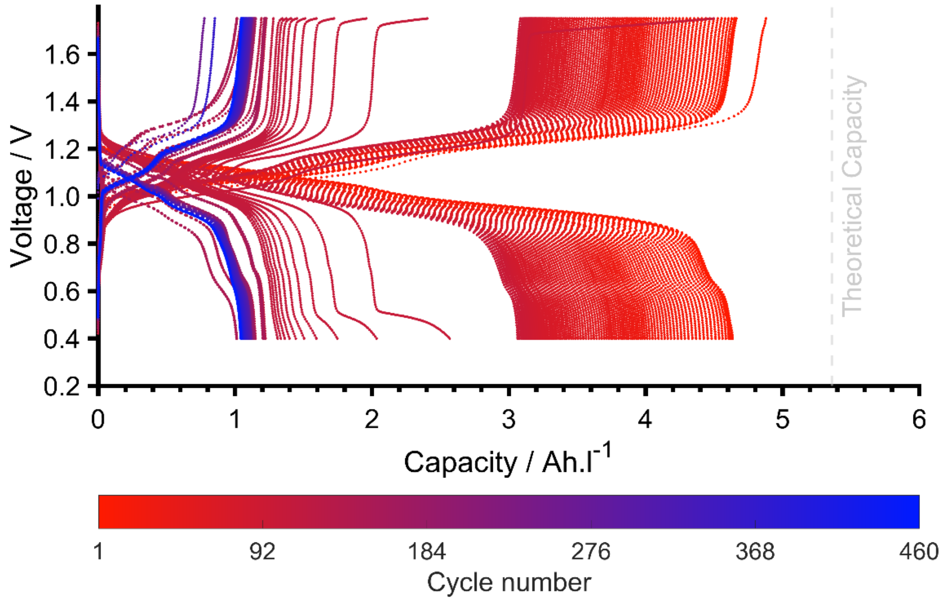

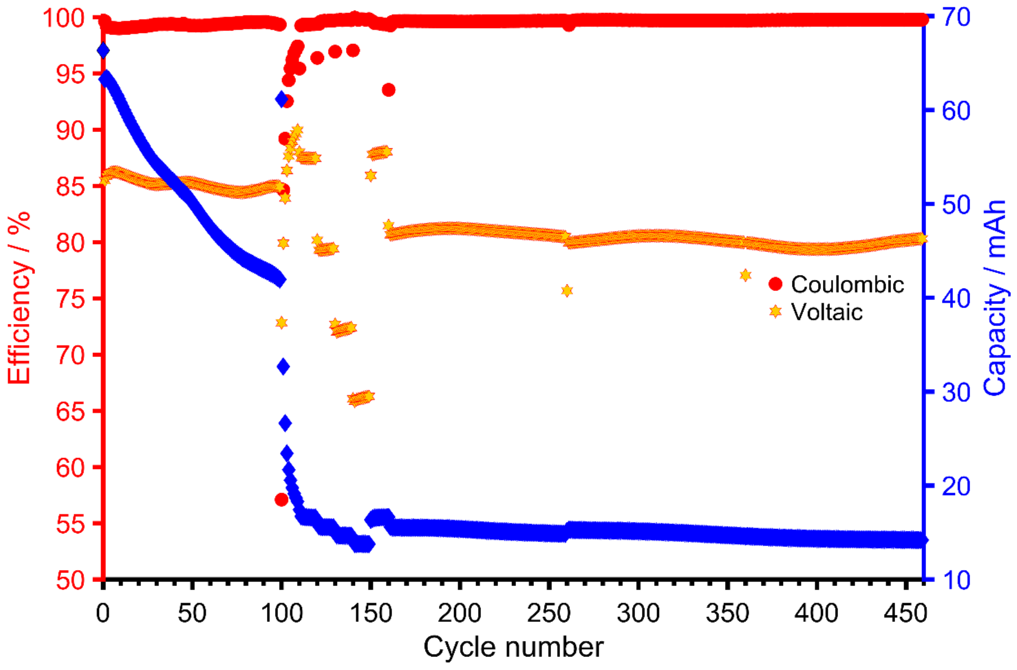

Figure S 26: Lab-scale battery performance of ***2*** *against potassium ferrocyanide in D_2O with 1 M LiOH added as the supporting electrolyte at ambient temperature under an inert nitrogen atmosphere. The battery was operated between 0.40 and 1.75 V at a current of 200 mA. Limiting tank: 100 mM* ***2****, 13.6 ml; non-limiting tank:300 mM $K_4Fe(CN)_6.3H_2O$, 75 mM $K_3Fe(CN)_6$, 20.1 ml. Two layers Nafion 212 pre-soaked in 0.1 M LiOH. Flow rate = 80 rpm (50 ml.min^{-1}). Carbon paper (3x per side, Sigracet GDL-39AA) pre-baked at 400 °C for 24 hrs was used as the electrode. Cycles 101–160 were gathered under different charging currents.*

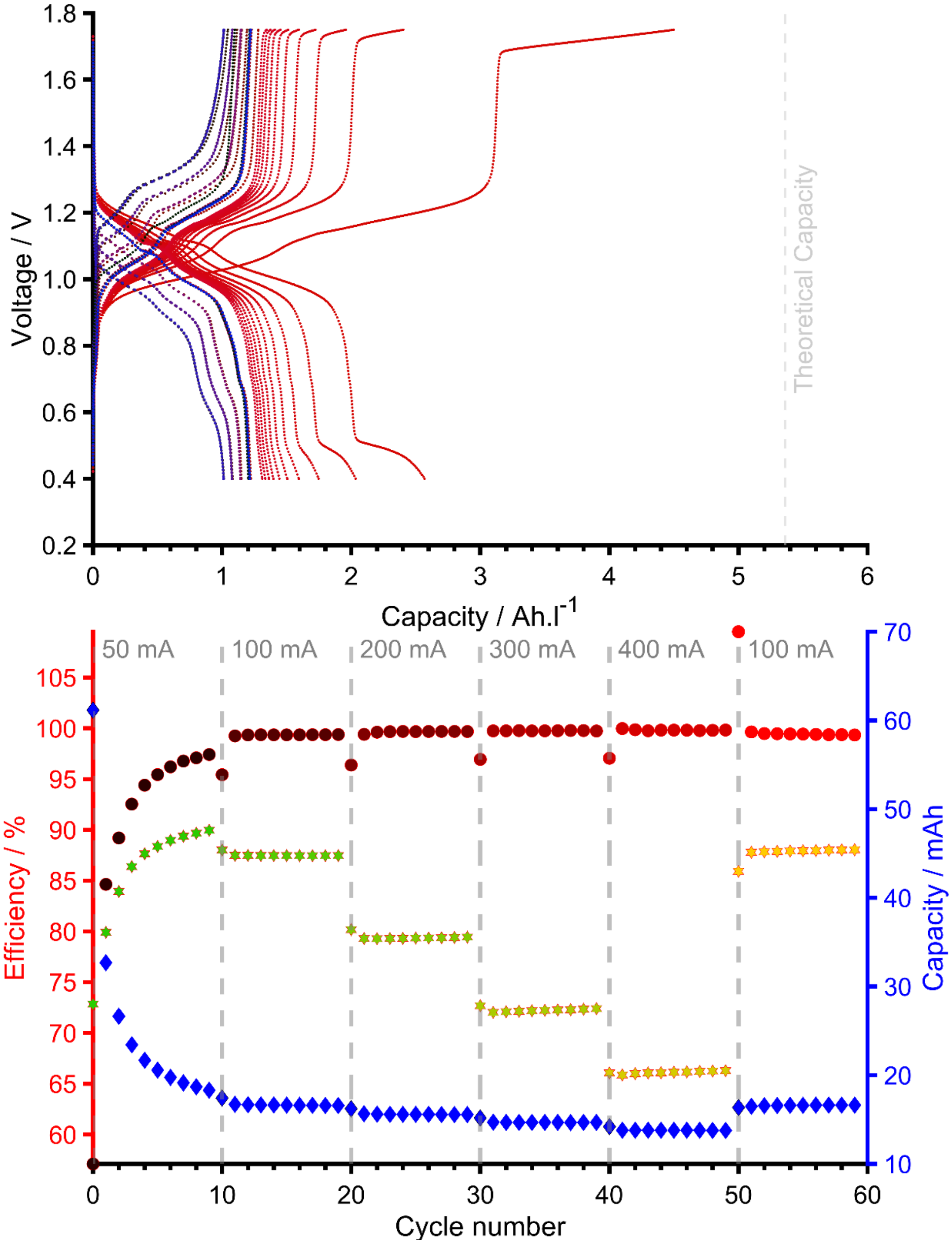

*Figure S 27: Battery performance of **2** against potassium ferrocyanide in D_2O with 1 M LiOH added as the supporting electrolyte at ambient temperature under an inert nitrogen atmosphere under different charging currents. Limiting tank: 100 mM **2**, 13.6 ml; non-limiting tank:300 mM $K_4Fe(CN)_6.3H_2O$, 75 mM $K_3Fe(CN)_6$, 20.1 ml. Two layers Nafion 212 pre-soaked in 0.1 M LiOH. Flow rate = 80 rpm (50 ml.min^{-1}). Carbon paper (3x per side, Sigracet GDL-39AA) pre-baked at 400 °C for 24 hrs was used as the electrode. 10 cycles were run at 10, 20, 40, 60, 80 and 20 mA.cm^{-2} after 100 cycles at 40 mA.cm^{-2}. The red hued circles represent Coulombic efficiency while the green-yellow stars represent Voltaic efficiency.*

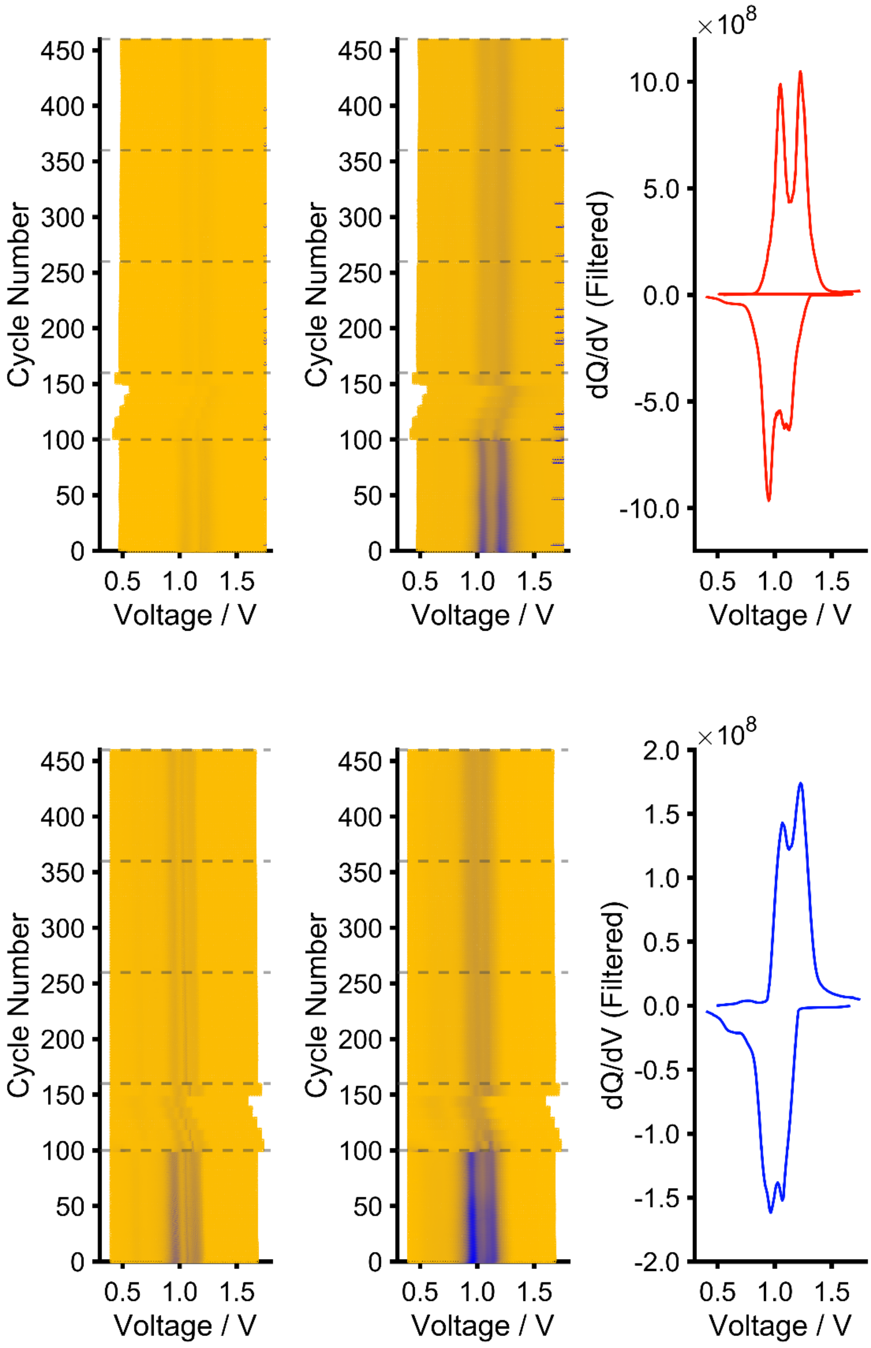

Figure S 28: dQ/dV data corresponding to the battery performance of ***2*** *against potassium ferrocyanide in D_2O with 1 M LiOH added as the supporting electrolyte at ambient temperature under an inert nitrogen atmosphere. Limiting tank: 100 mM* ***2****, 13.6 ml; non-limiting tank:300 mM $K_4Fe(CN)_6.3H_2O$, 75 mM $K_3Fe(CN)_6$, 20.1 ml. Two layers Nafion 212 pre-soaked in 0.1 M LiOH. Flow rate = 80 rpm (50 ml.min^{-1}). Carbon paper (3x per side, Sigracet GDL-39AA) pre-baked at 400 °C for 24 hrs was used as the electrode. The left graphs reflect the numerically calculated values from the raw data, the middle graphs show the data smoothed with a Savitzky-Golay filter (top: charge, bottom: discharge). Top-right shows the dQ/dV from the first cycle while the bottom-right shows the dQ/dV from the last cycle. Cycles 101–160 were gathered under different charging currents.*

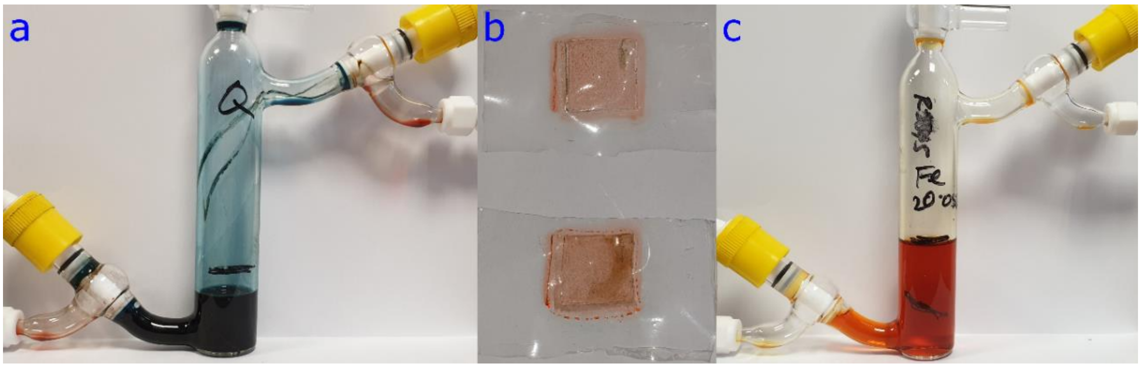

Figure S 29: Photographs of the (a) anolyte tank, (b) Nafion 212 membranes and (c) catholyte tank after cycling for the crude material full-cell test. The black marker lines denote the volumes in the tanks during battery operation (lower line) and tank isolation (upper line) and act as a qualitative guide to volume change.

Experimental Parameters

- The battery was operated between 0.40 and 1.75 V at a current of 200 mA (40 mA.cm^{-2}).
- Limiting tank: 100 mM **2**, 13.6 ml
- Non-limiting tank: 300 mM $K_4Fe(CN)_6.3H_2O$, 75 mM $K_3Fe(CN)_6$, 20.1 ml.
- Two layers Nafion 212 pre-soaked in 0.1 M LiOH.
- Flow rate = 80 rpm (50 ml.min^{-1}).
- Carbon paper (3x per side, Sigracet GDL-39AA) pre-baked at 400 °C for 24 hrs was used as the electrode.

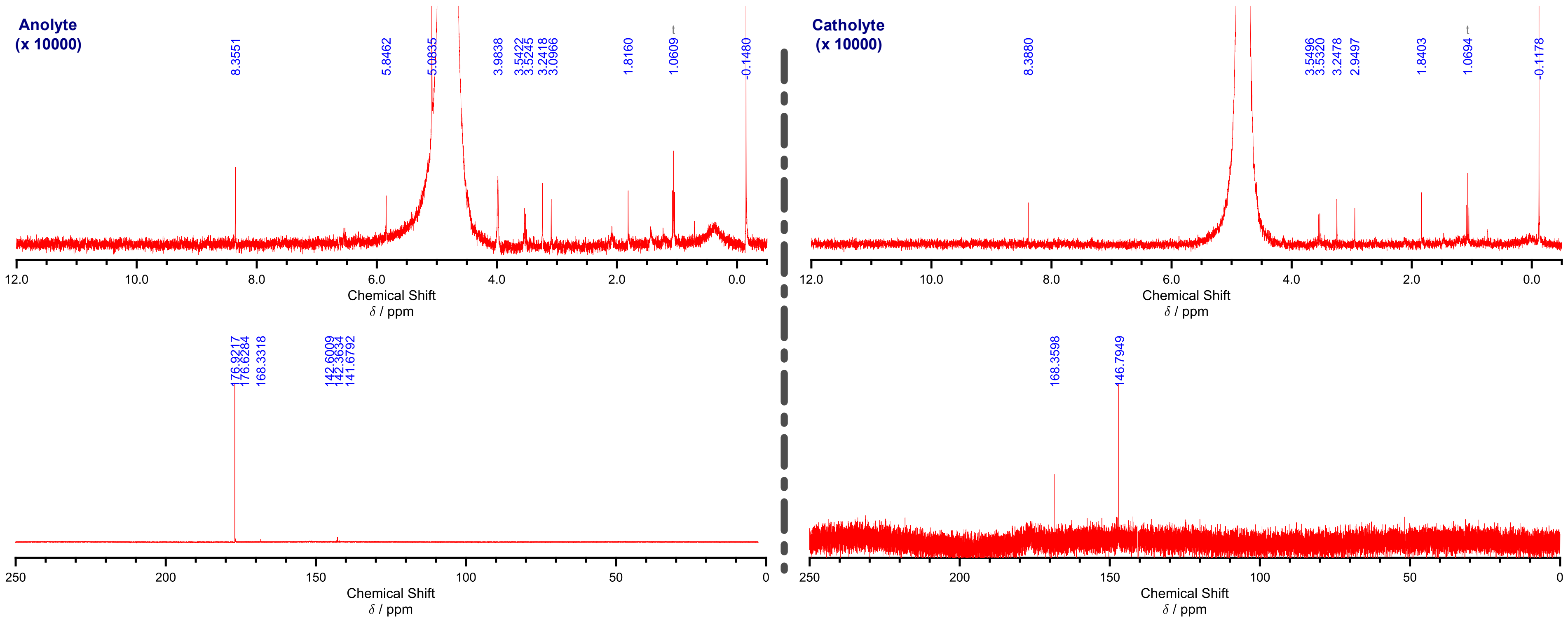

*Figure S 30: ^{1}H (above) and ^{13}C NMR (below) of the anolyte tank containing **2** (left) and the catholyte tank (right) containing hexacyanoferrate after battery cycling. The battery cycling data is presented in Figure 4 of the main matter. The peak at 4.79 ppm in the ^{1}H NMR corresponds to the HOD peak in D_2O while the peak below 0 ppm can be attributed to silicon grease. The intensity of the ^{1}H spectrum was enhanced 10 000-fold. The carbon spectra are presented without any additional scaling.*

Ultraviolet-Visible Spectroscopy

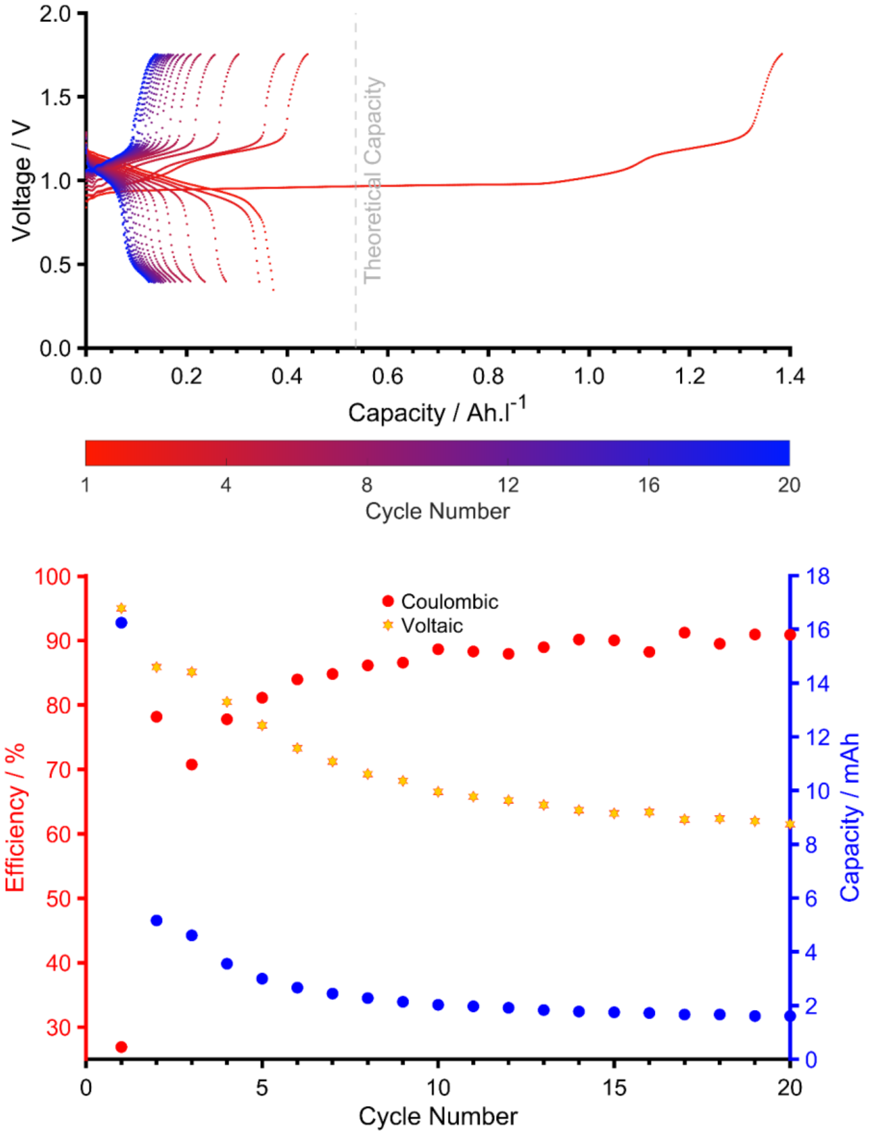

Figure S 31: Galvanostatic cycling data corresponding to the in-situ UV-Vis experiment. The lab-scale battery of ***2*** *against potassium ferrocyanide in* D_2O *with 1 M LiOH added as the supporting electrolyte was run at ambient temperature under an inert nitrogen atmosphere.*

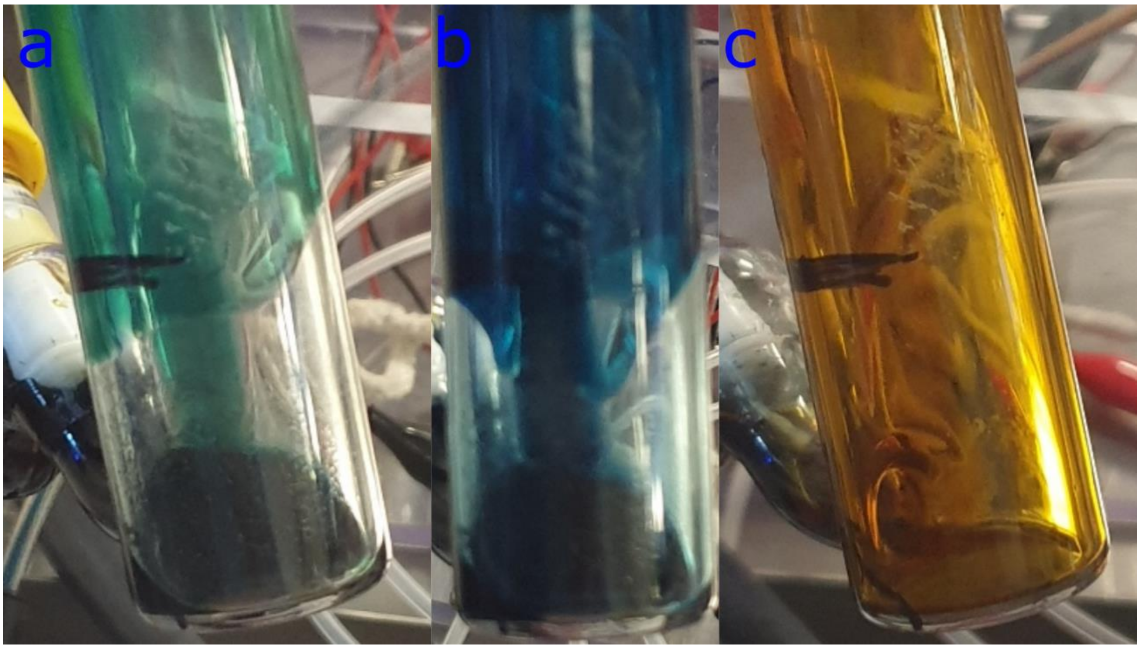

Figure S 32: Photographs of the anolyte tank at (a) full discharge, (b) half-charge and (c) full charge.

Simulated spectra were generated using the formulae described in reference [31]

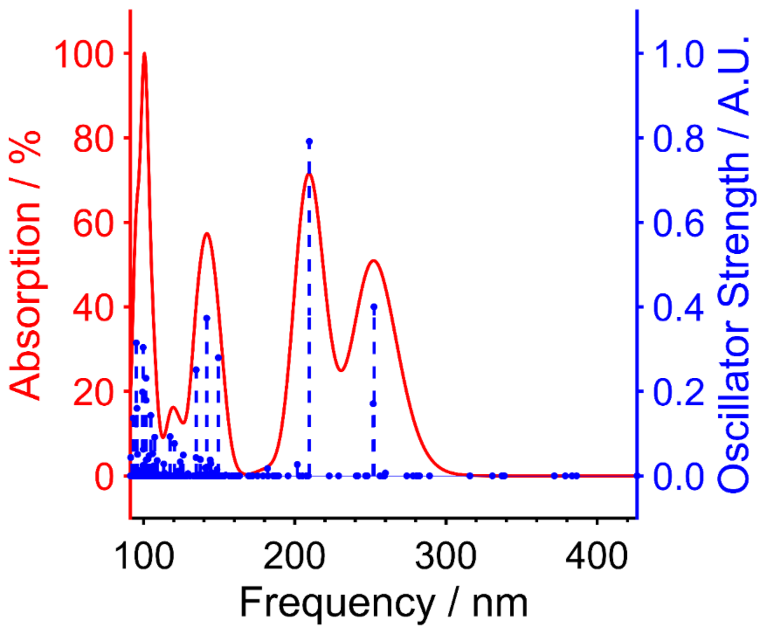

Figure S 33: Calculated spectrum for ***2***$^{2-}$ *at the CAM-B3LYP/TZVP level of theory (TD-DFT, 50:50 singlet: triplet, no. states = 200).*

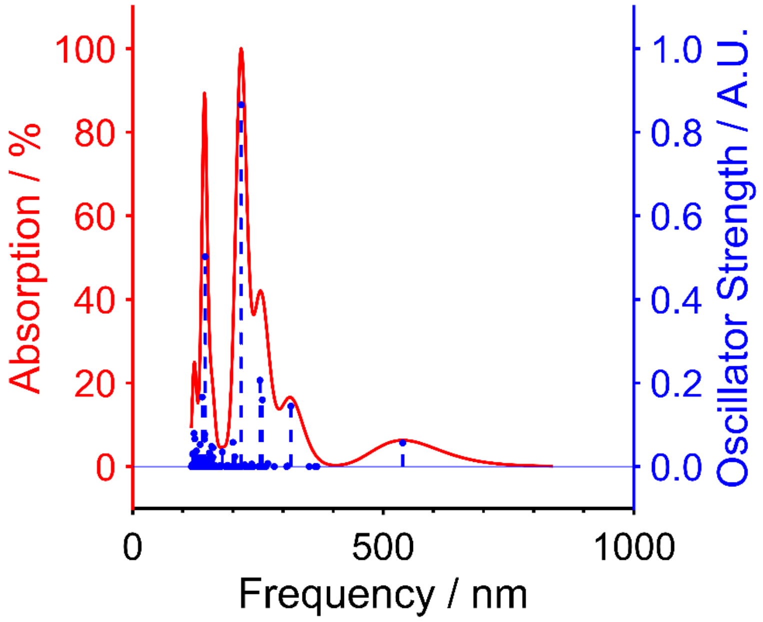

Figure S 34: Calculated spectrum for ***2***$^{3-\cdot}$ *at the CAM-B3LYP/TZVP level of theory (TD-DFT, 50:50 singlet: triplet, no. states = 200).*

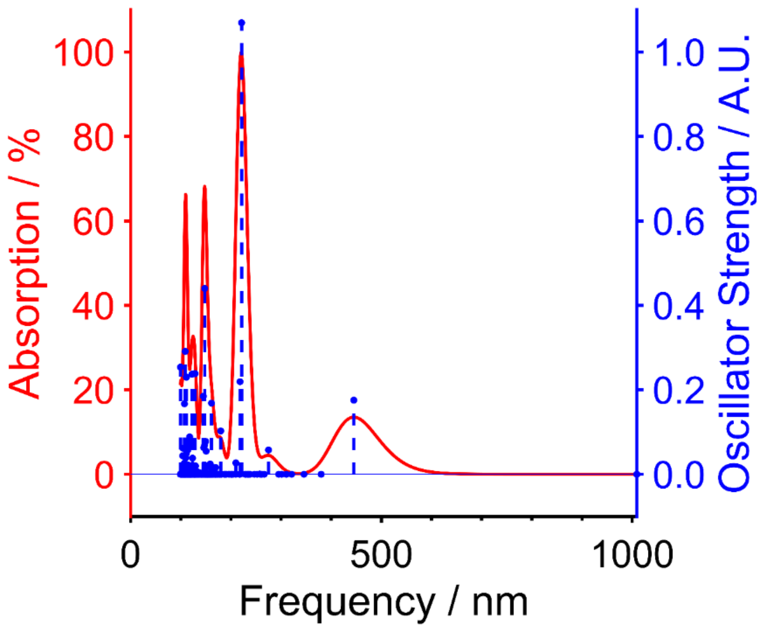

Figure S 35: Calculated spectrum for ***2***$^{4-}$ *at the CAM-B3LYP/TZVP level of theory (TD-DFT, 50:50 singlet: triplet, no. states = 200).*

Experimental parameters

- The battery was operated between 0.40 and 1.75 V at a current of 20 mA (4 mA.cm^{-2}).
- Limiting tank: 10 mM **2**, 11.7 ml
- Non-limiting tank: 30 mM $K_4Fe(CN)_6.3H_2O$, 7.5 mM $K_3Fe(CN)_6$, 12.0 ml.
- Two layers Nafion 212 pre-soaked in 0.1 M LiOH.
- Flow rate = 80 rpm (25 ml.min^{-1})
- Carbon paper (3x per side, Sigracet GDL-39AA) pre-baked at 400 °C for 24 hrs was used as the electrode.
- UV-Vis Integration time = 0.350 ms
- UV-Vis Averaging = 200 scans
- UV-Vis Smoothing = 3 pixels
- UV-Vis spectrometer = 1908047U1

NMR and EPR Spectroscopy

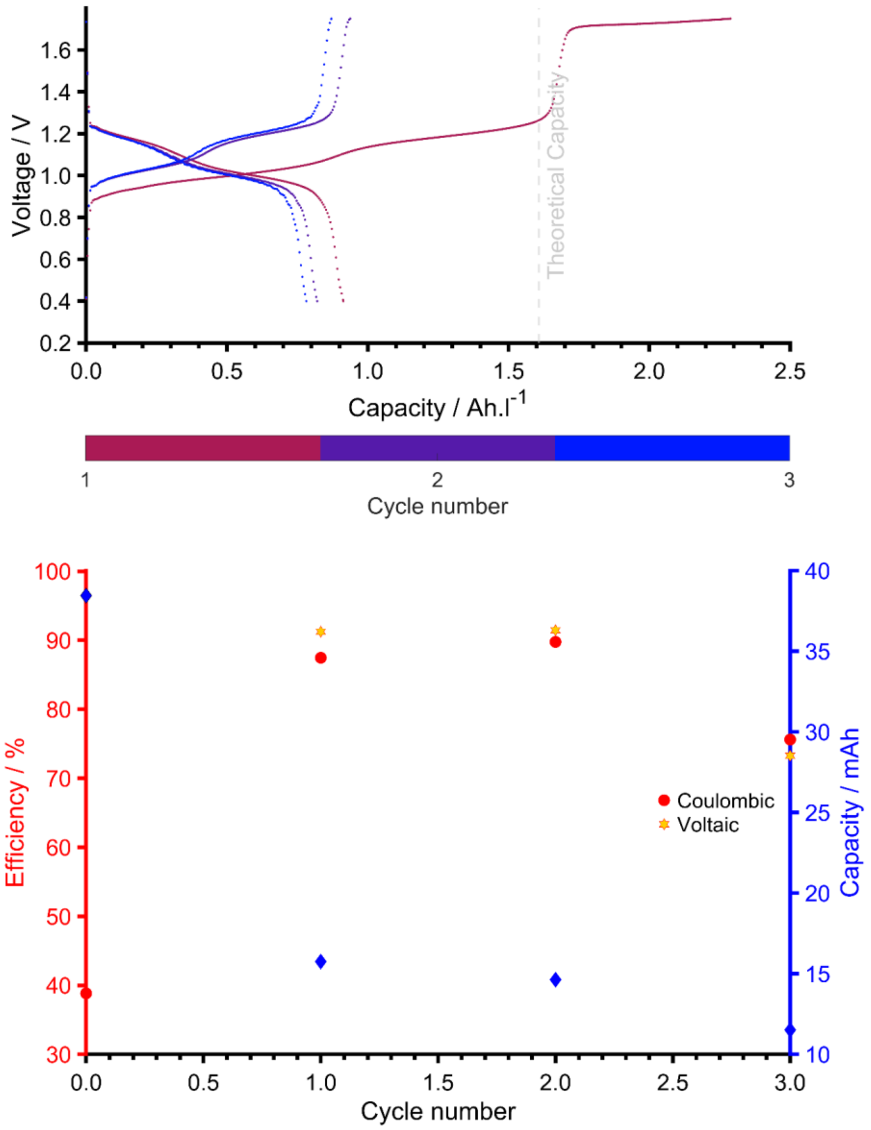

*Figure S 36: Galvanostatic cycling data corresponding to the in-situ NMR/EPR experiment. The lab-scale battery of **2** against potassium ferrocyanide in D_2O with 1 M LiOH added as the supporting electrolyte was run at ambient temperature under an inert nitrogen atmosphere. The battery was operated between 0.40 and 1.75 V at a current of 30 mA (6 mA.cm^{-2}). Limiting tank: 30 mM **2**, 17.0 ml; non-limiting tank:150 mM $K_4Fe(CN)_6.3H_2O$, 37.5 mM $K_3Fe(CN)_6$, 15.1 ml. Two layers Nafion 212 pre-soaked in 0.1 M LiOH. Flow rate = 80 rpm (20 ml.min^{-1}). Carbon paper (3x per side, Sigracet GDL-39AA) pre-baked at 400 °C for 24 hrs was used as the electrode.*

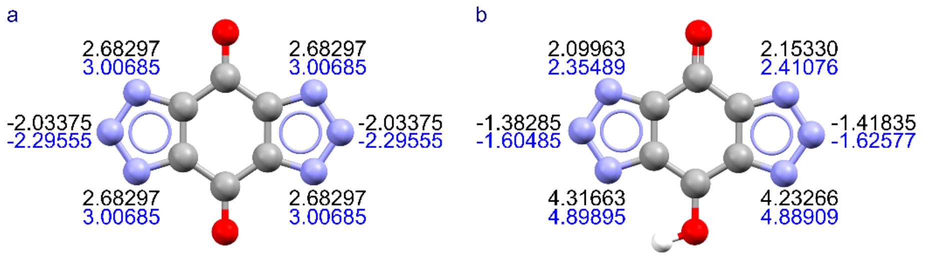

Figure S 37: Fermi contact coupling constants for each of the nitrogen atoms in (a) $\mathbf{2}^{3-\cdot}$ *and (b)* $\mathbf{2}^{2-\cdot}$*. The blue text denotes the values calculated in MHz at the B3LYP/EPR-III level of theory while the black text denotes the values calculated at the B3LYP/TZVP level of theory.*

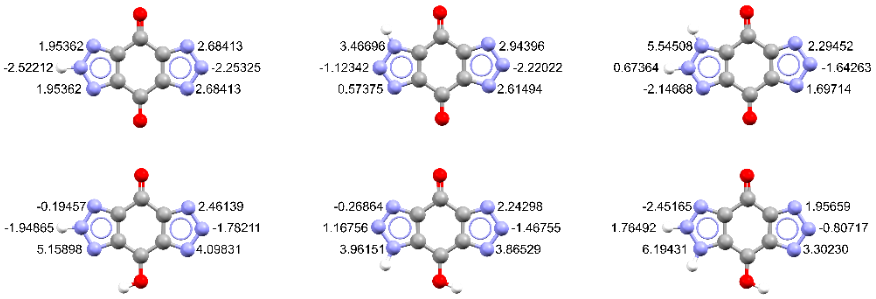

Figure S 38: Fermi contact coupling constants for each of the nitrogen atoms in a variety of protonated forms of ***2****. The black text denotes the values calculated in MHz at the B3LYP/TZVP level of theory.*

Table S 10: Table listing the anisotropic g and A values for the species depicted in Figure S 37 and Figure S 38calculated by DFT at the B3LYP/EPRIII level of theory. Note: N_{ax} refers to the central nitrogen atom of the triazole ring i.e., the nitrogen that lies on the axis of symmetry.

**Property**	$2^{3-\cdot}$	$2^{2-\cdot}_{H@O}$	$2^{2-\cdot}_{H@N}$	$2^{2-\cdot}_{H@Nax}$	$2^{1-\cdot}_{H@N, Nax}$	$2^{\cdot}_{H@O, N, Nax}$	$2^{1-\cdot}_{H@O, N}$	$2^{1-\cdot}_{H@O, Nax}$
**g relative to g_{free} / ppm**	[-137.2 3032.3 3968.1]	[-111.1 2541.7 2893.9]	[-162.1 3161.6 4582.3]	[-145.6 3141.9 4484.5]	[-228.5 2999.0 4614.3]	[-179.5 2156.5 3234.6]	[-148.7 2458.9 3390.5]	[-131.7 2625.1 2909.1]
**A1 / MHz**	[-3.354 -3.168 6.522]	[-5.558 -5.247 10.806]	[-3.312 -3.124 6.436]	[-3.372 -3.191 6.563]	[-3.086 -2.892 5.978]	[-4.463 -4.393 9.036]	[-5.050 -4.787 9.837]	[-3.028 -2.883 5.911]
**A2 / MHz**	[-3.983 1.920 2.063]	[-2.590 1.179 1.411]	-4.001 1.936 2.065	[-4.595 2.213 2.381]	[-3.458 1.672 1.786]	[-1.442 0.618 0.824]	[-2.833 1.301 1.532]	[-3.425 1.587 1.838]
**A3 / MHz**	[-3.354 -3.168 6.522]	[-2.649 -2.471 5.120]	[-2.904 -2.741 5.645]	[-3.372 -3.191 6.563]	[-2.059 -1.940 3.999]	[-2.410 -2.260 4.670]	[-2.812 -2.651 5.463]	[-5.497 -5.209 10.707]
**A4 / MHz**	[-3.354 -3.168 6.522]	[-2.729 -2.539 5.268]	[-0.816 -0.637 1.453]	[-2.796 -2.551 5.347]	[-4.248 2.100 2.149]	[-5.072 2.459 2.613]	[-0.319 0.027 0.292]	[-6.612 -6.189 12.801]
**A5 / MHz**	[-3.983 1.920 2.063]	[-2.537 1.148 1.388]	[-2.204 1.078 1.126]	[-3.011 1.477 1.533]	[-1.787 -1.399 3.187]	[-2.784 -2.482 5.266]	[-1.207 -1.066 2.273]	[-1.305 0.572 0.733]
**A6 / MHz**	[-3.354 -3.168 6.522]	[-5.663 -5.352 11.016]	[-3.970 -3.695 7.665]	[-2.796 -2.551 5.347]	[-8.142 -7.681 15.823]	[-8.046 -7.603 15.649]	[-4.664 -4.354 9.018]	[-0.229 -0.095 0.323]

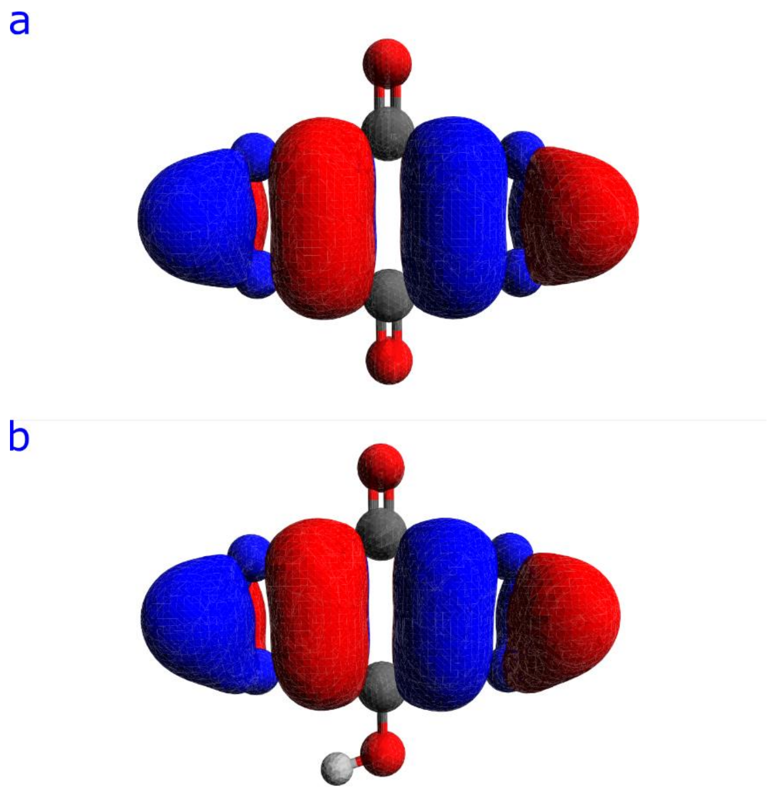

Figure S 39: (a) SOMO of $\mathbf{2}^{3-\cdot}$ *(b) SOMO of* $\mathbf{2}^{2-\cdot}$.

Experimental Parameters

- The battery was operated between 0.40 and 1.75 V at a current of 30 mA (6 $mA.cm^{-2}$).
- Limiting tank: 30 mM **2**, 17.0 ml
- Non-limiting tank: 150 mM $K_4Fe(CN)_6.3H_2O$, 37.5 mM $K_3Fe(CN)_6$, 15.1 ml.
- Two layers of Nafion 212 pre-soaked in 0.1 M LiOH were used as the membrane.
- Flow rate = 80 rpm (20 $ml.min^{-1}$).
- Carbon paper (3x per side, Sigracet GDL-39AA) pre-baked at 400 °C for 24 hrs was used as the electrode.

Anisotropic EPR simulation parameters

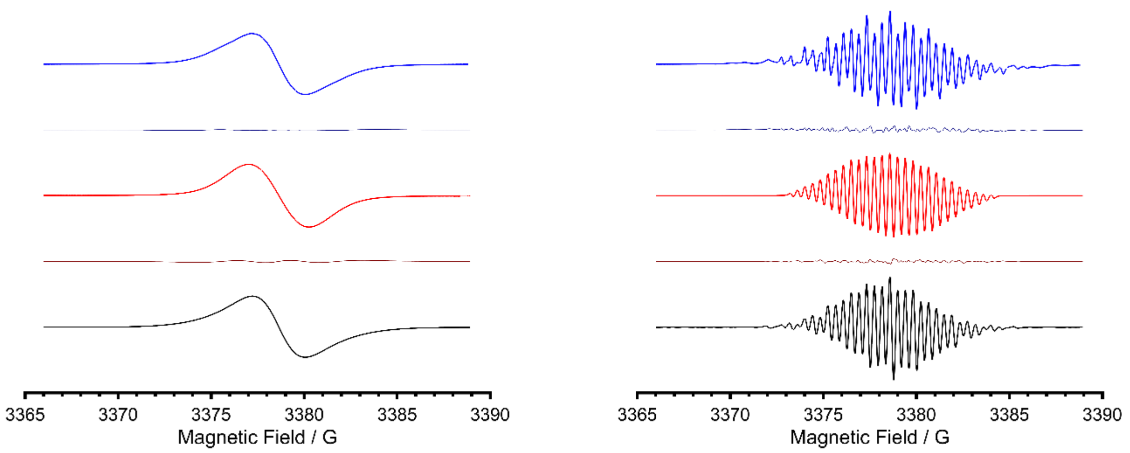

Figure S 40: Normalised EPR spectra extracted at the (left) peak radical concentration (end of first plateau) and at the (right) end of charge (just before end of second plateau). The experimental spectra (black) are compared to a simulated spectrum under an isotropic tumbling regime (red) and under the fast-motion regime but with slower tumbling (blue). The spectra on the left and right correspond to ζ and #, respectively. The residual for each simulated spectrum is shown underneath the respective simulation.

The EPR spectra were fit using Voigtian (a combination of both Gaussian and Lorentzian broadening) lineshapes where appropriate.

Listed below are the results obtained from the isotropic simulations.

Table S 11: Isotropic EPR fitting of the high-concentration spectrum (ζ) recorded during the in situ EPR experiment.

Property	Result
**Frequency / GHz**	9.472
**Temperature / °C**	29.9
**g**	2.002985
**A1 / MHz**	0.733
**A2 / MHz**	0.205
**A3 / MHz**	0.644
**A4 / MHz**	0.441
**A5 / MHz**	0.555
**A6 / MHz**	0.792
**Gaussian linewidth / mT**	0.270
**Lorentzian linewidth / mT**	0.0724

Table S 12: Isotropic EPR fitting of the low-concentration spectrum (#) recorded during the in situ EPR experiment.

Property	Result
**Frequency / GHz**	9.472
**Temperature / °C**	30.0
**g**	2.002970
**A1 / MHz**	5.815
**A2 / MHz**	3.511
**A3 / MHz**	2.240
**A4 / MHz**	1.136
**A5 / MHz**	1.358
**A6 / MHz**	1.005
**Gaussian linewidth / mT**	0.0133
**Lorentzian linewidth / mT**	0.0100

Easyspin[8] was used to simulate the spectra assuming an asymmetric g-tensor as a starting point. Listed below are the results obtained from the anisotropic simulations i.e., when the rate of tumbling of the molecule impacts the EPR spectrum. An anisotropic g-tensor of [2.00299 2.00300 2.00301] was provided as an initial estimate to obtain the results below.

Table S 13: Anisotropic EPR fitting of the high-concentration spectrum (ζ) recorded during the in situ EPR experiment.

**Property**	**x**	**y**	**z**
**Frequency / GHz**	9.472		
**Temperature / °C**	29.9		
**g**	2.003085	2.003281	2.002555
**A1 / MHz**	8.246	0.267	3.447
**A2 / MHz**	2.262	0.139	1.278
**A3 / MHz**	2.367	0.640	1.549
**A4 / MHz**	8.318	0.431	3.162
**A5 / MHz**	0.780	0.204	1.051
**A6 / MHz**	2.885	0.698	2.040
**Gaussian linewidth / mT**	0.0450		
**Lorentzian linewidth / mT**	6.027×10^{-4}		
**Log_{10}(time correction / s)**	-7.781		

Table S 14: Anisotropic EPR fitting of the low-concentration spectrum (#) recorded during the in situ EPR experiment.

Property	x	y	z
**Frequency / GHz**	9.472		
**Temperature / °C**	30.0		
**g**	2.002528	2.002450	2.002813
**A1 / MHz**	5.382	6.620	5.285
**A2 / MHz**	4.225	9.995	4.036
**A3 / MHz**	5.247	3.560	1.939
**A4 / MHz**	2.901	4.826	2.682
**A5 / MHz**	2.998	9.471	3.657
**A6 / MHz**	4.210	6.125	3.680
**Gaussian linewidth / mT**	0.00549		
**Lorentzian linewidth / mT**	8.880×10^{-6}		
**Log_{10}(time correction / s)**	-7.981		

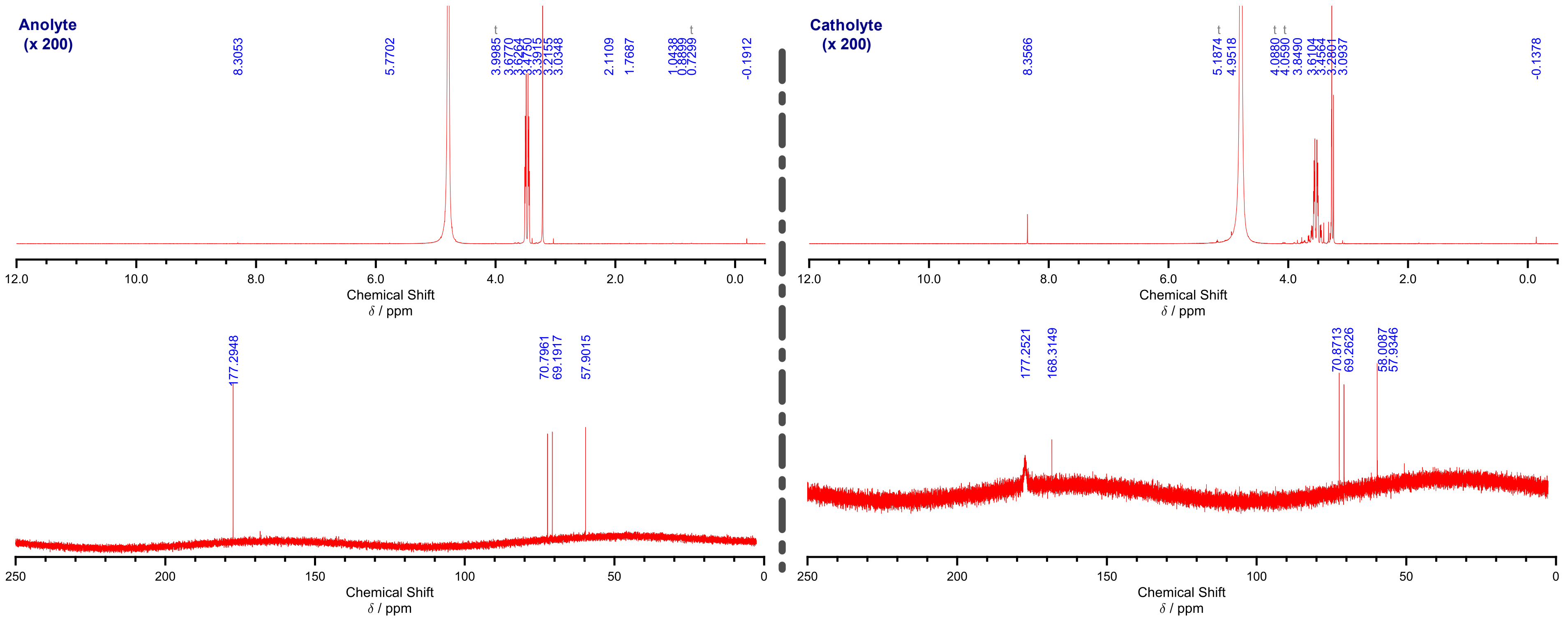

*Figure S 41: ^{1}H (above) and ^{13}C NMR (below) of the anolyte tank containing **2** (left) and the catholyte tank (right) containing **2**$^{2-}$ after battery cycling. The battery cycling data is presented in Figure 6 of the main matter. The peak at 4.79 ppm in the ^{1}H NMR corresponds to the HOD peak in D_2O while the peak below 0 ppm can be attributed to silicon grease. The intensity of the ^{1}H spectra was enhanced 200-fold. The carbon spectra are presented without any additional scaling.*

Symmetric Cell

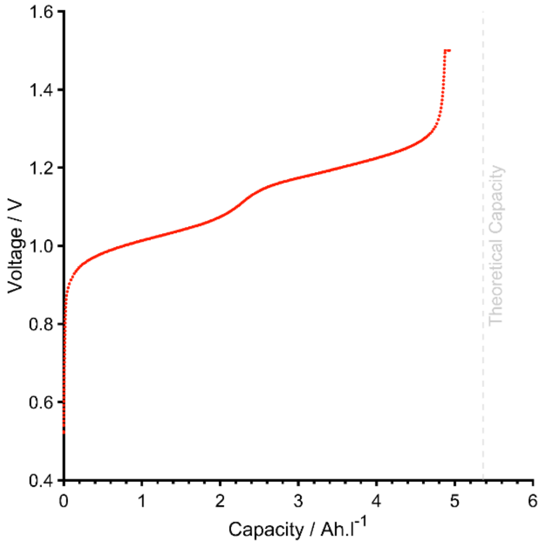

*Figure S 42: Lab-scale symmetric cell charge cycle of **2** against potassium ferrocyanide in D_2O with 1 M LiOH added as the supporting electrolyte at ambient temperature under an inert nitrogen atmosphere. The battery was charged at a current of 200 mA (40 mA.cm^{-2}). Limiting tank: 100 mM **2**, 13.2 ml; non-limiting tank:300 mM $K_4Fe(CN)_6.3H_2O$, 75 mM $K_3Fe(CN)_6$, 20.2 ml. Two layers Nafion 212 pre-soaked in 0.1 M LiOH. Flow rate = 80 rpm (50 ml.min^{-1}). Carbon paper (3x per side, Sigracet GDL-39AA) pre-baked at 400 °C for 24 hrs was used as the electrode.*

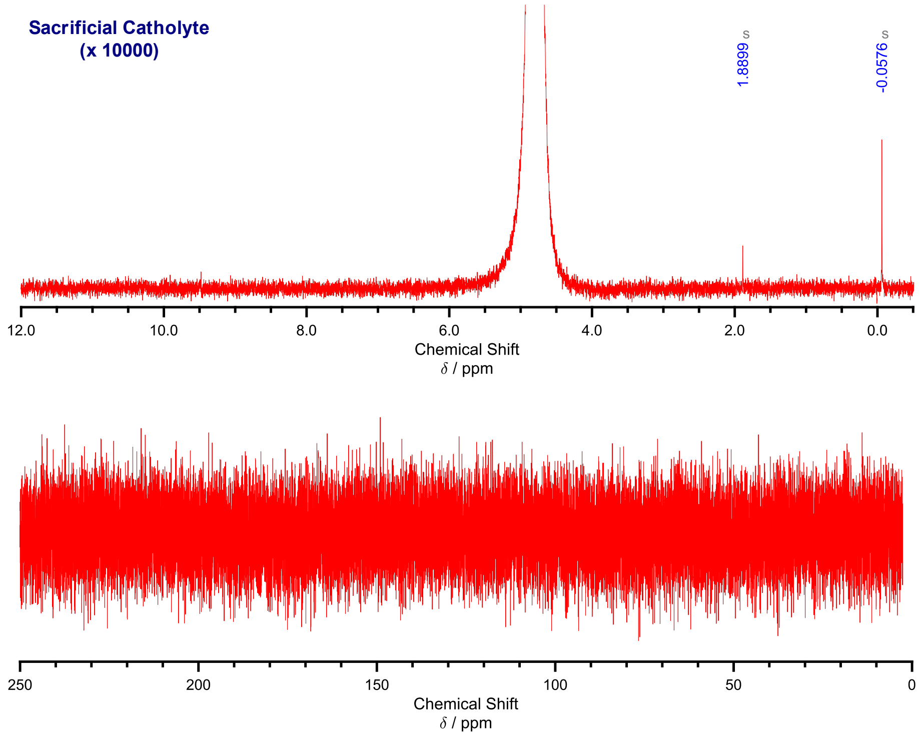

*Figure S 43: ^{1}H (above) and ^{13}C NMR of the sacrificial hexacyanoferrate tank that was used to charge a tank of **2** for symmetric cycling. The peak at 4.79 ppm in the ^{1}H NMR corresponds to the HOD peak in D_2O while the peak below 0 ppm can be attributed to silicon grease. The intensity of the ^{1}H spectrum was enhanced 10 000-fold. The carbon spectrum is presented without any additional scaling.*

Specific area resistance (two Nafion 212 membranes) = 2.0483 ± 0.006 Ωcm^2 (charge: 2.0457 ± 0.006 Ωcm^2, discharge: 2.051 ± 0.01 Ωcm^2).

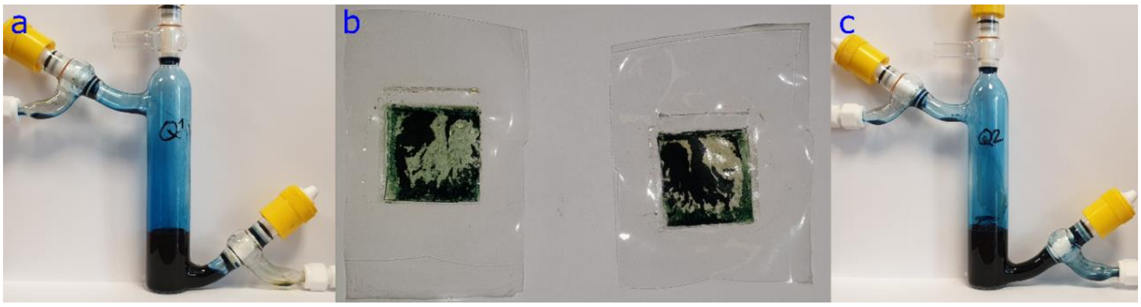

Figure S 44: Photographs of (a) the capacity limiting tank, (b) the membranes, (c) the non-limiting tank after completion of the symmetric cell experiment.

Experimental Parameters

- The battery was operated between -0.45 and 0.45 V at a current of 200 mA.
- Limiting tank: 100 mM **2**, 13.2 ml
- Non-limiting tank: 100 mM **2**, 15.0 ml.
- Two layers Nafion 212 pre-soaked in 0.1 M LiOH. Flow rate = 80 rpm (50 $ml.min^{-1}$).
- Carbon paper (3x per side, Sigracet GDL-39AA) pre-baked at 400 °C for 24 hrs was used as the electrode.
- The capacity limiting tank was charged against 300 mM $K_4Fe(CN)_6.3H_2O$, 75 mM $K_3Fe(CN)_6$, 20.2 ml.

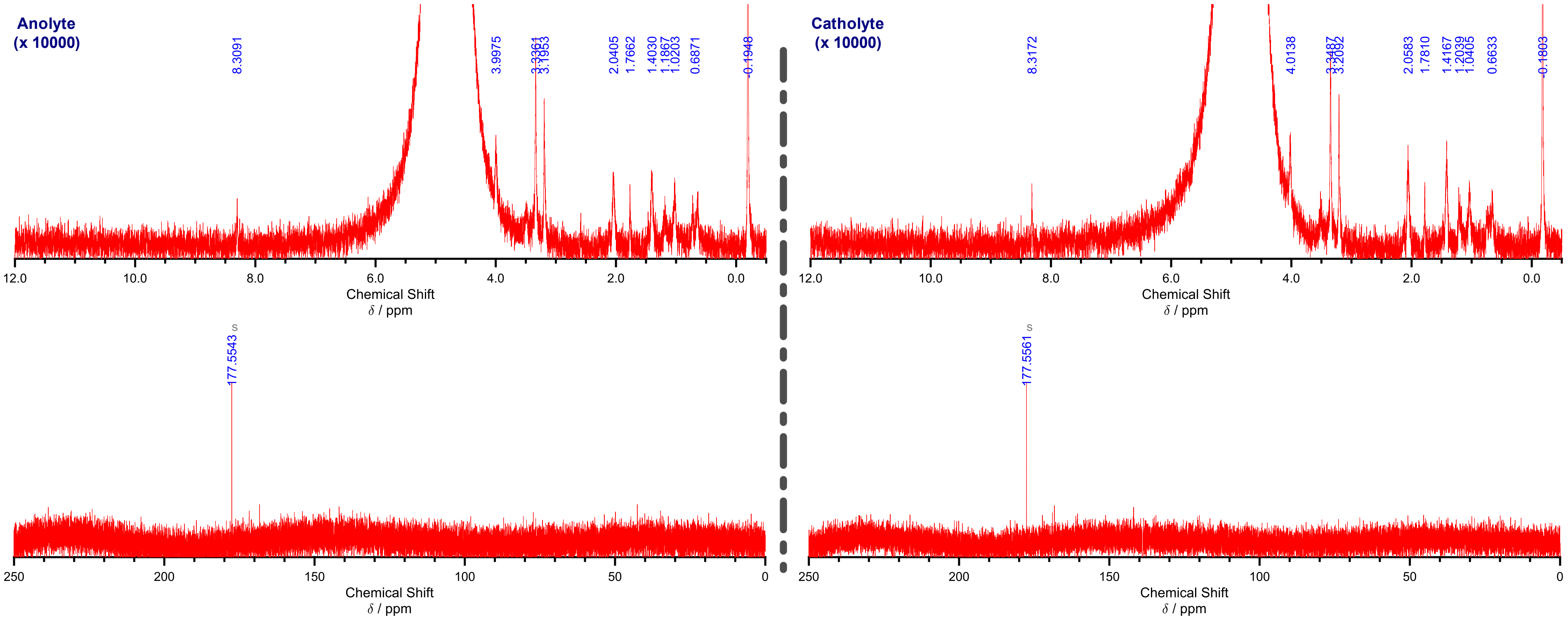

*Figure S 45: ^{1}H (above) and ^{13}C NMR (below) of the anolyte tank containing **2** (left) and the catholyte tank (right) containing **2**$^{2-}$ after battery cycling. The battery cycling data is presented in Figure 7 of the main matter. The peak at 4.79 ppm in the ^{1}H NMR corresponds to the HOD peak in D_2O while the peak below 0 ppm can be attributed to silicon grease. The intensity of the ^{1}H spectra was enhanced 10 000-fold. The carbon spectra are presented without any additional scaling.*

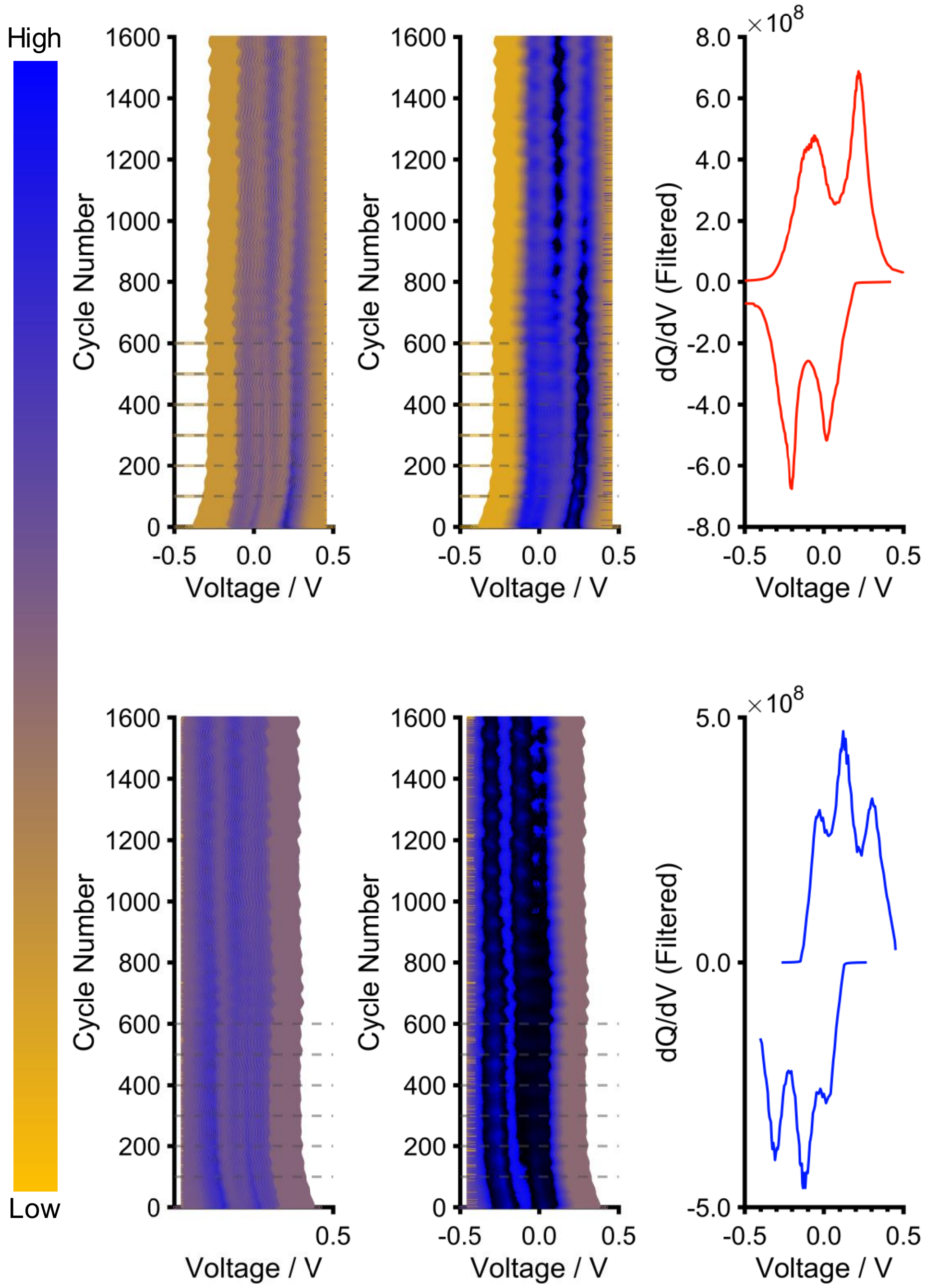

*Figure S 46: dQ/dV plots corresponding to the symmetric cell performance of **2** in D_2O with 1 M LiOH added as the supporting electrolyte at ambient temperature under an inert nitrogen atmosphere. The left-most graphs reflect the numerically calculated values from the raw data, the middle graphs show the data smoothed with a Savitzky-Golay filter (top: charge, bottom: discharge). Top-right shows the dQ/dV from the first cycle while the bottom-right shows the dQ/dV from the last cycle.*

Synthesis

2,3,5,6-tetraaminocyclohexa-2,5-diene-1,4-dione (**1**)

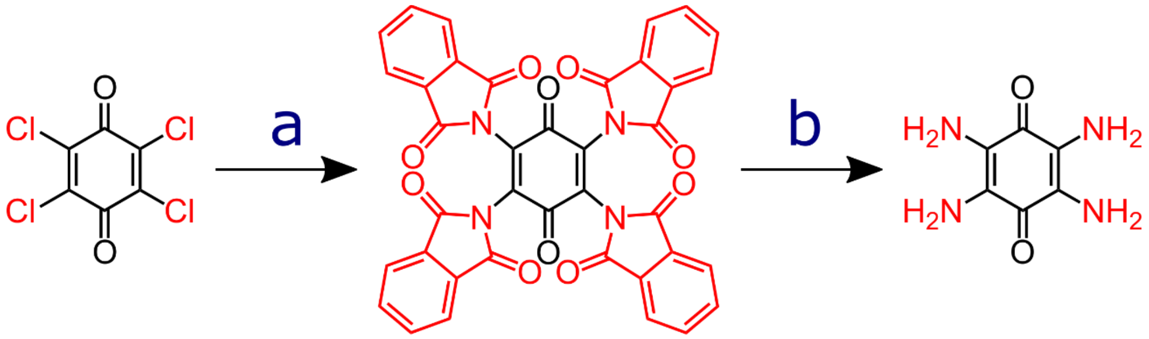

*Scheme S 1: Synthetic scheme for the synthesis of **1**. (a) Potassium phthalimide, acetonitrile (dry), Δ (b) Hydrazine hydrate, Δ.*

Synthesis of **1** was carried out as described in literature.[32–34]

Under an inert nitrogen atmosphere, 2,3,5,6-tetrachloro-benzo-1,4-quinone (20.70 g, 84.17 mmol) and potassium phthalimide (79.02 g, 426.67 mmol) were mixed. Acetonitrile (dry, degassed, 300 ml) was then added. The suspension was then heated at reflux (90ºC, 24 h) and it took on a dark green colour. The suspension was then allowed to cool to room temperature and filtered under vacuum. The filtrate had a dark-red colour while the solid was dark purple/brown. The solid was washed onto the filter with acetonitrile and washed further with water. The solid was then dissolved in N, N-dimethylformamide under heat (100 °C, 1.5 h). The suspension was then hot filtered and washed with water (Soxhlet) until the filtrate was colourless. The brown solid residue was then washed with ethanol (Soxhlet) and filtered once more under vacuum. The solid sample was then added to an aqueous solution of hydrazine hydrate (64%, 100 ml) and heated (65 °C, 3 h). The dark purple suspension was filtered under vacuum and washed with copious amounts of water to give the desired product as a dark purple/black crystalline powder (5.868 g, 34.89 mmol, 41% yield).

A sample was sublimed according to the literature purification.[28] However, the crystal yield was low (0.195 g from 3.387 g crude, 5.76% by mass) so the crude was utilised as synthesised in all reactions listed below.

Elemental Analysis (Crude) / %: Predicted: C 42.86, H 4.80, N 33.32; Observed: C 41.9, H 4.6, N 31.4.

^{1}H NMR (400.13 MHz, 298 K, Figure S 47, d_6-DMSO): Crude: δ 4.54 ppm (A, 8H, s); Sublimed: δ 4.54 ppm (A, 8H, s).

^{13}C NMR (100.61 MHz, 298 K, d_6-DMSO): Crude: δ 179.3 (C=O), 121.8 ppm (C-NH_2); Sublimed: δ 179.3 (C=O), 121.8 ppm (C-NH_2).

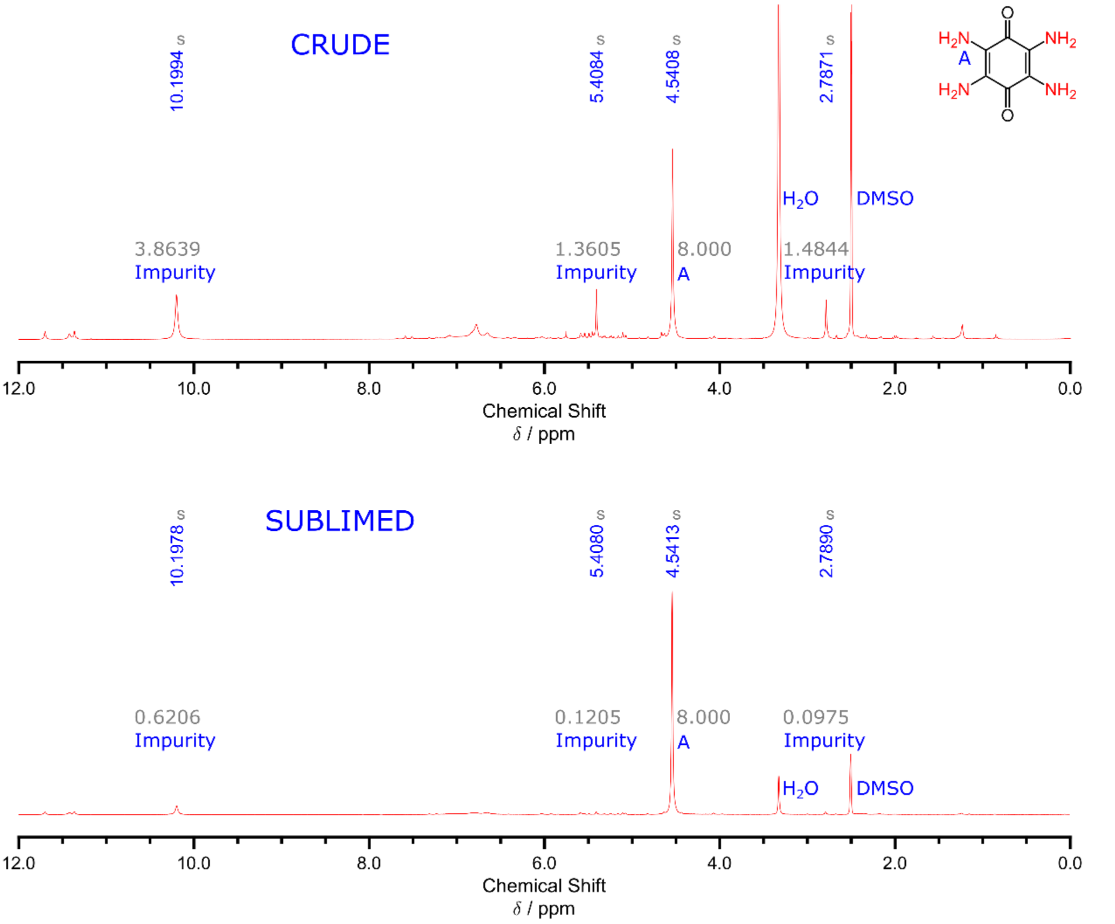

*Figure S 47: ^{1}H NMR spectra of **1** in deuterated dimethyl sulphoxide (DMSO). (top) Crude (400.13 MHz, 298 K) (bottom) Sublimed (400.03 MHz, 298 K).*

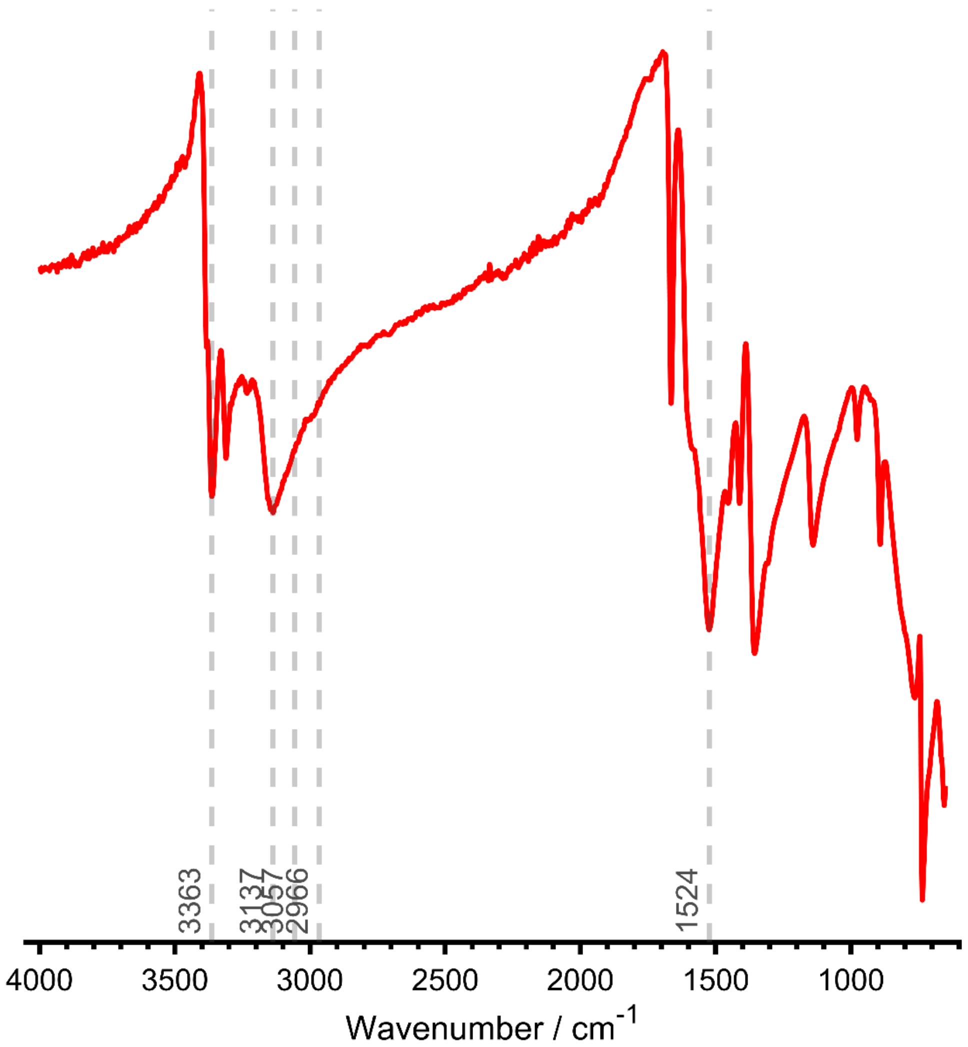

*Figure S 48: ATR-IR spectrum of **1** (Crude).*

Sodium 4,8-dioxo-4,8-dihydrobenzo[1,2-d:4,5-d']bis([1,2,3]triazole)-1,5-diide (**2**)

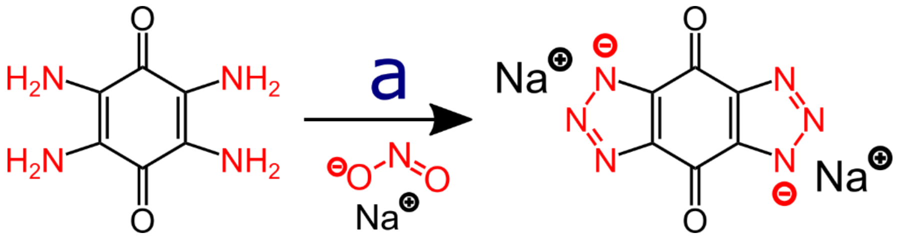

Scheme S 2: Synthetic scheme for the synthesis of ***2*** *(a) sodium nitrite (0.8 M aqueous solution), glacial acetic acid.*

While the synthetic route reported by Bunzen *et al.* reportedly gives higher yields,[30] the protocol described by Sato *et al.* was followed as it used **1** as a starting material.[28]

1 (997 mg, 5.93 mmol) was added to glacial acetic acid (25 ml) to form a black suspension. Sodium nitrite (1.46 g, 21.16 mmol) was dissolved in water (deionised, 26.5 ml, 0.8 M) at 0 °C and then added to the suspension of **1** dropwise with stirring. The suspension rapidly turned brown and was left to stir for 24 h. The crude product was isolated by filtration as a green/brown powder. The powder was dissolved in the minimum volume of water and hot-filtered to give a yellow solution. On cooling, yellow crystalline needles were formed (495 mg, 2.11 mmol, 36% yield).

Elemental Analysis / %: Predicted: C 30.79, H 0.00, N 35.90; Observed: C 32.1, H 2.3, N 35.7.

^{1}H NMR (400.13 MHz, 298 K, Figure S 49, d_6-DMSO): No peaks expected.

^{13}C NMR (100.61 MHz, 298 K, d_6-DMSO): δ 171.2 (C=O), 145.3 ppm (C-N).

Note: To prepare sufficient material for battery characterisation, the above protocol was repeated several times, with several recrystallizations being required in some cases. Therefore, for each battery run a separate batch of sample was prepared.

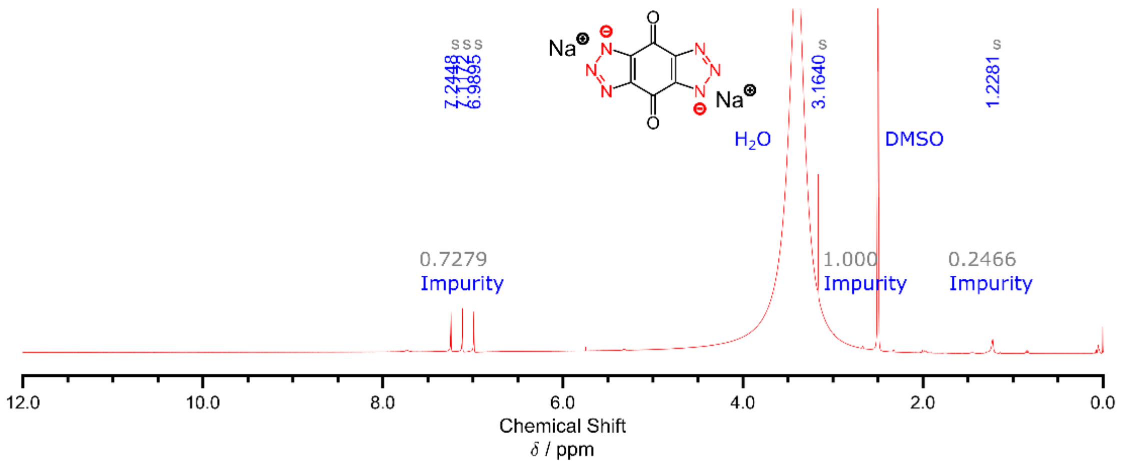

*Figure S 49: ^{1}H NMR spectrum of **2** (400.13 MHz, 298 K) in deuterated dimethyl sulphoxide (DMSO).*

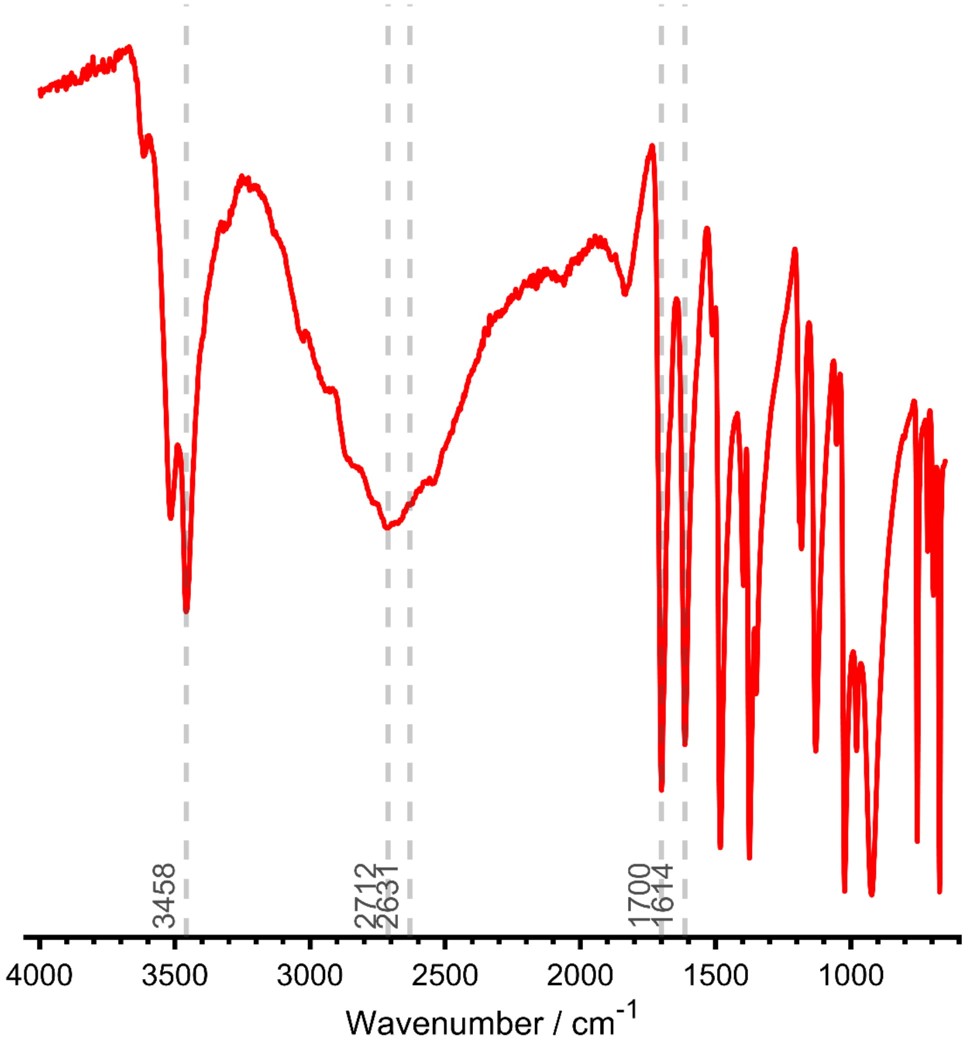

*Figure S 50: ATR-IR spectrum of **2**.*

4H,8H-benzo[1,2-c:4,5-c']bis([1,2,5]thiadiazole)-4,8-dione (**3**)

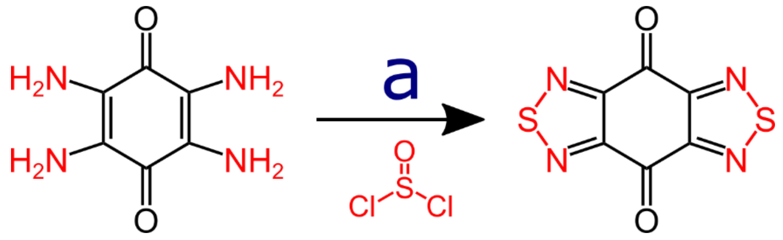

Scheme S 3: Synthetic scheme of the synthesis of ***3*** *(a) thionyl chloride, Δ.*

The synthetic protocol described by Neidlein was followed.[35,36]

1 (262 mg, 1.56 mmol) was added to thionyl chloride (distilled over quinoline, 2.9 ml, 39.98 mmol) under an inert atmosphere. The dark red suspension was heated (80 °C, 22.5 h) to form a brown suspension. The excess thionyl chloride was removed under vacuum to yield a light brown powder (357 mg) as crude. A sample (207 mg) was dissolved in N, N-dimethylformamide and filtered. The filtrate was added to activated charcoal and filtered again. The filtrate was then collected and heated to redissolve the product. The solution was then held at 5 °C until light-brown crystalline plates formed. The solvent was decanted, and the sample collected by filtration under vacuum. Copious amounts of water and ether were used to wash the crystals (86 mg, 0.38 mmol, 43% yield). A sample was analysed via single-crystal X-ray diffraction (orange plate, Figure S 51, Table S 15).

Elemental Analysis / %: Predicted: C 32.14, H 0.00, N 24.99; Observed: C 32.1, H 0.0, N 24.9.

^{1}H NMR (400.03 MHz, 298 K, Figure S 52, d_6-DMSO): No peaks expected.

^{13}C NMR (100.59 MHz, 298 K, d_6-DMSO): δ 169.3 (C=O), 158.5 ppm (C-N).

*Table S 15: Details of the data collections and refinements for **3**.*

	**3**
**CCDC number**	2287481
**Cambridge data number**	DW_B2_0304
**Chemical formula**	$C_6N_4O_2S_2$
**Formula weight**	224.22
**Temperature / K**	180(2)
**Crystal system**	monoclinic
**Space group**	P 2_1/n
**a / Å**	8.7904(6)
**b / Å**	5.5796(4)
**c / Å**	8.7930(6)
**alpha / °**	90
**beta / °**	119.563(2)
**gamma / °**	90
**Unit-cell volume / Å^3**	375.12(5)
**Z**	2
**Calc. density / g cm^{-3}**	1.985
**F(000)**	224
**Radiation type**	Cu Kα
**Absorption coefficient / mm^{-1}**	6.280
**Crystal size / mm^3**	0.30 x 0.30 x 0.05
**2-Theta range / °**	11.57-133.34
**Completeness to max 2-theta**	0.987
**No. of reflections measured**	3632
**No. of independent reflections**	658
**R(int)**	0.0454
**No. parameters / restraints**	65 / 0
**Final R1 values (I > 2σ(I))**	0.0349
**Final wR(F^2) values (all data)**	0.0932
**Goodness-of-fit on F^2**	1.070
**Largest difference peak & hole / e Å^{-3}**	0.403, -0.582

The structure is refined as a 2-component twin, with twin law [0 0 1 | 0 -1 0 | 1 0 0] and refined BASF = 0.404(3). The structure is monoclinic with an approximate hexagonal metric (a ≈ c, beta ≈ 120°). The twin law represents a 2-fold rotation around [1 0 1]. The molecule is situated on a crystallographic inversion centre.

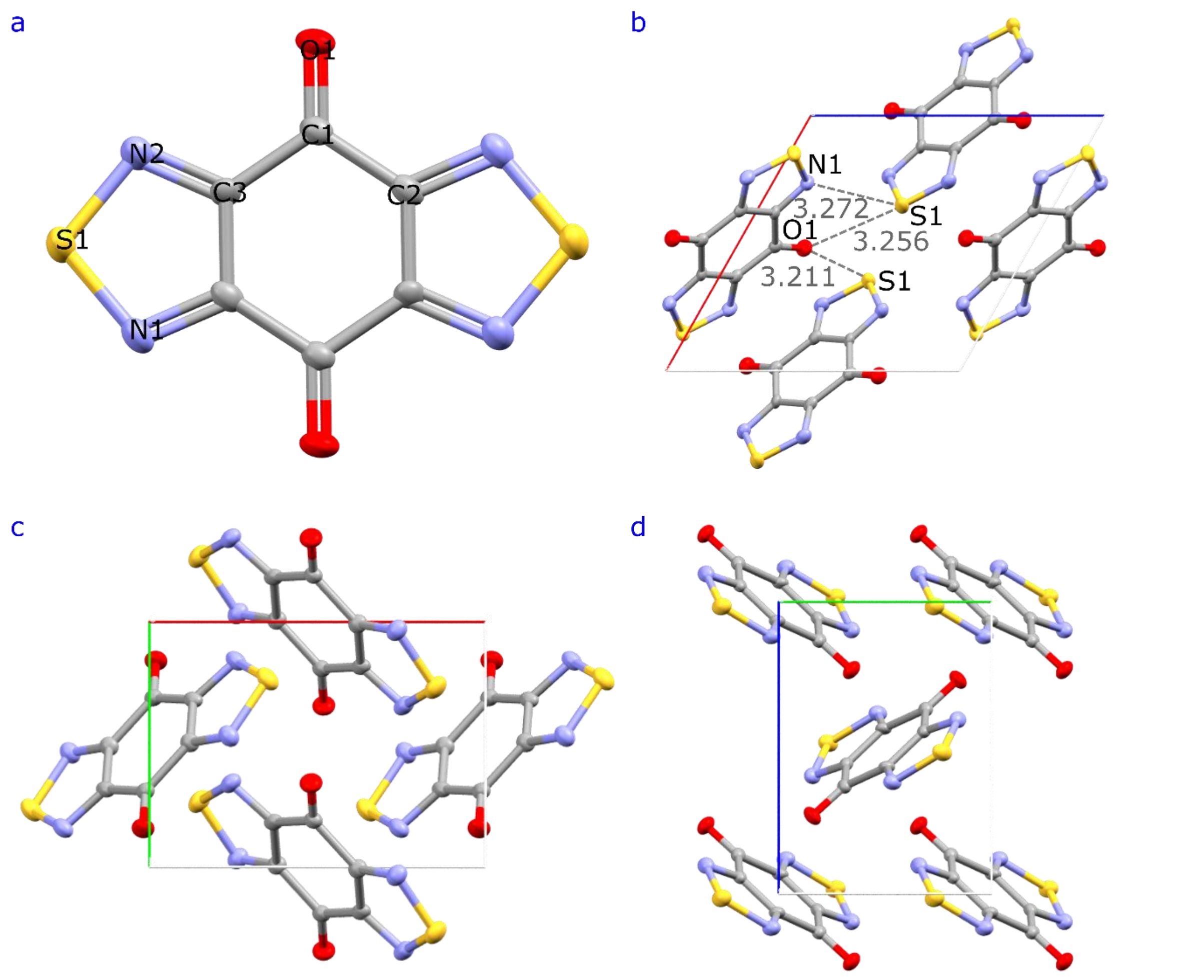

*Figure S 51: Crystal structure of **3** (a) bond lengths and dihedral angles, (b) unit cell viewed along the b-axis, (c) unit cell viewed along the c-axis, (d) unit cell viewed along the a-axis. Selected bond lengths and angles: C1-O1 = 1.209(4) Å, C1-C2 = 1.498(3) Å, C1-C3 = 1.483(5) Å, C2-C3 = 1.423(4) Å, C2-N1 = 1.319(4) Å, C3-N2 = 1.332(4) Å, N1-S1 = 1.622(2) Å, N2-S1 = 1.622(3) Å, C2-C1-C3 = 112.7(2)°, C2-C3-C1 = 124.1(2)°, C1-C2-C3 = 123.2(2)°. The CCDC number is 2287481.*

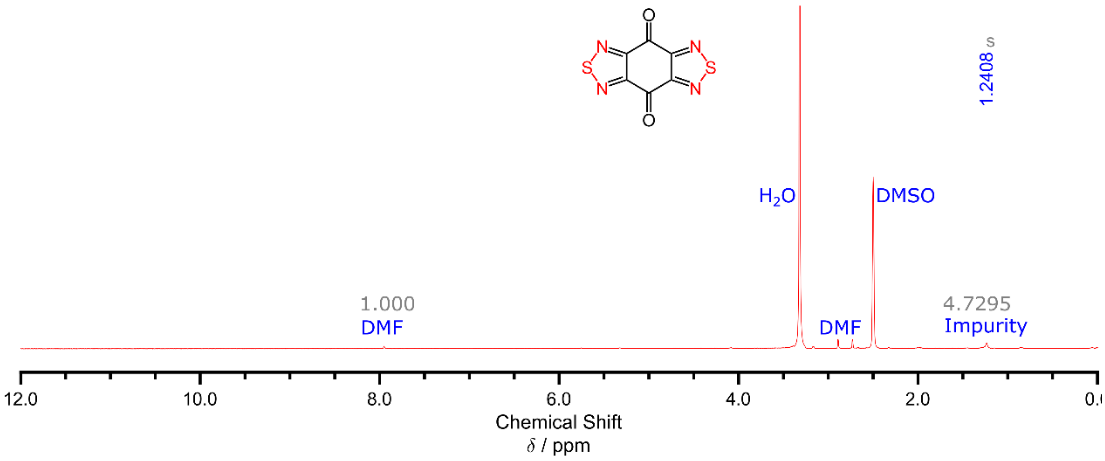

*Figure S 52: ^{1}H NMR spectrum of **3** (400.03 MHz, 298 K) in deuterated dimethyl sulphoxide (DMSO).*

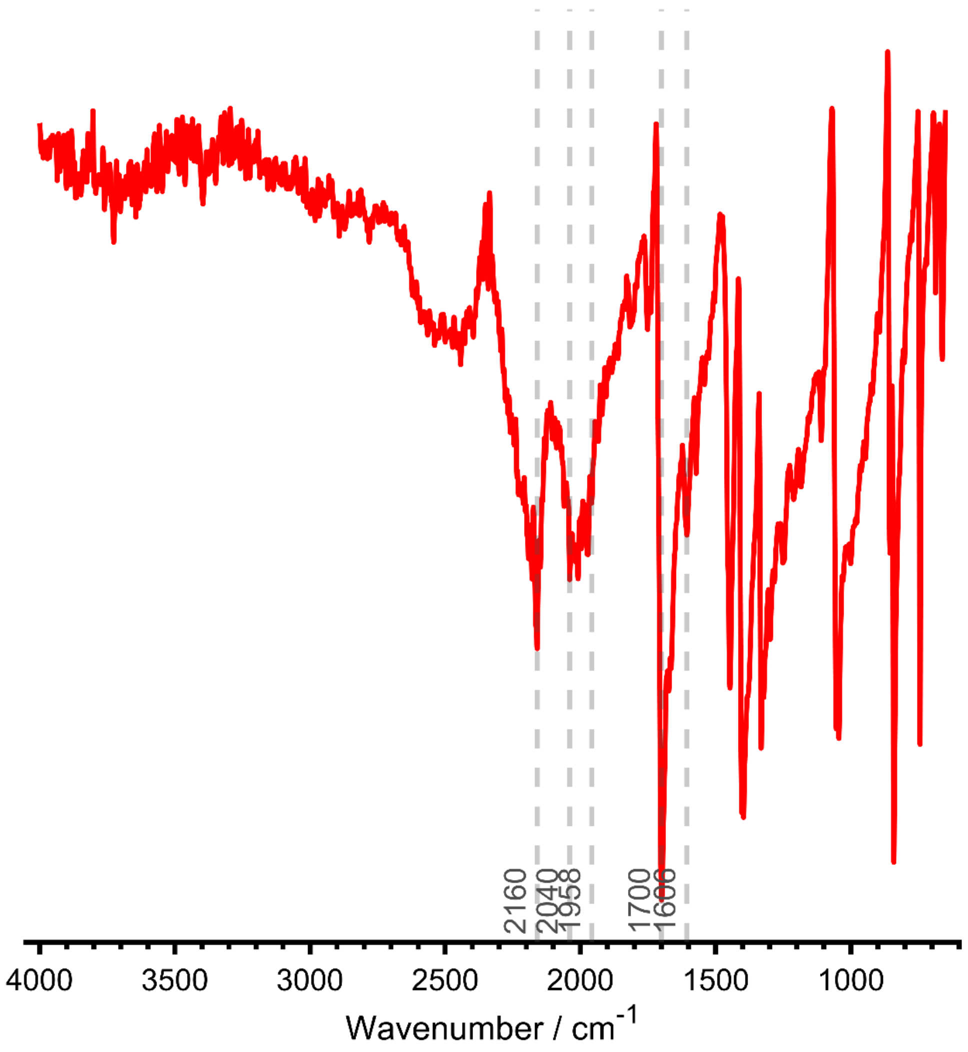

*Figure S 53: ATR-IR Spectrum of **3**.*

2,6-dimethylbenzo[1,2-d:4,5-d']diimidazole-4,8(1H,5H)-dione (**4**)

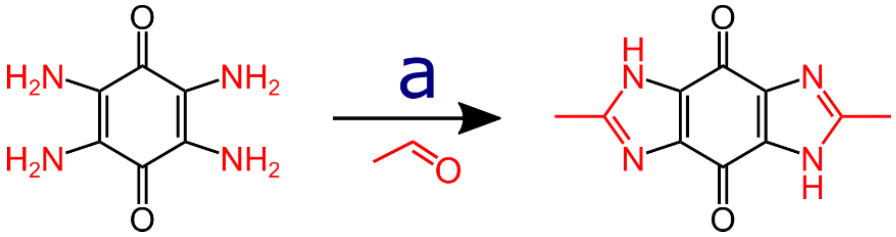

*Scheme S 4: Synthetic scheme of the synthesis of **4** (a) acetaldehyde, ethanol, Δ.*

The protocol described by Manivannan *et al.* was followed.[37]

1 (147 mg, 0.87 mmol) was added to acetaldehyde (0.1 ml, 1.78 mmol) and ethanol (5 ml) under a nitrogen atmosphere. The mixture was stirred at room temperature (4 h) before being heated at reflux (45 h). The suspension was then allowed to cool to room temperature and the product was collected by filtration under vacuum. A dark brown powder (128 mg, 0.59 mmol. 68% yield) was obtained.

^{1}H NMR (400.03 MHz, 298 K, Figure S 54, d_6-DMSO): δ 10.25 (B, 2H, s (br)), 2.35 ppm (A, 6H, s).

^{13}C NMR (100.59 MHz, 298 K, d_6-DMSO): δ 151.0 (C=O), 65.4 (NCN), 14.5 ppm (-CH_3).

MS (ES^+) = Calculated: 217.0726 Da; Observed: 217.0733 Da (3.22 ppm).

Note: satisfactory elemental analysis was not obtained.

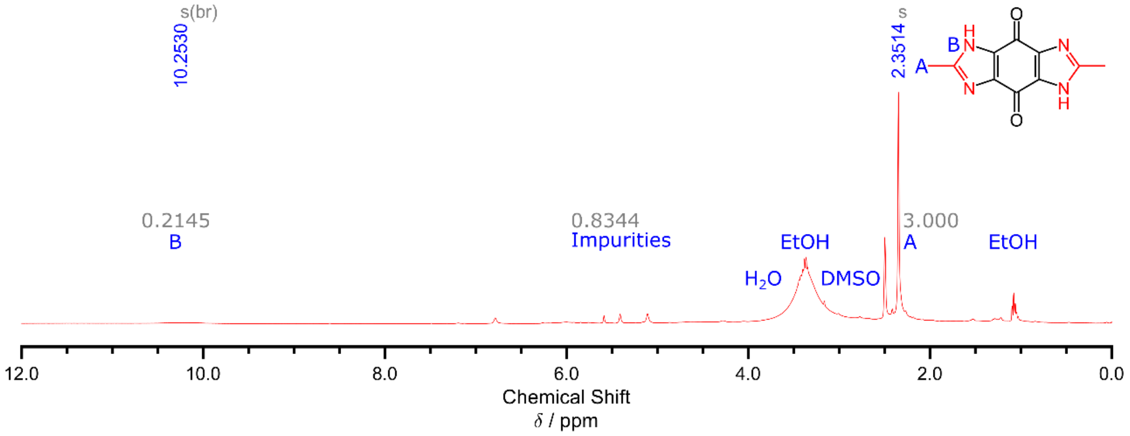

Figure S 54: ^{1}H NMR of ***4*** *(400.03 MHz, 298 K) in deuterated dimethyl sulphoxide (DMSO).*

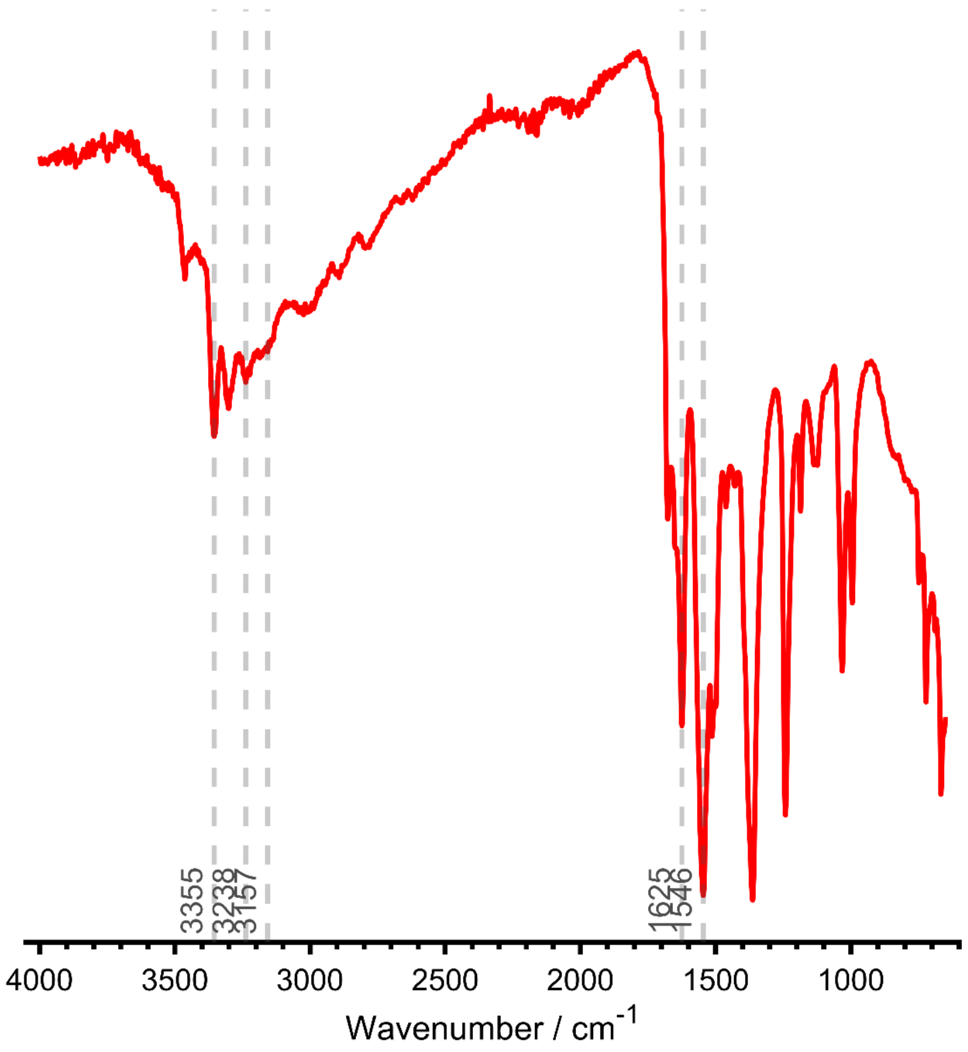

*Figure S 55: ATR-IR spectrum of **4**.*

2,6-di(pyridin-2-yl)benzo[1,2-d:4,5-d']diimidazole-4,8(1H,5H)-dione (**5**)

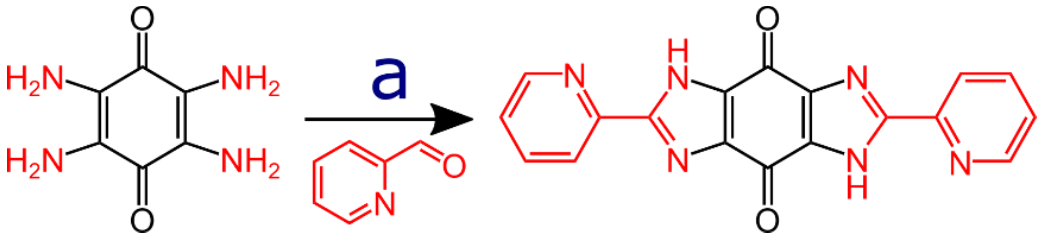

Scheme S 5: Synthetic scheme of the synthesis of ***5*** *(a) 2-pyridinecarboxaldehyde, tosic acid, N, N-dimethylacetamide, Δ.*

This molecule was previously reported in reference 38 but no synthetic protocol was provided. The protocol described by Karayannidis *et al.*[39] was modified. **1** (102 mg, 0.61 mmol), 2-pyridinecarboxaldehyde (0.12 ml, 1.26 mmol) and p-tosic acid (23 mg, 0.13 mmol) were added to N, N-dimethylacetamide (4 ml). The solution was heated (100 °C, 3 d) in the dark under an inert atmosphere. Methanol (10 ml) was then added to form a dark green suspension. The suspension was filtered to yield a yellow-green powder as the product (64 mg, 0.17 mmol, 31% yield).

^{1}H NMR (400.13 MHz, 298 K, Figure S 56, d_6-DMSO): 14.18 (E, 2H, s (br)), 8.71 (A, 2H, ddd, 4.8 Hz, 1.6 Hz, 0.9 Hz), 8.20 (D, 2H, ddd, 7.9 Hz, 1.1 Hz, 0.9 Hz), 7.99 (C, 2H, ddd, 7.9 Hz, 7.5 Hz, 1.6 Hz), 7.52 ppm (B, 2H, ddd, 7.5 Hz, 4.8 Hz, 1.1 Hz).

^{13}C NMR (100.61 MHz, 298 K, d_6-DMSO): 151.0 ($C_{Imidazole}$), 149.9 (C-Imidazole), 147.9 (C ortho to N), 138.1 (C para to N), 125.3 (C meta to N, para to imidazole), 122.1 ppm (C meta to N, ortho to imidazole). The quinone carbons were not observed.

MS (ES^+) = Calculated: 343.0943 Da; Observed: 343.0952 Da (2.62 ppm).

Note: satisfactory elemental analysis was not obtained.

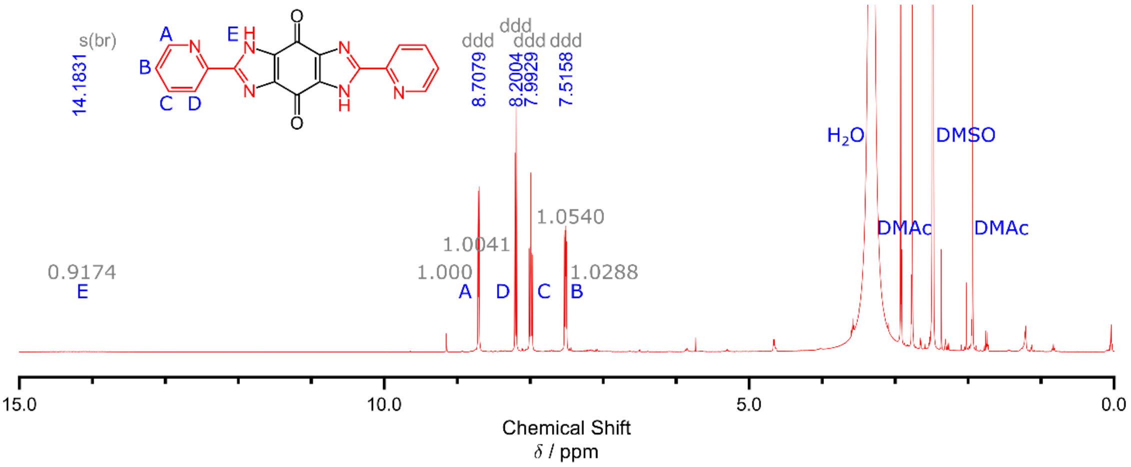

*Figure S 56: ^{1}H NMR of **5** (400.13 MHz, 298 K) in deuterated dimethyl sulphoxide (DMSO).*

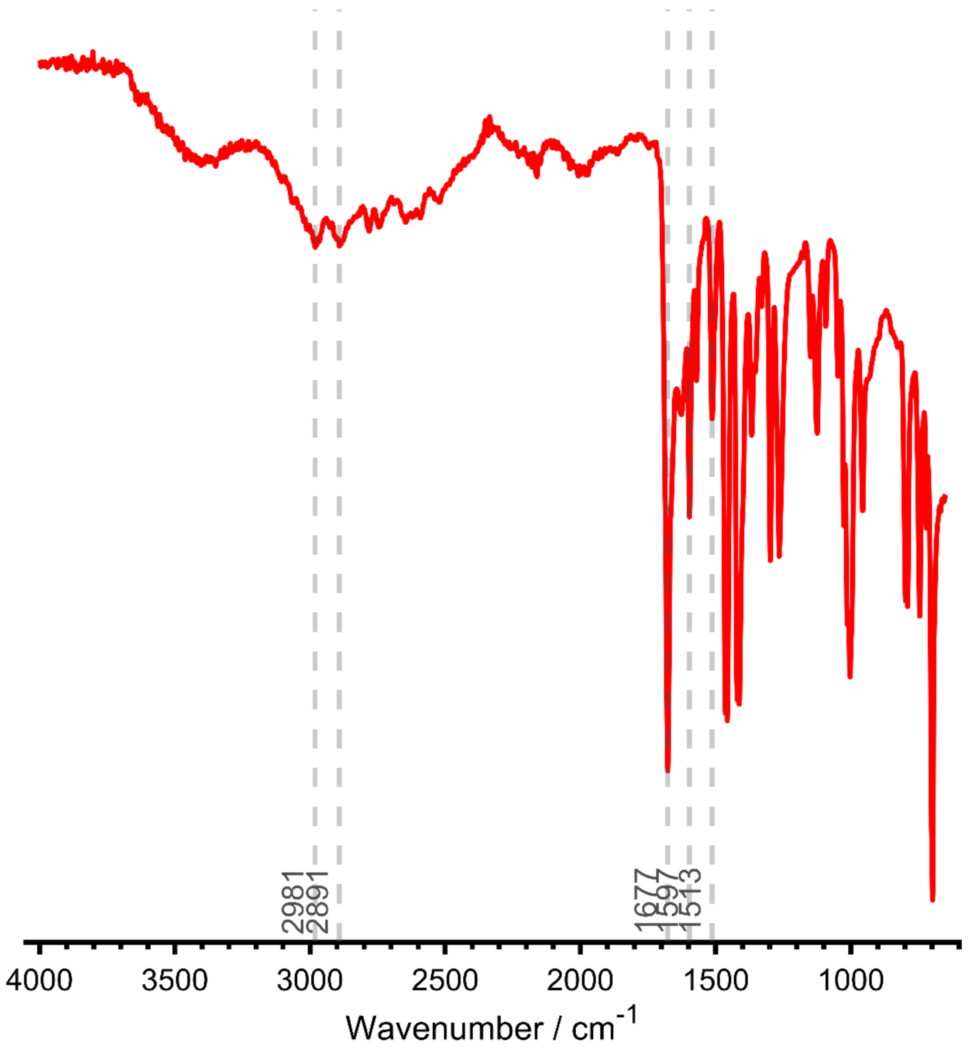

Figure S 57: ATR-IR spectrum of ***5****.*

2,6-di(pyridin-3-yl)benzo[1,2-d:4,5-d']diimidazole-4,8(1H,5H)-dione (**6**)

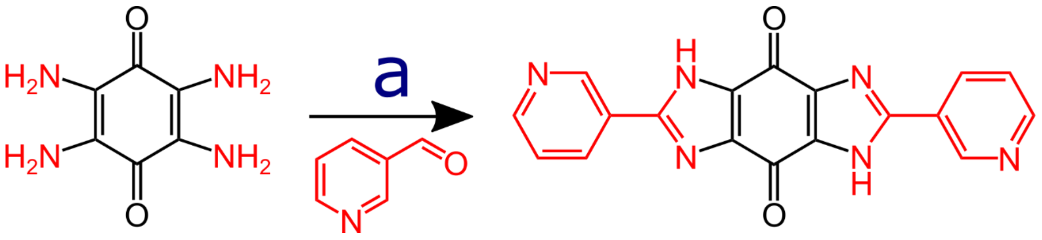

*Scheme S 6: Synthetic scheme of the synthesis of **6** (a) 3-pyridinecarboxaldehyde, N, N-dimethylacetamide, Δ.*

1 (101 mg, 0.60 mmol) and 3-pyridinecarboxaldehyde (0.12 ml, 1.28 mmol) were added to N, N-dimethylacetamide (4 ml). The solution was heated (100 °C, 3 d) in the dark under an inert atmosphere. Methanol (20 ml) was then added to form a brown suspension. The suspension was filtered to yield a brown powder as the product (89 mg, 0.26 mmol, 43% yield).

Elemental Analysis / %: Predicted: C 63.16, H 2.94, N 24.55; Observed: C 60.3, H 3.2, N 23.1.

^{1}H NMR (400.13 MHz, 298 K, Figure S 58, d_6-DMSO): δ 14.46 (E, 2H, s (br)), 9.31 (D, 2H, d, 1.96 Hz), 8.66 (A, 2H, dd, 4.75 Hz, 1.10 Hz), 8.47 (C, 2H, ddd, 8.00 Hz, 1.96 Hz, 1.10 Hz), 7.56 ppm (B, 2H, dd, 8.00 Hz, 4.75 Hz).

^{13}C NMR (100.59 MHz, 298 K, d_6-DMSO): δ 147.8, 134.2, 124.5 ppm. Several peaks missing.

MS (ES^{+}) = Calculated: 343.0943 Da; Observed: 343.0943 Da (0 ppm).

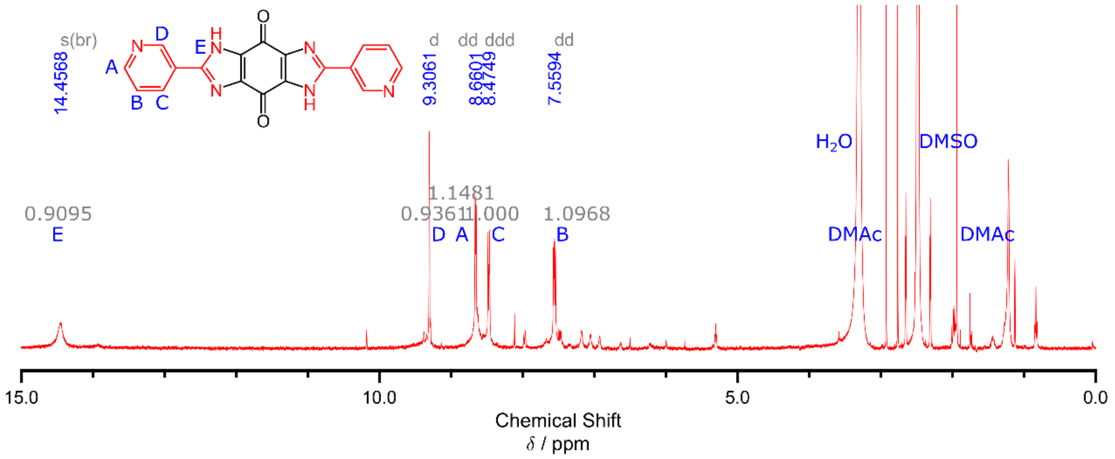

Figure S 58: ^{1}H NMR spectrum of ***6*** *(400.13 MHz, 298 K) in deuterated dimethyl sulphoxide (DMSO).*

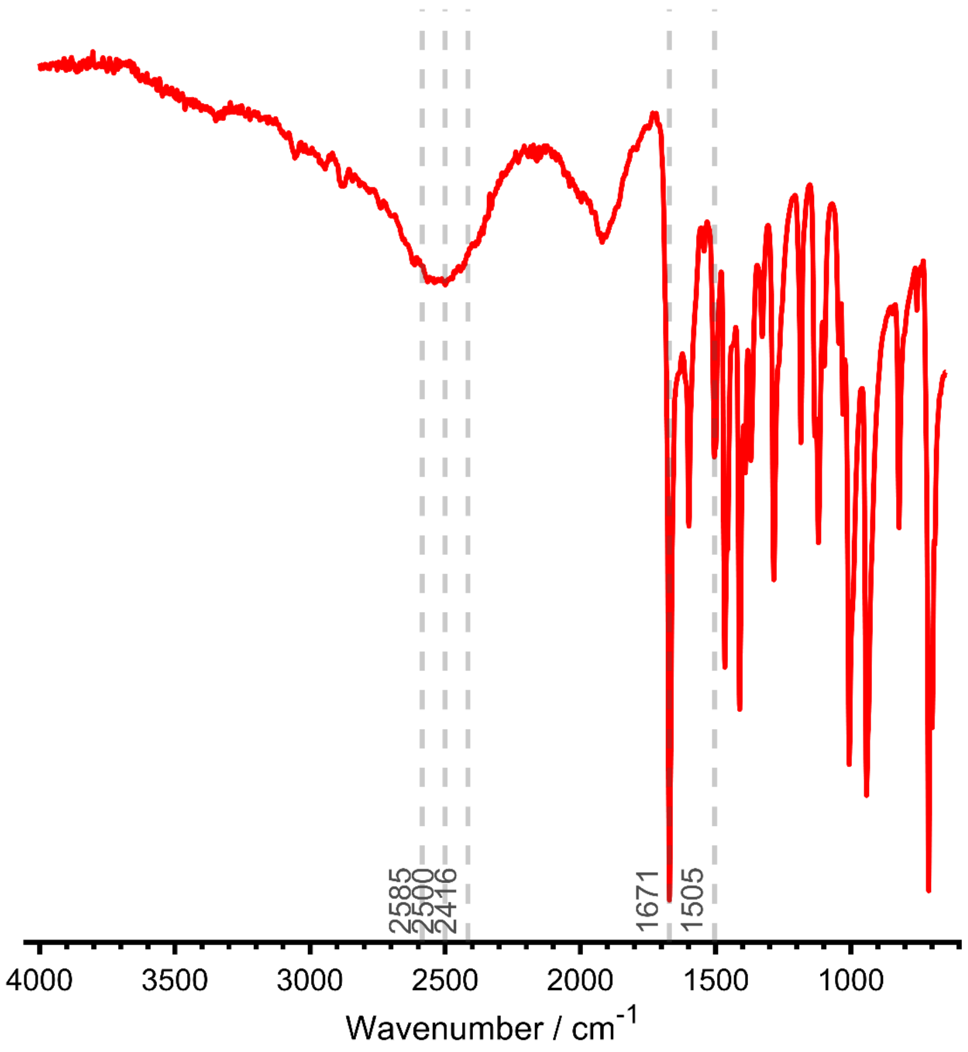

*Figure S 59: ATR-IR spectrum of **6**.*

2,2'-(4,8-dioxo-1,4,5,8-tetrahydrobenzo[1,2-d:4,5-d']diimidazole-2,6-diyl)bis(1-methylpyridin-1-ium) diiodide (**7**)

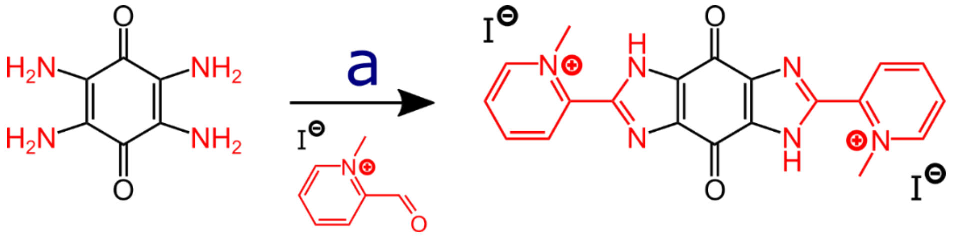

*Scheme S 7: Synthetic scheme of the synthesis of **7** (a) 2-formyl-1-methylpyridin-1-ium iodide, N, N-dimethylacetamide, Δ.*

2-Pyridinecarboxaldehye (5.4 ml, 56.77 mmol) was added dropwise to a solution of iodomethane (7 ml, 112.4 mmol) in dichloromethane (20 ml, dry, degassed) under an inert atmosphere. The solution was stirred in the dark for 5 d after which the orange suspension was filtered under vacuum and washed with ether. A yellow powder corresponding to 2-formyl-1-methylpyridin-1-ium iodide was obtained (875 mg, 3.51 mmol, 6% yield).

1 (97 mg, 0.58 mmol) and 2-formyl-1-methylpyridin-1-ium iodide (303 mg, 1.22 mmol) were added to N, N-dimethylacetamide (4 ml). The solution was heated (100 °C, 4 d) under inert atmosphere and in the dark. The solution was cooled down to ambient conditions and methanol (15 ml) was added to form a brown precipitate. The suspension was filtered under vacuum to give a brown powder (217 mg, 0.35 mmol, 60% yield).

Elemental Analysis / %: Predicted: C 38.36, H 2.58, N 13.42; Observed: C 38.2, H 3.2, N 12.9.

^{1}H NMR (298 K, Figure S 60, d_6-DMSO): 2-formyl-1-methylpyridin-1-ium iodide (400.03 MHz): δ 10.28 (E, 1H, s), 9.14 (A, 1H, d, 5.75 Hz), 8.80 (C, 1H, dd, 7.77 Hz, 6.85 Hz), 8.53 (D, 1H, d, 7.77 Hz), 8.35 (B, 1H, dd, 6.85 Hz, 5.75 Hz), 4.65 ppm (F, 3H, s); **7** (400.13 MHz): 10.26 (E, 2H, s), 9.28 (A, 2H, d, 8.31 Hz), 8.95 (D, 2H, d, 6.03 Hz), 8.52 (C, 2H, dd, 7.91 Hz, 6.03 Hz), 8.02 (B, 2H, dd, 8.31 Hz, 7.91 Hz), 4.45 ppm (F, 6H, s). *Note: additional unresolved coupling observed.*

^{13}C NMR (298 K, d_6-DMSO): 2-formyl-1-methylpyridin-1-ium iodide (100.59 MHz): δ 184.1 (HC=O), 147.1 (C alpha to N^+), 131.1 (C para to N^+), 128.2 (C meta to N^+), 46.4 ppm ($-CH_3$). Two peaks missing; **7** (100.61 MHz): δ 171.0 (C=O), 155.7 (C-N), 151.1 ($C_{Imidazole}$), 146.4 (C-Imidazole), 144.3 (C alpha to N^+), 142.9 (C meta to N^+, ortho to imidazole), 125.8 (C para to N^+), 116.2 (C meta to N^+, para to imidazole), 45.8 ppm ($-CH_3$).

MS (ES^+) = 343.1105 Da (Loss of methyl groups from pyridiniums under ionisation).

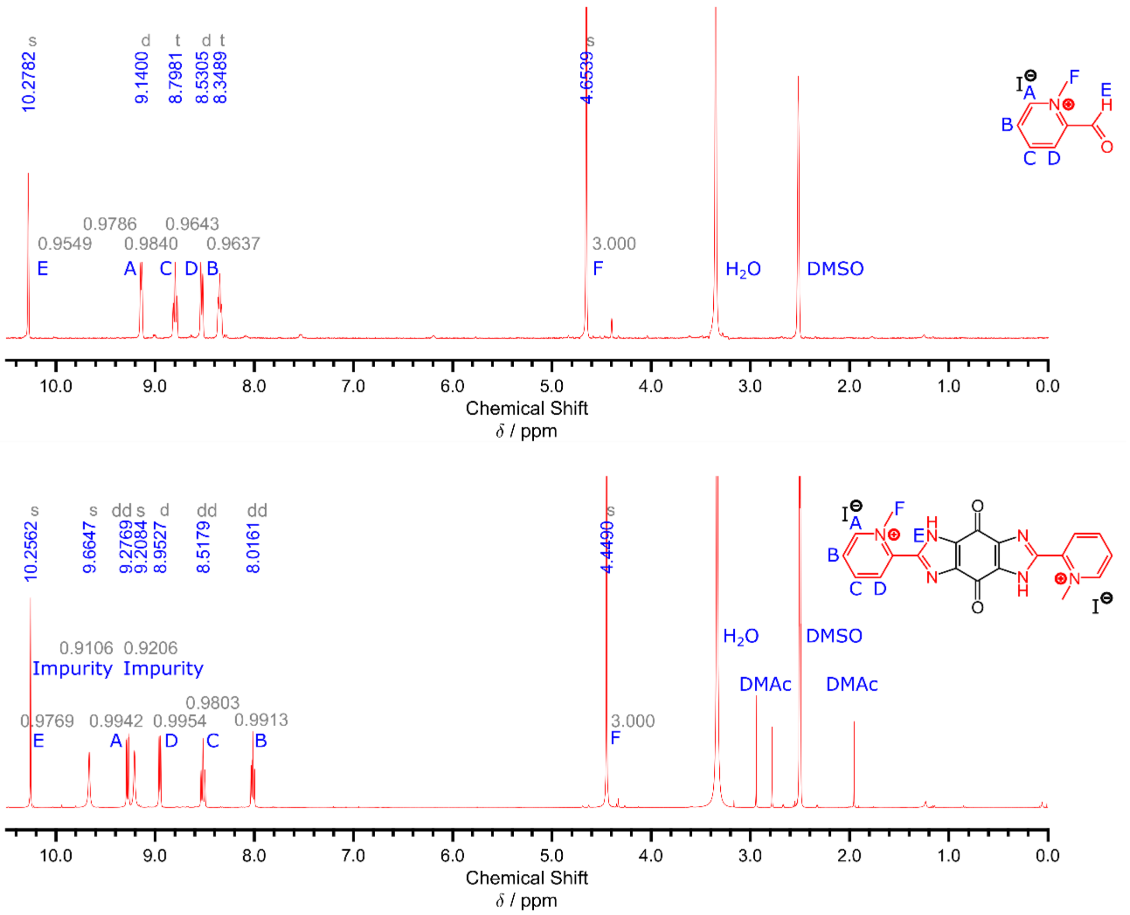

Figure S 60: ^{1}H NMR spectra of (above) 2-formyl-1-methylpyridin-1-ium iodide (400.03 MHz, 298 K) and (below) ***7*** *(400.13 MHz, 298 K) in deuterated dimethyl sulphoxide (DMSO).*

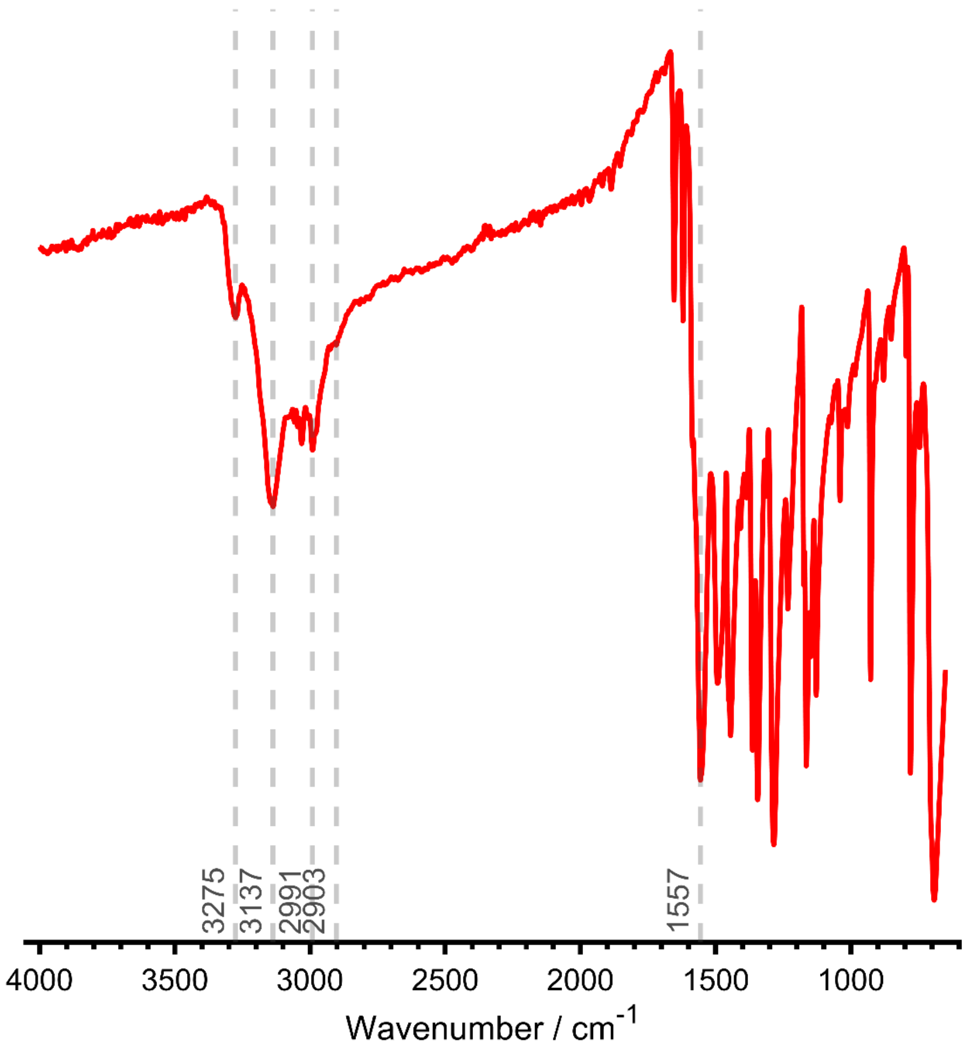

*Figure S 61: ATR-IR spectrum of **7**.*

4,4'-(4,8-dioxo-1,4,5,8-tetrahydrobenzo[1,2-d:4,5-d']diimidazole-2,6-diyl)bis(1-methylpyridin-1-ium) diiodide (**8**)

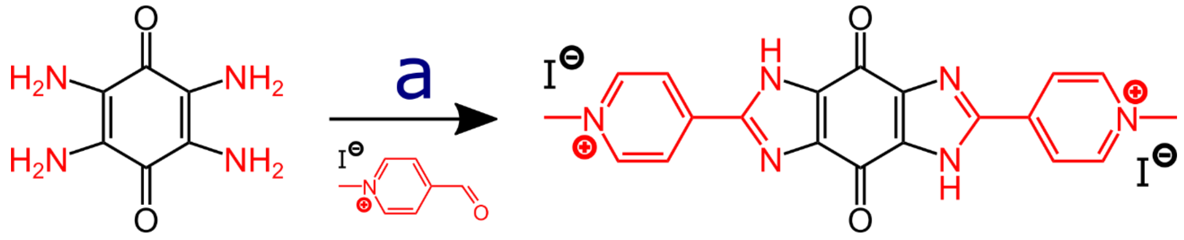

*Scheme S 8: Synthetic scheme of the synthesis of **8** (a) 4-formyl-1-methylpyridin-1-ium iodide, N, N-dimethylacetamide, Δ.*

The protocols for the condensation of **1** with an aldehyde described by Manivannan *et al.* were initially attempted.[32,37] However, the protocol described by Karayannidis *et al.* was found to be more successful.[39] 4-formyl-1-methylpyridin-1-ium iodide was synthesised as described previously.[40]

1 (110 mg, 0.65 mmol) and 4-formyl-1-methylpyridin-1-ium iodide (335 mg, 1.35 mmol) were added to N, N-dimethylacetamide (4 ml). The solution was heated (100 °C, 5 d) under inert atmosphere and in the dark. The solution was cooled down to ambient conditions and methanol (15 ml) was added to form a brown precipitate. The suspension was filtered under vacuum to give a brown powder (348 mg, 0.56 mmol, 85% yield).

Elemental Analysis / %: Predicted: C 38.36, H 2.58, N 13.42; Observed: C 38.1, H 3.2, N 12.9.

^{1}H NMR (298 K, Figure S 62, d_6-DMSO): δ 4-formyl-1-methylpyridin-1-ium iodide (400.03 MHz): 10.24 (D, 1H, s), 9.25 (B, 2H, d, 4.46 Hz), 8.49 (C, 2H, d, 4.46 Hz), 4.45 ppm (A, 3H, s); **8** (400.13 MHz): δ 9.93 (D, 2H, s), 8.93 (B, 4H, d, 6.74 Hz), 8.68 (C, 4H, d, 6.74 Hz), 4.30 ppm (A, 6H, s).

^{13}C NMR (100.61 MHz, 298 K, d_6-DMSO): δ 171.04 (C=O), 155.36 (C-N), 152.39 (C-NH), 148.28 ($C_{Imidazole}$), 145.75 (C-Imidazole), 125.01 (ArH ortho to N^+), 115.85 (ArH meta to N^+), 47.68 ppm ($-CH_3$).

MS (ES^+) = 343.1311 Da (Loss of methyl groups from pyridiniums under ionisation).

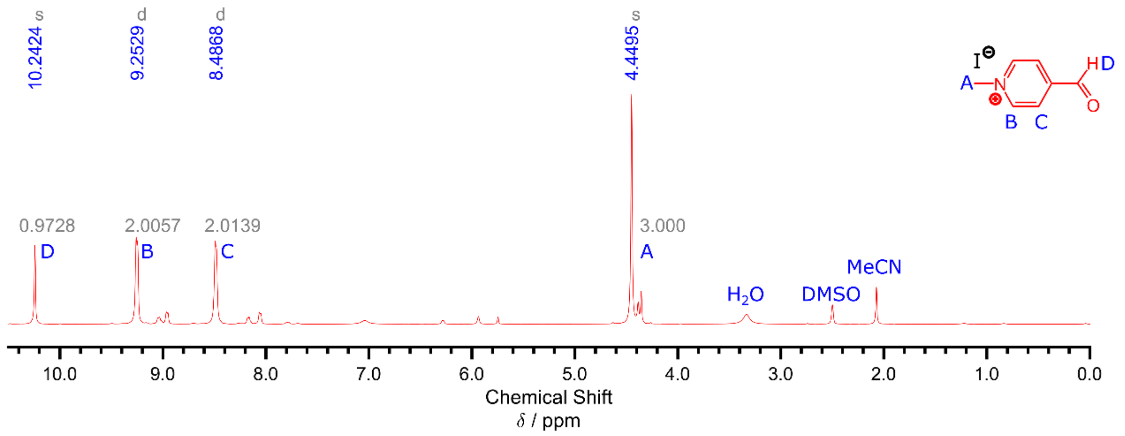

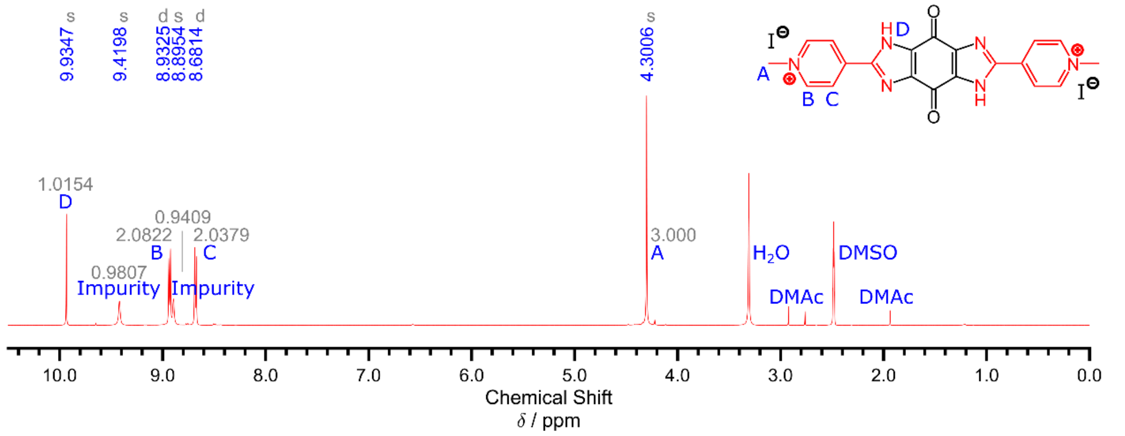

*Figure S 62: ^{1}H NMR spectra of (above) 4-formyl-1-methylpyridin-1-ium iodide (400.03 MHz, 298 K), (below) **8** (400.13 MHz, 298 K) in deuterated dimethyl sulphoxide (DMSO).*

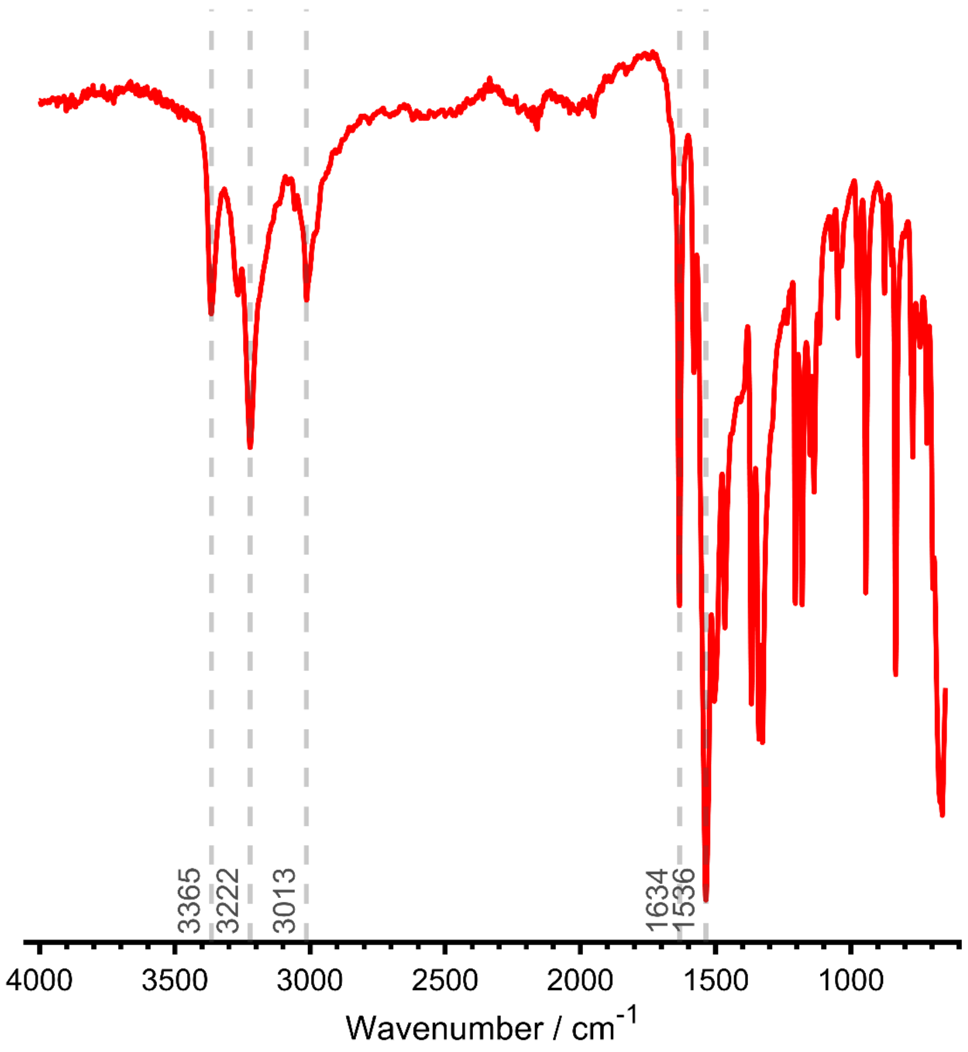

*Figure S 63: ATR-IR spectrum of **8**.*

3,3'-(4,8-dioxo-1,4,5,8-tetrahydrobenzo[1,2-d:4,5-d']diimidazole-2,6-diyl)bis(1-methylpyridin-1-ium) diiodide (**9**)

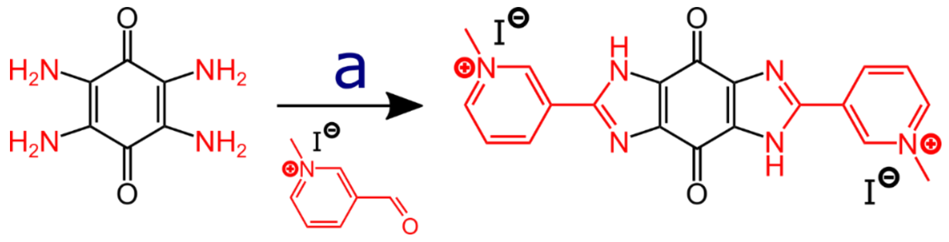

*Scheme S 9: Synthetic scheme of the synthesis of **9** (a) 3-formyl-1-methylpyridin-1-ium iodide, N, N-dimethylacetamide, Δ.*

3-Pyridinecarboxaldehye (5.4 ml, 57.47 mmol) was added dropwise to a solution of iodomethane (7 ml, 112.4 mmol) in dichloromethane (20 ml, dry, degassed) under inert atmosphere. The solution was stirred in the dark for 7 d after which the yellow suspension was filtered under vacuum and washed with hexane then ether. A yellow powder corresponding to 3-formyl-1-methylpyridin-1-ium iodide was obtained (13.90 g, 55.81 mmol, 97% yield).

1 (98 mg, 0.58 mmol) and 3-formyl-1-methylpyridin-1-ium iodide (302 mg, 1.21 mmol) were added to N, N-dimethylacetamide (4 ml). The solution was heated (100 °C, 2 d) under inert atmosphere and in the dark. The solution was cooled down to ambient conditions and methanol (15 ml) was added to form a brown precipitate. The suspension was filtered under vacuum to give a brown powder (206 mg, 0.33 mmol, 57% yield).

Elemental Analysis / %: Predicted: C 38.36, H 2.58, N 13.42; Observed: C 37.7, H 3.2, N 12.8.

^{1}H NMR (298 K, Figure S 64, d_6-DMSO): 3-formyl-1-methylpyridin-1-ium iodide (400.03 MHz): δ 10.20 (F, 1H, s), 9.55 (E, 1H, s), 9.22 (B, 1H, d, 5.78 Hz), 9.01 (D, 1H, d, 7.80 Hz), 8.35 (C, 1H, dd, 7.80 Hz, 5.78 Hz), 4.46 ppm (A, 3H, s); **9** (400.03 MHz): δ 9.94 (F, 2H, s), 9.68 (E, 2H, s), 9.13 (B, 2H, d, 7.88 Hz), 8.95 (D, 2H, d, 5.76 Hz), 8.18 (C, 2H, dd, 7.88 Hz, 5.76 Hz), 4.42 ppm (A, 6H, s)

^{13}C NMR (298 K, d_6-DMSO): 3-formyl-1-methylpyridin-1-ium iodide (100.59 MHz): δ 189.5 (HC=O), 149.5 (C, alpha to N^+, ortho to aldehyde), 147.2 (C meta to N^+, alpha to aldehyde), 145.2 (C alpha to N^+), 134.5 (C para to N^+), 128.6 (C meta to N^+), 48.9 ppm (-CH_3); **9** (100.59 MHz): δ 171.5 (C=O), 154.2 (C-N), 148.0 (C-NH), 145.3 ($C_{Imidazole}$), 145.0 (C-Imidazole), 143.0 (C alpha to N^+, ortho to aldehyde), 138.2 (C alpha to N^+), 127.9 (C para to N^+), 114.9 (C meta to N^+), 48.6 ppm (-CH_3).

MS (ES^+) = 272.1150 Da (Fragmentation under ionisation).

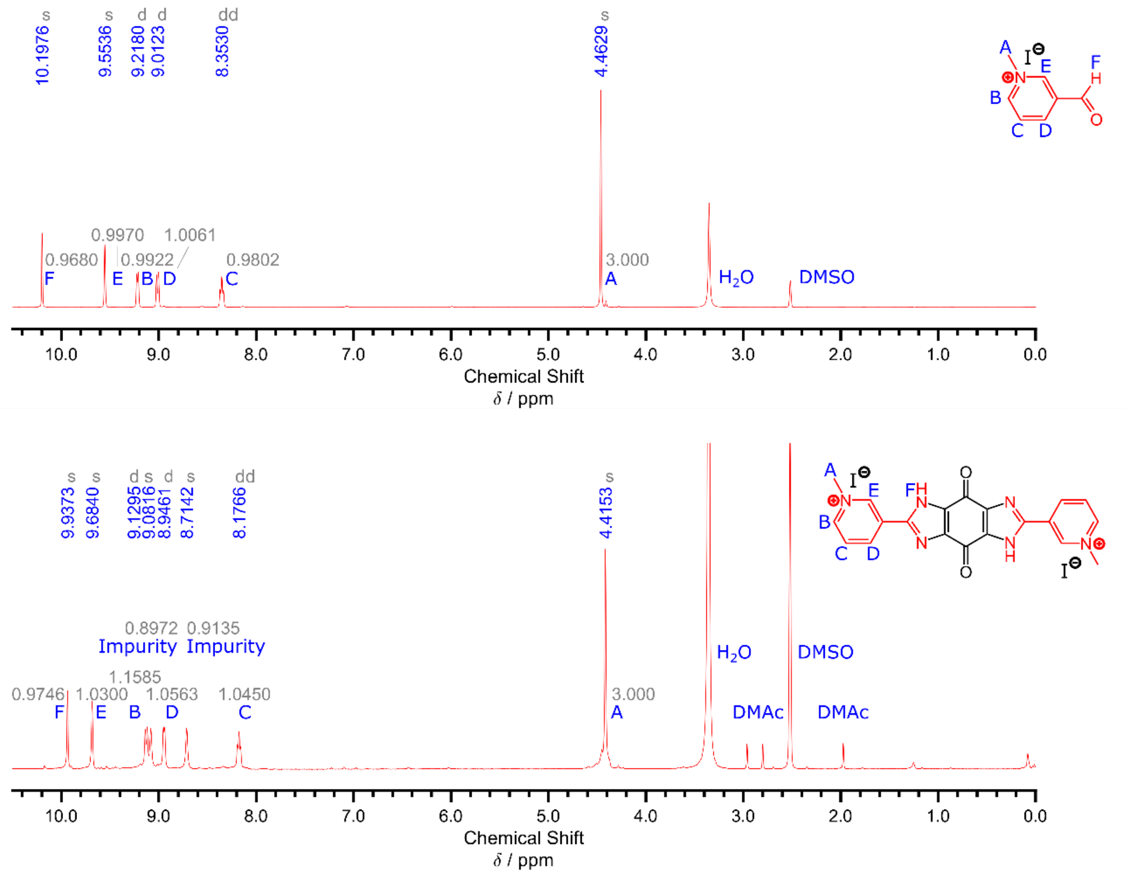

Figure S 64: ^{1}H NMR spectra of (above) 3-formyl-1-methylpyridin-1-ium iodide (400.03 MHz, 298 K) and (below) ***9*** *(400.03 MHz, 298 K) in deuterated dimethyl sulphoxide (DMSO).*

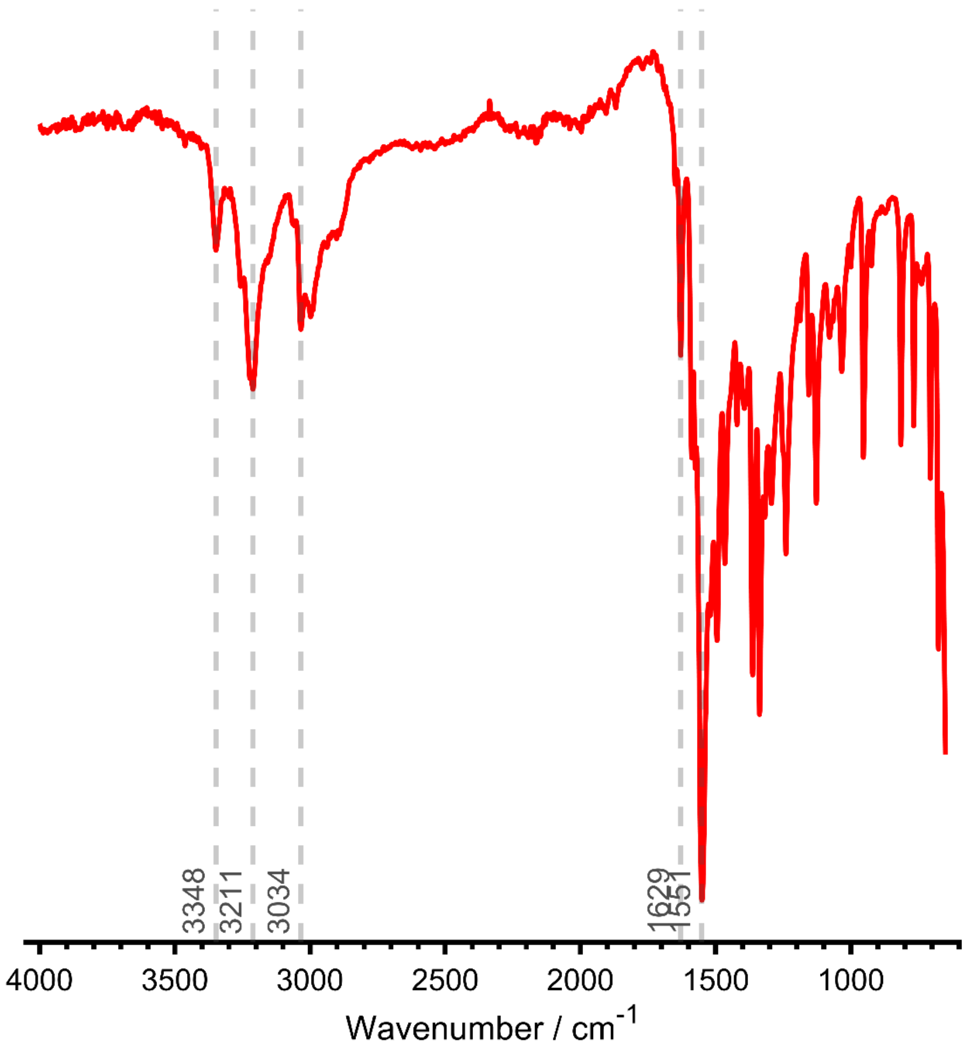

Figure S 65: ATR-IR spectrum of ***9****.*

DFT Coordinates

2,3,5,6-tetraaminocyclohexa-2,5-diene-1,4-dione (**1**)

Coordinates (B3LYP/TZVP, PCM (Water))

Oxidised

*Table S 16: **1** Oxidised - Coordinates (B3LYP/TZVP, PCM (Water))*

Element	x	y	z
**O**	-0.000027	2.653889	0.039882
**N**	-2.436730	1.437525	0.036283
**N**	2.436692	1.437575	0.036279
**C**	-1.276866	0.679317	-0.006181
**C**	-0.000017	1.421056	0.025967
**C**	1.276851	0.679340	-0.006182
**H**	2.279592	2.419880	-0.146238
**H**	3.220924	1.070150	-0.486002
**H**	-2.279645	2.419834	-0.146226
**H**	-3.220937	1.070095	-0.486034
**O**	0.000027	-2.653889	-0.039879
**N**	2.436731	-1.437524	-0.036281
**N**	-2.436693	-1.437574	-0.036278
**C**	1.276866	-0.679317	0.006179
**C**	0.000017	-1.421056	-0.025973
**C**	-1.276851	-0.679340	0.006180
**H**	-2.279591	-2.419880	0.146236
**H**	-3.220921	-1.070152	0.486012
**H**	2.279644	-2.419835	0.146224
**H**	3.220933	-1.070097	0.486044

Reduced

*Table S 17: **1** Reduced - Coordinates (B3LYP/TZVP, PCM (Water))*

Element	x	y	z
**O**	0.499106	2.731016	-0.014357
**N**	2.638660	0.990989	-0.031002
**N**	-2.143915	1.818074	-0.048545
**C**	1.324935	0.498147	0.017162
**C**	0.234174	1.367542	-0.017106
**C**	-1.077884	0.893907	0.004925
**H**	-2.093885	2.520882	0.680667
**H**	-3.028089	1.325626	0.016572
**H**	2.696100	1.979024	0.172268
**H**	3.286463	0.467455	0.544715

**O**	-0.370419	-2.728340	-0.002346
**N**	-2.663392	-0.959123	0.009309
**N**	2.205076	-1.774422	0.057581
**C**	-1.308749	-0.489572	-0.002902
**C**	-0.211470	-1.358930	0.005238
**C**	1.101367	-0.887783	0.006362
**H**	1.921625	-2.734695	-0.085180
**H**	2.923469	-1.537500	-0.618097
**H**	-2.905983	-1.510500	0.823491
**H**	-2.946887	-1.443795	-0.833706
**H**	-0.199093	3.181412	-0.509807
**H**	-1.312457	-2.937817	-0.050781

4,8-dioxo-4,8-dihydrobenzo[1,2-d:4,5-d']bis([1,2,3]triazole)-1,5-diide (**2**)

Coordinates (B3LYP/TZVP, PCM (Water))

H

Oxidised

Table S 18: $\mathbf{2_H}$ Oxidised - Coordinates (B3LYP/TZVP, PCM (Water))

Element	x	y	z
**O**	0.000000	2.767477	0.000001
**N**	2.476818	1.128166	0.000002
**N**	3.166229	0.000000	-0.000007
**N**	2.476818	-1.128166	0.000001
**C**	1.213994	0.706357	0.000001
**C**	1.213994	-0.706357	0.000003
**C**	0.000000	1.554403	0.000004
**O**	0.000000	-2.767477	0.000002
**N**	-2.476818	-1.128166	-0.000006
**N**	-3.166229	0.000000	0.000000
**N**	-2.476818	1.128166	-0.000004
**C**	-1.213994	-0.706357	0.000002
**C**	-1.213994	0.706357	0.000000
**C**	0.000000	-1.554403	0.000007
**H**	-4.178511	0.000000	-0.000001
**H**	4.178511	0.000000	-0.000011

Reduced

*Table S 19: **2**$_{H}$ Reduced - Coordinates (B3LYP/TZVP, PCM (Water))*

Element	x	y	z
**O**	0.041981	2.843342	-0.000020
**N**	2.486421	1.069661	-0.000008
**N**	3.129149	-0.076963	-0.000008
**N**	2.440975	-1.196923	0.000011
**C**	1.183247	0.682646	-0.000007
**C**	1.152393	-0.759762	0.000005
**C**	0.040060	1.483743	-0.000013
**O**	-0.041981	-2.843342	0.000025
**N**	-2.486421	-1.069661	0.000012
**N**	-3.129149	0.076963	-0.000002
**N**	-2.440975	1.196923	-0.000007
**C**	-1.183247	-0.682646	0.000005
**C**	-1.152393	0.759762	-0.000007
**C**	-0.040060	-1.483743	0.000013
**H**	-4.141354	0.097499	-0.000002
**H**	4.141354	-0.097499	-0.000011
**H**	-0.953305	-3.166943	0.000037
**H**	0.953305	3.166943	-0.000030

Li

Oxidised

*Table S 20: **2**$_{Li}$ Oxidised - Coordinates (B3LYP/TZVP, PCM (Water))*

Element	x	y	z
**O**	0.940102	2.593415	-0.000037
**N**	-2.050869	1.831001	-0.000022
**N**	-3.090662	0.998850	-0.000017
**N**	-2.678168	-0.281544	0.000003
**C**	-0.935371	1.072978	-0.000010
**C**	-1.340477	-0.262968	0.000006
**C**	0.477033	1.451836	-0.000007
**O**	-0.940102	-2.593415	0.000034
**N**	2.050869	-1.831001	0.000024
**N**	3.090662	-0.998850	0.000002
**N**	2.678168	0.281544	-0.000007
**C**	0.935371	-1.072978	0.000015
**C**	1.340477	0.262968	-0.000001
**C**	-0.477033	-1.451836	0.000022
**Li**	3.332626	2.286920	-0.000025

**Li**	-3.332623	-2.286922	0.000022

Reduced

*Table S 21: **2**$_{Li}$ Reduced - Coordinates (B3LYP/TZVP, PCM (Water))*

Element	x	y	z
**O**	0.878867	2.699198	0.002917
**N**	-2.027068	1.816096	0.004251
**N**	-3.069327	1.003159	0.001868
**N**	-2.714411	-0.289474	0.002013
**C**	-0.904681	1.026649	0.005589
**C**	-1.342849	-0.329904	0.004696
**C**	0.441679	1.391626	0.005024
**O**	-0.878873	-2.699194	0.002776
**N**	2.027060	-1.816091	0.004422
**N**	3.069325	-1.003154	0.001902
**N**	2.714407	0.289477	0.001912
**C**	0.904677	-1.026644	0.005874
**C**	1.342846	0.329907	0.004498
**C**	-0.441686	-1.391621	0.004742
**Li**	4.080719	1.775612	-0.057696
**Li**	-4.080643	-1.775684	-0.057457
**H**	0.101812	3.274350	0.001497
**H**	-0.101817	-3.274345	0.001307

Na

Oxidised

*Table S 22: **2**$_{Na}$ Oxidised - Coordinates (B3LYP/TZVP, PCM (Water))*

Element	x	y	z
**O**	0.742344	2.661192	-0.042231
**N**	-2.107102	1.739535	-0.038668
**N**	-3.122072	0.878911	-0.033168
**N**	-2.694069	-0.395660	-0.032522
**C**	-0.982576	1.002371	-0.042670
**C**	-1.354364	-0.349787	-0.039244
**C**	0.409756	1.481363	-0.042105
**O**	-0.742343	-2.661190	-0.042403
**N**	2.107103	-1.739533	-0.038732
**N**	3.122072	-0.878909	-0.033156
**N**	2.694069	0.395662	-0.032440
**C**	0.982576	-1.002369	-0.042712

**C**	1.354364	0.349789	-0.039193
**C**	-0.409756	-1.481360	-0.042202
**Na**	5.181406	0.368885	0.164888
**Na**	-5.181408	-0.368895	0.164804

Reduced

*Table S 23: **2**$_{Na}$ Reduced - Coordinates (B3LYP/TZVP, PCM (Water))*

Element	x	y	z
**O**	-0.908277	-2.691147	-0.025517
**N**	1.991144	-1.853363	-0.026614
**N**	3.043899	-1.051822	-0.023893
**N**	2.720161	0.247768	-0.021828
**C**	0.887457	-1.039994	-0.027004
**C**	1.352175	0.311573	-0.024813
**C**	-0.464078	-1.386126	-0.026042
**O**	0.908278	2.691121	-0.025405
**N**	-1.991145	1.853337	-0.026614
**N**	-3.043899	1.051794	-0.023939
**N**	-2.720160	-0.247796	-0.021911
**C**	-0.887457	1.039969	-0.026998
**C**	-1.352174	-0.311598	-0.024859
**C**	0.464079	1.386101	-0.025983
**Na**	-5.079647	-0.405999	0.109290
**Na**	5.079645	0.406113	0.109303
**H**	-0.132226	-3.267784	-0.024734
**H**	0.132227	3.267759	-0.024620

K

Oxidised

*Table S 24: **2**$_{K}$ Oxidised - Coordinates (B3LYP/TZVP, PCM (Water))*

Element	x	y	z
**O**	0.968491	2.588180	0.027579
**N**	-1.951399	1.912021	0.024123
**N**	-3.039775	1.140802	0.022281
**N**	-2.719080	-0.163779	0.023537
**C**	-0.892445	1.082950	0.026520
**C**	-1.379541	-0.231898	0.026222
**C**	0.535788	1.439927	0.027047
**O**	-0.968477	-2.588202	0.027785
**N**	1.951412	-1.912044	0.023812

**N**	3.039788	-1.140825	0.021771
**N**	2.719094	0.163756	0.023060
**C**	0.892458	-1.082972	0.026380
**C**	1.379555	0.231876	0.025976
**C**	-0.535775	-1.439949	0.027162
**K**	5.592222	0.249183	-0.062905
**K**	-5.592255	-0.249127	-0.061771

Reduced

*Table S 25: **2**$_K$ Reduced - Coordinates (B3LYP/TZVP, PCM (Water))*

Element	x	y	z
**O**	-0.992734	2.661739	-0.026569
**N**	1.931456	1.915259	-0.025112
**N**	3.012040	1.144992	-0.023419
**N**	2.729647	-0.162235	-0.024549
**C**	0.853865	1.068144	-0.027207
**C**	1.363318	-0.267184	-0.026936
**C**	-0.508520	1.369057	-0.027329
**O**	0.992739	-2.661736	-0.026580
**N**	-1.931450	-1.915255	-0.025102
**N**	-3.012036	-1.144989	-0.023407
**N**	-2.729641	0.162237	-0.024543
**C**	-0.853860	-1.068140	-0.027191
**C**	-1.363313	0.267187	-0.026944
**C**	0.508525	-1.369053	-0.027345
**K**	-5.549926	0.228987	0.065193
**K**	5.549913	-0.228995	0.065200
**H**	-0.233015	3.259674	-0.025819
**H**	0.233020	-3.259670	-0.025825

TMA

Oxidised

*Table S 26: **2**$_{TMA}$ Oxidised - Coordinates (B3LYP/TZVP, PCM (Water))*

Element	x	y	z
**O**	-2.132886	-1.706952	0.412690
**N**	0.697262	-2.642062	0.015016
**N**	2.013559	-2.543492	-0.206538
**N**	2.390157	-1.263560	-0.334596
**C**	0.211114	-1.387920	0.032808
**C**	1.283690	-0.514584	-0.191320

**C**	-1.179383	-0.952757	0.234088
**O**	2.132886	1.706952	-0.412690
**N**	-0.697262	2.642062	-0.015016
**N**	-2.013559	2.543492	0.206538
**N**	-2.390157	1.263560	0.334596
**C**	-0.211114	1.387920	-0.032808
**C**	-1.283690	0.514584	0.191320
**C**	1.179383	0.952757	-0.234088
**N**	-5.761278	-0.155204	-0.009637
**N**	5.761278	0.155204	0.009637
**C**	-5.179617	-0.628362	1.296396
**C**	-4.912755	-0.668114	-1.142261
**C**	-7.164622	-0.667145	-0.157268
**C**	-5.762194	1.350479	-0.032700
**C**	5.762194	-1.350479	0.032700
**C**	7.164622	0.667145	0.157268
**C**	4.912755	0.668114	1.142261
**C**	5.179617	0.628362	-1.296396
**H**	-4.734886	1.685794	0.083227
**H**	-6.173755	1.682779	-0.982482
**H**	-6.380368	1.708335	0.786949
**H**	-7.572266	-0.308810	-1.099055
**H**	-7.144024	-1.753828	-0.149306
**H**	-7.762396	-0.299074	0.672442
**H**	-4.156137	-0.270991	1.357128
**H**	-5.784993	-0.230831	2.107126
**H**	-5.198404	-1.714697	1.309326
**H**	-3.903540	-0.294404	-1.003308
**H**	-4.916172	-1.754245	-1.112535
**H**	-5.335581	-0.314356	-2.079109
**H**	6.173755	-1.682779	0.982482
**H**	4.734886	-1.685794	-0.083227
**H**	6.380368	-1.708335	-0.786949
**H**	7.144024	1.753828	0.149306
**H**	7.572266	0.308810	1.099055
**H**	7.762396	0.299074	-0.672442
**H**	4.916172	1.754245	1.112535
**H**	3.903540	0.294404	1.003308
**H**	5.335581	0.314356	2.079109
**H**	5.784993	0.230831	-2.107126
**H**	4.156137	0.270991	-1.357128
**H**	5.198404	1.714697	-1.309326

Reduced

*Table S 27: **2_{TMA}** Reduced - Coordinates (B3LYP/TZVP, PCM (Water))*

Element	x	y	z
**O**	-0.738077	-2.578834	0.925401
**N**	0.586191	-0.451006	2.618727
**N**	1.132083	0.725288	2.933652
**N**	1.163990	1.579409	1.910606
**C**	0.230478	-0.372138	1.298125
**C**	0.595629	0.932180	0.841172
**C**	-0.368755	-1.328956	0.477891
**O**	0.738077	2.578834	-0.925401
**N**	-0.586191	0.451006	-2.618727
**N**	-1.132083	-0.725288	-2.933652
**N**	-1.163990	-1.579409	-1.910606
**C**	-0.230478	0.372138	-1.298125
**C**	-0.595629	-0.932180	-0.841172
**C**	0.368755	1.328956	-0.477891
**N**	-4.150690	0.440684	0.248838
**N**	4.150690	-0.440684	-0.248838
**C**	-3.086066	1.127969	1.060396
**C**	-4.021172	-1.050417	0.412507
**C**	-5.507421	0.884010	0.719510
**C**	-3.978294	0.800087	-1.202551
**C**	3.978294	-0.800087	1.202551
**C**	5.507421	-0.884010	-0.719510
**C**	4.021172	1.050417	-0.412507
**C**	3.086066	-1.127969	-1.060396
**H**	-2.991607	0.486047	-1.527588
**H**	-4.744140	0.289307	-1.780016
**H**	-4.084014	1.876558	-1.308478
**H**	-6.266028	0.388090	0.119832
**H**	-5.621598	0.613810	1.765920
**H**	-5.583883	1.961591	0.601266
**H**	-2.113702	0.805492	0.704286
**H**	-3.193938	2.202235	0.938090
**H**	-3.211665	0.854234	2.104388
**H**	-3.050719	-1.358523	0.035686
**H**	-4.112438	-1.293754	1.467714
**H**	-4.814450	-1.531468	-0.153607
**H**	4.744140	-0.289307	1.780016
**H**	2.991607	-0.486047	1.527588
**H**	4.084014	-1.876558	1.308478
**H**	5.621598	-0.613810	-1.765920
**H**	6.266028	-0.388090	-0.119832

**H**	5.583883	-1.961591	-0.601266
**H**	4.112438	1.293754	-1.467714
**H**	3.050719	1.358523	-0.035686
**H**	4.814450	1.531468	0.153607
**H**	3.193938	-2.202235	-0.938090
**H**	2.113702	-0.805492	-0.704286
**H**	3.211665	-0.854234	-2.104388
**H**	-0.512341	-2.634660	1.864025
**H**	0.512341	2.634660	-1.864025

TEA

Oxidised

*Table S 28: **2**$_{TEA}$ Oxidised - Coordinates (B3LYP/TZVP, PCM (Water))*

Family	x	y	z
**O**	-1.331611	-1.139617	-2.134347
**N**	0.604758	-2.633523	-0.395505
**N**	1.458114	-2.839047	0.616297
**N**	1.625401	-1.726009	1.346468
**C**	0.209448	-1.350608	-0.315579
**C**	0.855164	-0.775876	0.788198
**C**	-0.721741	-0.631193	-1.197546
**O**	1.331611	1.139617	2.134347
**N**	-0.604758	2.633523	0.395505
**N**	-1.458114	2.839047	-0.616297
**N**	-1.625401	1.726009	-1.346468
**C**	-0.209448	1.350608	0.315579
**C**	-0.855164	0.775876	-0.788198
**C**	0.721741	0.631193	1.197546
**N**	-4.810379	0.471795	0.397252
**N**	4.810379	-0.471795	-0.397252
**C**	-3.675903	0.607433	1.424489
**C**	-4.442844	-0.597065	-0.627699
**C**	-6.148830	0.181475	1.066039
**C**	-4.935663	1.811224	-0.344748
**C**	4.935663	-1.811224	0.344748
**C**	6.148830	-0.181475	-1.066039
**C**	4.442844	0.597065	0.627699
**C**	3.675903	-0.607433	-1.424489
**H**	-4.043645	1.882614	-0.963624
**H**	-5.803796	1.715691	-0.990433
**H**	-6.894900	0.256249	0.278770
**H**	-6.320541	1.001178	1.757982

**H**	-2.951506	1.276189	0.969919
**H**	-4.112307	1.115993	2.280376
**H**	-3.483040	-0.292183	-1.037814
**H**	-4.295139	-1.519893	-0.077542
**H**	5.803796	-1.715691	0.990433
**H**	4.043645	-1.882614	0.963624
**H**	6.894900	-0.256249	-0.278770
**H**	6.320541	-1.001178	-1.757982
**H**	4.295139	1.519893	0.077542
**H**	3.483040	0.292183	1.037814
**H**	4.112307	-1.115993	-2.280376
**H**	2.951506	-1.276189	-0.969919
**C**	-6.279597	-1.144684	1.793438
**C**	-5.072669	3.046850	0.529375
**C**	-2.962181	-0.662452	1.863390
**C**	-5.449473	-0.820410	-1.743331
**C**	2.962181	0.662452	-1.863390
**C**	5.449473	0.820410	1.743331
**C**	5.072669	-3.046850	-0.529375
**C**	6.279597	1.144684	-1.793438
**H**	-5.658340	-1.193411	2.684754
**H**	-7.318465	-1.230616	2.115159
**H**	-6.063451	-2.002179	1.157650
**H**	-5.190478	3.896671	-0.144584
**H**	-5.950097	3.020222	1.174022
**H**	-4.187918	3.233982	1.134634
**H**	-2.386440	-1.117339	1.060620
**H**	-2.252587	-0.365036	2.637168
**H**	-3.621308	-1.412497	2.291155
**H**	-5.587904	0.055754	-2.374000
**H**	-5.050863	-1.616335	-2.373816
**H**	-6.419663	-1.152293	-1.375143
**H**	5.950097	-3.020222	-1.174022
**H**	5.190478	-3.896671	0.144584
**H**	4.187918	-3.233982	-1.134634
**H**	5.050863	1.616335	2.373816
**H**	5.587904	-0.055754	2.374000
**H**	6.419663	1.152293	1.375143
**H**	2.252587	0.365036	-2.637168
**H**	2.386440	1.117339	-1.060620
**H**	3.621308	1.412497	-2.291155
**H**	7.318465	1.230616	-2.115159
**H**	5.658340	1.193411	-2.684754
**H**	6.063451	2.002179	-1.157650

Reduced

*Table S 29: **2**TEA Reduced - Coordinates (B3LYP/TZVP, PCM (Water))*

Element	x	y	z
**O**	0.398534	2.393390	1.470494
**N**	0.731301	-0.384386	2.590403
**N**	0.755268	-1.718837	2.624687
**N**	0.431966	-2.278751	1.458863
**C**	0.362862	-0.036794	1.317179
**C**	0.175616	-1.250714	0.586364
**C**	0.192350	1.231076	0.758763
**O**	-0.398534	-2.393390	-1.470494
**N**	-0.731301	0.384386	-2.590403
**N**	-0.755268	1.718837	-2.624687
**N**	-0.431966	2.278751	-1.458863
**C**	-0.362862	0.036794	-1.317179
**C**	-0.175616	1.250714	-0.586364
**C**	-0.192350	-1.231076	-0.758763
**N**	-4.171839	0.093280	-0.125686
**N**	4.171839	-0.093280	0.125686
**C**	-3.264185	-0.525662	0.946861
**C**	-4.028665	-0.698396	-1.421803
**C**	-5.621957	0.175673	0.335388
**C**	-3.690089	1.525966	-0.391174
**C**	3.690089	-1.525966	0.391174
**C**	5.621957	-0.175673	-0.335388
**C**	4.028665	0.698396	1.421803
**C**	3.264185	0.525662	-0.946861
**H**	-2.777513	1.429962	-0.970670
**H**	-4.448065	1.984420	-1.020243
**H**	-6.158252	0.685816	-0.460694
**H**	-5.620237	0.832677	1.200968
**H**	-2.279278	-0.102883	0.772562
**H**	-3.632202	-0.147935	1.897745
**H**	-2.958265	-0.753180	-1.609160
**H**	-4.389712	-1.700507	-1.216840
**H**	4.448065	-1.984420	1.020243
**H**	2.777513	-1.429962	0.970670
**H**	6.158252	-0.685816	0.460694
**H**	5.620237	-0.832677	-1.200968
**H**	4.389712	1.700507	1.216840
**H**	2.958265	0.753180	1.609160
**H**	3.632202	0.147935	-1.897745
**H**	2.279278	0.102883	-0.772562
**C**	-6.309869	-1.132006	0.683847

**C**	-3.438455	2.386414	0.835849
**C**	-3.138556	-2.041120	0.975849
**C**	-4.742540	-0.123516	-2.633099
**C**	3.138556	2.041120	-0.975849
**C**	4.742540	0.123516	2.633099
**C**	3.438455	-2.386414	-0.835849
**C**	6.309869	1.132006	-0.683847
**H**	-5.902065	-1.587468	1.583558
**H**	-7.357266	-0.899059	0.880972
**H**	-6.284695	-1.856798	-0.128801
**H**	-3.174400	3.380217	0.472232
**H**	-4.316098	2.493831	1.472084
**H**	-2.601716	2.029098	1.432526
**H**	-2.630602	-2.428037	0.095211
**H**	-2.503972	-2.277752	1.830830
**H**	-4.084334	-2.560057	1.103912
**H**	-4.341845	0.840895	-2.939294
**H**	-4.580654	-0.820416	-3.456607
**H**	-5.818888	-0.035344	-2.489092
**H**	4.316098	-2.493831	-1.472084
**H**	3.174400	-3.380217	-0.472232
**H**	2.601716	-2.029098	-1.432526
**H**	4.580654	0.820416	3.456607
**H**	4.341845	-0.840895	2.939294
**H**	5.818888	0.035344	2.489092
**H**	2.503972	2.277752	-1.830830
**H**	2.630602	2.428037	-0.095211
**H**	4.084334	2.560057	-1.103912
**H**	7.357266	0.899059	-0.880972
**H**	5.902065	1.587468	-1.583558
**H**	6.284695	1.856798	0.128801
**H**	0.609885	2.148051	2.381849
**H**	-0.609885	-2.148051	-2.381849

TPA

Oxidised

*Table S 30: **2**TPA Oxidised - Coordinates (B3LYP/TZVP, PCM (Water))*

Element	x	y	z
**O**	-0.850947	2.314452	1.241554
**N**	0.518097	0.064941	2.679114
**N**	1.050223	-1.153707	2.850267
**N**	1.097290	-1.831640	1.695802

**C**	0.217695	0.177185	1.374082
**C**	0.579907	-1.025867	0.750937
**C**	-0.436740	1.299108	0.686042
**O**	0.850947	-2.314452	-1.241554
**N**	-0.518097	-0.064941	-2.679114
**N**	-1.050223	1.153707	-2.850267
**N**	-1.097290	1.831640	-1.695802
**C**	-0.217695	-0.177185	-1.374082
**C**	-0.579907	1.025867	-0.750937
**C**	0.436740	-1.299108	-0.686042
**N**	-4.507474	0.669050	0.353639
**N**	4.507474	-0.669050	-0.353639
**C**	-3.714824	0.135260	1.582974
**C**	-4.110627	-0.085953	-0.910334
**C**	-6.006534	0.601414	0.599066
**C**	-4.123322	2.142676	0.156369
**C**	4.123322	-2.142676	-0.156369
**C**	6.006534	-0.601414	-0.599066
**C**	4.110627	0.085953	0.910334
**C**	3.714824	-0.135260	-1.582974
**H**	-3.059495	2.131297	-0.059910
**H**	-4.640650	2.474846	-0.739280
**H**	-6.471328	1.170656	-0.203111
**H**	-6.181749	1.139285	1.527558
**H**	-2.973448	0.900749	1.787266
**H**	-4.417035	0.125274	2.412989
**H**	-3.044737	0.083368	-1.036753
**H**	-4.259432	-1.140119	-0.705185
**H**	4.640650	-2.474846	0.739280
**H**	3.059495	-2.131297	0.059910
**H**	6.471328	-1.170656	0.203111
**H**	6.181749	-1.139285	-1.527558
**H**	4.259432	1.140119	0.705185
**H**	3.044737	-0.083368	1.036753
**H**	4.417035	-0.125274	-2.412989
**H**	2.973448	-0.900749	-1.787266
**C**	-6.621607	-0.784887	0.688195
**C**	-4.434568	3.092976	1.321777
**C**	-2.979774	-1.204405	1.497459
**C**	-4.848706	0.285258	-2.191161
**C**	2.979774	1.204405	-1.497459
**C**	4.848706	-0.285258	2.191161
**C**	4.434568	-3.092976	-1.321777
**C**	6.621607	0.784887	-0.688195

**H**	-6.195397	-1.333271	1.526641
**H**	-6.418181	-1.360299	-0.215891
**H**	-3.572342	3.756353	1.410229
**H**	-4.480302	2.556943	2.270637
**H**	-2.290900	-1.161340	2.345537
**H**	-2.336058	-1.234811	0.619739
**H**	-4.685876	1.331953	-2.447430
**H**	-5.923836	0.139961	-2.076591
**H**	3.572342	-3.756353	-1.410229
**H**	4.480302	-2.556943	-2.270637
**H**	4.685876	-1.331953	2.447430
**H**	5.923836	-0.139961	2.076591
**H**	2.290900	1.161340	-2.345537
**H**	2.336058	1.234811	-0.619739
**H**	6.195397	1.333271	-1.526641
**H**	6.418181	1.360299	0.215891
**C**	-5.695119	3.940960	1.132742
**C**	-3.792964	-2.491765	1.612862
**C**	-4.336401	-0.595848	-3.335243
**C**	-8.136280	-0.663885	0.879000
**H**	-8.376704	-0.110823	1.789663
**H**	-8.599790	-0.146623	0.036050
**H**	-8.588332	-1.653152	0.956970
**H**	-5.634065	4.521905	0.209828
**H**	-6.601413	3.336180	1.084681
**H**	-5.807867	4.641612	1.961735
**H**	-4.422599	-2.480681	2.504927
**H**	-4.434000	-2.673681	0.750345
**H**	-3.113988	-3.341816	1.696399
**H**	-3.265247	-0.452081	-3.486439
**H**	-4.515359	-1.653596	-3.128498
**H**	-4.849214	-0.343727	-4.264221
**C**	5.695119	-3.940960	-1.132742
**C**	3.792964	2.491765	-1.612862
**C**	4.336401	0.595848	3.335243
**C**	8.136280	0.663885	-0.879000
**H**	6.601413	-3.336180	-1.084681
**H**	5.634065	-4.521905	-0.209828
**H**	5.807867	-4.641612	-1.961735
**H**	4.515359	1.653596	3.128498
**H**	3.265247	0.452081	3.486439
**H**	4.849214	0.343727	4.264221
**H**	4.434000	2.673681	-0.750345
**H**	4.422599	2.480681	-2.504927

**H**	3.113988	3.341816	-1.696399
**H**	8.599790	0.146623	-0.036050
**H**	8.376704	0.110823	-1.789663
**H**	8.588332	1.653152	-0.956970

Reduced

*Table S 31: **2**$_{TPA}$ Reduced - Coordinates (B3LYP/TZVP, PCM (Water))*

Element	x	y	z
**O**	0.034622	-0.205817	2.828992
**N**	0.210273	-2.547637	0.928989
**N**	0.256028	-3.208086	-0.229761
**N**	0.185761	-2.407294	-1.292983
**C**	0.101268	-1.218659	0.613670
**C**	0.083139	-1.124902	-0.812708
**C**	0.018837	-0.108859	1.455439
**O**	-0.034622	0.205817	-2.828992
**N**	-0.210273	2.547637	-0.928989
**N**	-0.256028	3.208086	0.229761
**N**	-0.185761	2.407294	1.292983
**C**	-0.101268	1.218659	-0.613670
**C**	-0.083139	1.124902	0.812708
**C**	-0.018837	0.108859	-1.455439
**N**	-4.083791	0.444465	0.647793
**N**	4.083791	-0.444465	-0.647793
**C**	-3.612306	0.112671	2.091522
**C**	-3.494158	-0.563845	-0.329001
**C**	-5.599099	0.525100	0.547055
**C**	-3.523407	1.826010	0.284638
**C**	3.523407	-1.826010	-0.284638
**C**	5.599099	-0.525100	-0.547055
**C**	3.494158	0.563845	0.329001
**C**	3.612306	-0.112671	-2.091522
**H**	-2.448512	1.698630	0.308272
**H**	-3.816376	2.013899	-0.744280
**H**	-5.810496	0.982052	-0.417252
**H**	-5.910721	1.226763	1.317160
**H**	-2.775583	0.784043	2.263973
**H**	-4.427295	0.408910	2.747800
**H**	-2.417533	-0.523646	-0.185164
**H**	-3.836655	-1.542362	-0.009340
**H**	3.816376	-2.013899	0.744280
**H**	2.448512	-1.698630	-0.308272
**H**	5.810496	-0.982052	0.417252

**H**	5.910721	-1.226763	-1.317160
**H**	3.836655	1.542362	0.009340
**H**	2.417533	0.523646	0.185164
**H**	4.427295	-0.408910	-2.747800
**H**	2.775583	-0.784043	-2.263973
**C**	-6.379086	-0.769899	0.689048
**C**	-3.949075	3.002800	1.173035
**C**	-3.124991	-1.295173	2.443378
**C**	-3.825655	-0.364632	-1.803234
**C**	3.124991	1.295173	-2.443378
**C**	3.825655	0.364632	1.803234
**C**	3.949075	-3.002800	-1.173035
**C**	6.379086	0.769899	-0.689048
**H**	-6.235649	-1.189509	1.682234
**H**	-6.036511	-1.511384	-0.034140
**H**	-3.053807	3.613376	1.302261
**H**	-4.217445	2.668281	2.175813
**H**	-2.629264	-1.137286	3.405347
**H**	-2.327237	-1.602441	1.769238
**H**	-3.370013	0.551572	-2.179555
**H**	-4.903033	-0.282368	-1.956315
**H**	3.053807	-3.613376	-1.302261
**H**	4.217445	-2.668281	-2.175813
**H**	3.370013	-0.551572	2.179555
**H**	4.903033	0.282368	1.956315
**H**	2.629264	1.137286	-3.405347
**H**	2.327237	1.602441	-1.769238
**H**	6.235649	1.189509	-1.682234
**H**	6.036511	1.511384	0.034140
**C**	-5.063220	3.875332	0.588777
**C**	-4.142172	-2.420592	2.615476
**C**	-3.287588	-1.555446	-2.603037
**C**	-7.869643	-0.497814	0.465106
**H**	-8.251092	0.229715	1.185041
**H**	-8.054004	-0.109230	-0.538918
**H**	-8.443829	-1.417671	0.580840
**H**	-4.778470	4.255333	-0.394966
**H**	-6.003691	3.334787	0.476290
**H**	-5.250302	4.733640	1.236335
**H**	-4.946322	-2.132852	3.296170
**H**	-4.591507	-2.738574	1.674994
**H**	-3.643123	-3.291732	3.043649
**H**	-2.210982	-1.665211	-2.465937
**H**	-3.767958	-2.486139	-2.291729

**H**	-3.483714	-1.415218	-3.666848
**C**	5.063220	-3.875332	-0.588777
**C**	4.142172	2.420592	-2.615476
**C**	3.287588	1.555446	2.603037
**C**	7.869643	0.497814	-0.465106
**H**	6.003691	-3.334787	-0.476290
**H**	4.778470	-4.255333	0.394966
**H**	5.250302	-4.733640	-1.236335
**H**	3.767958	2.486139	2.291729
**H**	2.210982	1.665211	2.465937
**H**	3.483714	1.415218	3.666848
**H**	4.591507	2.738574	-1.674994
**H**	4.946322	2.132852	-3.296170
**H**	3.643123	3.291732	-3.043649
**H**	8.054004	0.109230	0.538918
**H**	8.251092	-0.229715	-1.185041
**H**	8.443829	1.417671	-0.580840
**H**	0.112793	-1.141903	3.058118
**H**	-0.112793	1.141903	-3.058118

Charged

Oxidised (2-)

*Table S 32: **2**$^{2-}$ Oxidised- Coordinates (B3LYP/TZVP, PCM (Water))*

Element	x	y	z
**O**	0.000000	2.763817	0.000001
**N**	-2.494945	1.106791	0.000000
**N**	-3.250670	0.000000	0.000001
**N**	-2.494945	-1.106791	-0.000002
**C**	-1.213183	0.701095	-0.000001
**C**	-1.213183	-0.701095	0.000000
**C**	0.000000	1.534688	-0.000001
**O**	0.000000	-2.763817	-0.000002
**N**	2.494945	-1.106791	0.000001
**N**	3.250670	0.000000	0.000000
**N**	2.494945	1.106791	0.000002
**C**	1.213183	-0.701095	0.000000
**C**	1.213183	0.701095	0.000000
**C**	0.000000	-1.534688	0.000000

Singly reduced (3-)

*Table S 33: **2**$^{3-\cdot}$ - Coordinates (B3LYP/TZVP, PCM (Water))*

Element	x	y	z
**O**	0.000000	2.807365	0.000000
**N**	2.493419	1.108953	0.000001
**N**	3.241999	0.000000	0.000000
**N**	2.493419	-1.108953	0.000000
**C**	1.190970	0.709154	0.000000
**C**	1.190970	-0.709154	0.000001
**C**	0.000000	1.532929	0.000000
**O**	0.000000	-2.807365	0.000000
**N**	-2.493419	-1.108953	-0.000002
**N**	-3.241999	0.000000	0.000000
**N**	-2.493419	1.108953	-0.000001
**C**	-1.190970	-0.709154	0.000000
**C**	-1.190970	0.709154	-0.000001
**C**	0.000000	-1.532929	0.000002

Singly reduced (protonated, 2-)

*Table S 34: **2**$^{2-\cdot}$ - Coordinates (B3LYP/TZVP, PCM (Water))*

Element	x	y	z
**O**	-0.042042	2.778515	0.000001
**N**	-2.484849	1.043495	0.000000
**N**	-3.229272	-0.071984	-0.000001
**N**	-2.474937	-1.174899	-0.000001
**C**	-1.191527	0.635642	0.000000
**C**	-1.179507	-0.776295	-0.000001
**C**	-0.019978	1.421122	0.000000
**O**	0.066881	-2.827003	0.000000
**N**	2.523781	-1.083948	-0.000001
**N**	3.247538	0.039841	0.000002
**N**	2.477615	1.134968	0.000001
**C**	1.219758	-0.719924	0.000000
**C**	1.191305	0.698322	0.000001
**C**	0.038163	-1.574056	-0.000001
**H**	-0.967133	3.066737	0.000001

Doubly reduced (4-)

*Table S 35: **2**$^{4-}$ - Coordinates (B3LYP/TZVP, PCM (Water))*

Element	x	y	z
**O**	0.000000	2.862976	-0.000001
**N**	2.495117	1.111383	0.000001
**N**	3.235864	0.000000	0.000000
**N**	2.495117	-1.111383	0.000002
**C**	1.168206	0.719767	0.000000
**C**	1.168206	-0.719767	0.000001
**C**	0.000000	1.539679	0.000000
**O**	0.000000	-2.862976	0.000001
**N**	-2.495117	-1.111383	-0.000001
**N**	-3.235864	0.000000	0.000000
**N**	-2.495117	1.111383	-0.000002
**C**	-1.168206	-0.719767	0.000000
**C**	-1.168206	0.719767	-0.000001
**C**	0.000000	-1.539679	0.000000

Doubly reduced (protonated, 2-)

*Table S 36: **2**$^{2-}$ Reduced - Coordinates (B3LYP/TZVP, PCM (Water))*

Element	x	y	z
**O**	-0.000877	2.841106	-0.000407
**N**	2.475689	1.125533	-0.001998
**N**	3.227819	0.023551	-0.000058
**N**	2.510857	-1.100500	0.001663
**C**	1.171619	0.704406	-0.002796
**C**	1.189762	-0.724044	0.002085
**C**	-0.002932	1.457426	0.001873
**O**	0.000877	-2.841106	0.000407
**N**	-2.475689	-1.125533	0.001998
**N**	-3.227819	-0.023551	0.000058
**N**	-2.510857	1.100500	-0.001663
**C**	-1.171619	-0.704406	0.002796
**C**	-1.189762	0.724044	-0.002085
**C**	0.002932	-1.457426	-0.001873
**H**	0.922632	3.127214	0.001911
**H**	-0.922632	-3.127214	-0.001911

Protonated variants

one proton

version 1

*Table S 37: **2**$^{2-\cdot}$ - Coordinates (B3LYP/TZVP, PCM (Water))*

Element	x	y	z
**O**	-0.071312	-2.789673	-0.000004
**N**	2.500853	-1.150097	0.000006
**N**	3.269024	-0.055276	0.000009
**N**	2.545825	1.062213	0.000016
**C**	1.213910	-0.726264	-0.000005
**C**	1.239985	0.690430	-0.000008
**C**	0.006958	-1.523296	-0.000016
**O**	0.084093	2.805872	0.000000
**N**	-2.442746	1.169092	0.000018
**N**	-3.229165	0.136744	0.000002
**N**	-2.461162	-0.974169	0.000009
**C**	-1.139778	0.746171	-0.000008
**C**	-1.139823	-0.660038	-0.000005
**C**	0.069801	1.545891	-0.000026
**H**	-2.886975	-1.886509	0.000012

version 2

*Table S 38: **2**$^{2-\cdot}$ - Coordinates (B3LYP/TZVP, PCM (Water))*

Element	x	y	z
**O**	0.026980	2.808257	-0.000001
**N**	2.524144	1.108619	-0.000001
**N**	3.269385	0.000000	0.000003
**N**	2.524144	-1.108619	0.000002
**C**	1.223323	0.710515	-0.000001
**C**	1.223323	-0.710515	-0.000001
**C**	0.044004	1.543043	-0.000003
**O**	0.026980	-2.808257	0.000001
**N**	-2.434190	-1.132598	0.000004
**N**	-3.119697	0.000000	-0.000001
**N**	-2.434190	1.132598	0.000002
**C**	-1.151450	-0.716472	-0.000001
**C**	-1.151450	0.716472	-0.000001
**C**	0.044004	-1.543043	-0.000004
**H**	-4.129392	0.000000	-0.000002

two protons

version 1

*Table S 39: **2**$^{1-·}$ - Coordinates (B3LYP/TZVP, PCM (Water))*

Element	x	y	z
**O**	-0.035650	-2.794818	0.000001
**N**	2.521354	-1.156261	0.000000
**N**	3.303975	-0.056826	-0.000001
**N**	2.590102	1.058864	-0.000004
**C**	1.247543	-0.724649	0.000000
**C**	1.282553	0.692711	0.000000
**C**	0.034462	-1.535169	0.000001
**O**	0.120074	2.799184	-0.000001
**N**	-2.358023	1.202549	-0.000001
**N**	-3.129377	0.153226	0.000001
**N**	-2.429529	-1.009418	0.000000
**C**	-1.106212	0.747569	0.000001
**C**	-1.107865	-0.681963	0.000001
**C**	0.126115	1.558836	0.000003
**H**	-4.139885	0.187791	0.000000
**H**	-2.884609	-1.911667	0.000001

version 2

*Table S 40: **2**$^{1-·}$ - Coordinates (B3LYP/TZVP, PCM (Water))*

Element	x	y	z
**O**	0.011233	2.764904	-0.000003
**N**	-2.502275	1.079382	0.000003
**N**	-3.262938	-0.021052	0.000006
**N**	-2.528329	-1.138308	-0.000001
**C**	-1.218540	0.650755	-0.000001
**C**	-1.231128	-0.762739	-0.000001
**C**	-0.037258	1.419217	-0.000003
**O**	-0.006706	-2.828549	-0.000003
**N**	2.474723	-1.147156	-0.000003
**N**	3.244719	-0.102037	0.000011
**N**	2.457875	1.000795	0.000002
**C**	1.171027	-0.748879	-0.000001
**C**	1.145854	0.656942	-0.000001
**C**	-0.031162	-1.586683	-0.000007
**H**	2.876803	1.919042	0.000003
**H**	-0.892194	3.117068	-0.000004

version 3

Table S 41: $\mathbf{2}^{1-\cdot}$ - Coordinates (B3LYP/TZVP, PCM (Water))

Element	x	y	z
**O**	-0.069338	-2.826399	-0.000001
**N**	-2.546781	-1.102114	0.000003
**N**	-3.272396	0.017892	0.000001
**N**	-2.510335	1.116270	0.000001
**C**	-1.246442	-0.732696	-0.000001
**C**	-1.222746	0.688474	0.000000
**C**	-0.072627	-1.583932	-0.000004
**O**	-0.037551	2.784419	-0.000001
**N**	2.432422	1.080273	-0.000002
**N**	3.114372	-0.059154	0.000005
**N**	2.420581	-1.183077	-0.000001
**C**	1.155559	0.657773	-0.000001
**C**	1.148305	-0.767322	-0.000002
**C**	-0.029602	1.435822	-0.000001
**H**	4.124955	-0.064544	0.000009
**H**	0.870435	3.121030	-0.000002

three protons

Table S 42: $\mathbf{2}^{\cdot}$ - Coordinates (B3LYP/TZVP, PCM (Water))

Element	x	y	z
**O**	-0.013213	2.762785	0.000001
**N**	-2.524395	1.086005	0.000004
**N**	-3.292317	-0.007974	-0.000003
**N**	-2.567302	-1.130567	-0.000005
**C**	-1.246994	0.650886	-0.000001
**C**	-1.271228	-0.764977	0.000002
**C**	-0.069603	1.428026	0.000000
**O**	-0.045463	-2.830578	-0.000001
**N**	2.398547	-1.176580	-0.000003
**N**	3.136550	-0.109339	0.000000
**N**	2.412627	1.029426	0.000001
**C**	1.134056	-0.756484	0.000002
**C**	1.111692	0.668555	0.000000
**C**	-0.085795	-1.601866	0.000005
**H**	4.150286	-0.113543	0.000001
**H**	2.851788	1.943160	0.000002
**H**	-0.911397	3.131090	0.000000

4H,8H-benzo[1,2-c:4,5-c']bis([1,2,5]thiadiazole)-4,8-dione (**3**)

Coordinates (B3LYP/TZVP, PCM (Water))

Oxidised

*Table S 43: **3** Oxidised - Coordinates (B3LYP/TZVP, PCM (Water))*

Element	x	y	z
**S**	-3.530092	-0.000017	0.000000
**O**	-0.000013	2.753071	0.001601
**N**	-2.445033	1.251700	0.000932
**N**	-2.445021	-1.251723	-0.000933
**C**	-0.000007	1.543710	0.000984
**C**	-1.242840	0.717362	0.000556
**C**	-1.242833	-0.717374	-0.000557
**S**	3.530092	0.000017	0.000000
**O**	0.000013	-2.753071	-0.001601
**N**	2.445033	-1.251700	-0.000932
**N**	2.445021	1.251723	0.000933
**C**	0.000007	-1.543710	-0.000984
**C**	1.242840	-0.717362	-0.000556
**C**	1.242833	0.717374	0.000557

Reduced

*Table S 44: **3** Reduced - Coordinates (B3LYP/TZVP, PCM (Water))*

Element	x	y	z
**S**	3.471241	0.042617	-0.000012
**O**	0.025436	-2.829955	0.000013
**N**	2.436590	-1.221325	0.000001
**N**	2.419580	1.290615	0.000015
**C**	0.023382	-1.475756	0.000006
**C**	1.191446	-0.704241	0.000000
**C**	1.178923	0.761078	0.000005
**S**	-3.471241	-0.042617	-0.000008
**O**	-0.025436	2.829955	0.000000
**N**	-2.436590	1.221325	0.000010
**N**	-2.419580	-1.290615	-0.000005
**C**	-0.023382	1.475756	-0.000001
**C**	-1.191446	0.704241	0.000002
**C**	-1.178923	-0.761077	-0.000003
**H**	0.941679	-3.144670	-0.000002
**H**	-0.941679	3.144670	0.000016

2,6-dimethylbenzo[1,2-d:4,5-d']diimidazole-4,8(1H,5H)-dione (**4**)

Coordinates (B3LYP/TZVP, PCM (Water))

Oxidised

*Table S 45: **4** Oxidised - Coordinates (B3LYP/TZVP, PCM (Water))*

Element	x	y	z
**O**	2.370645	1.386017	0.000000
**N**	-0.308567	2.701711	0.000000
**N**	-2.248852	1.625576	0.000000
**C**	-0.003472	1.371703	0.000000
**C**	-1.224461	0.722069	0.000000
**C**	1.326583	0.749542	0.000000
**O**	-2.370645	-1.386017	0.000000
**N**	0.308567	-2.701711	0.000000
**N**	2.248852	-1.625576	0.000000
**C**	0.003472	-1.371703	0.000000
**C**	1.224461	-0.722069	0.000000
**C**	-1.326583	-0.749542	0.000000
**C**	1.670903	-2.818268	0.000000
**C**	-1.670903	2.818268	0.000000
**H**	-0.361997	-3.458011	0.000000
**H**	0.361997	3.458011	0.000000
**C**	2.370645	-4.129737	0.000000
**H**	2.103793	-4.714904	0.882539
**H**	2.103793	-4.714904	-0.882539
**H**	3.445425	-3.963580	0.000000
**C**	-2.370645	4.129737	0.000000
**H**	-2.103793	4.714904	0.882539
**H**	-2.103793	4.714904	-0.882539
**H**	-3.445425	3.963580	0.000000

Reduced

*Table S 46: **4** Reduced - Coordinates (B3LYP/TZVP, PCM (Water))*

Element	x	y	z
**O**	0.009282	2.829344	0.000028
**N**	-2.492315	1.093410	0.000010
**N**	-2.502742	-1.138021	-0.000003
**C**	-1.155941	0.725509	0.000008
**C**	-1.187412	-0.692913	-0.000002
**C**	0.021006	1.458091	0.000014
**O**	-0.009282	-2.829344	-0.000010

**N**	2.492315	-1.093410	0.000004
**N**	2.502742	1.138021	0.000025
**C**	1.155941	-0.725509	-0.000002
**C**	1.187412	0.692913	0.000008
**C**	-0.021006	-1.458091	-0.000006
**C**	3.245417	0.054123	-0.000008
**C**	-3.245417	-0.054123	-0.000018
**H**	2.848475	-2.035771	0.000007
**H**	-2.848475	2.035771	0.000017
**C**	4.733264	0.018548	-0.000031
**H**	5.111671	-0.503643	0.881805
**H**	5.111648	-0.503374	-0.882037
**H**	5.121096	1.034787	0.000116
**C**	-4.733264	-0.018548	-0.000014
**H**	-5.111659	0.503380	0.881983
**H**	-5.111660	0.503637	-0.881858
**H**	-5.121096	-1.034787	-0.000158
**H**	-0.925793	-3.139442	-0.000011
**H**	0.925793	3.139442	0.000032

2,6-di(pyridin-2-yl)benzo[1,2-d:4,5-d']diimidazole-4,8(1H,5H)-dione (**5**)

Coordinates (B3LYP/TZVP, PCM (Water))

Oxidised

*Table S 47: **5** Oxidised - Coordinates (B3LYP/TZVP, PCM (Water))*

Element	x	y	z
**O**	-0.080329	2.747752	-0.000050
**N**	-2.515015	1.025395	-0.000019
**N**	-2.496440	-1.199458	0.000021
**C**	-1.206848	0.657215	-0.000021
**C**	-1.219733	-0.731629	0.000010
**C**	-0.023331	1.527919	-0.000042
**O**	0.080329	-2.747752	0.000038
**N**	2.515015	-1.025395	0.000011
**N**	2.496440	1.199458	-0.000029
**C**	1.206848	-0.657215	0.000008
**C**	1.219733	0.731629	-0.000022
**C**	0.023331	-1.527919	0.000025
**C**	3.264037	0.115375	-0.000007
**C**	-3.264037	-0.115375	0.000004
**H**	2.891895	-1.964219	0.000029
**H**	-2.891895	1.964219	-0.000036

**C**	4.726248	0.076152	-0.000003
**C**	5.480116	1.250569	-0.000024
**C**	6.604841	-1.235162	0.000025
**C**	6.864010	1.142617	-0.000019
**H**	4.985240	2.211461	-0.000042
**H**	7.020309	-2.236594	0.000045
**H**	7.481148	2.031776	-0.000034
**C**	-4.726248	-0.076152	0.000008
**C**	-5.480116	-1.250569	0.000032
**C**	-6.604841	1.235162	-0.000011
**C**	-6.864010	-1.142617	0.000034
**H**	-4.985240	-2.211461	0.000048
**H**	-7.020309	2.236594	-0.000029
**H**	-7.481148	-2.031776	0.000053
**C**	7.442059	-0.122144	0.000006
**H**	8.516112	-0.249781	0.000011
**C**	-7.442059	0.122144	0.000012
**H**	-8.516112	0.249781	0.000013
**N**	5.274954	-1.149196	0.000021
**N**	-5.274954	1.149196	-0.000014

Reduced

*Table S 48: **5** Reduced - Coordinates (B3LYP/TZVP, PCM (Water))*

Element	x	y	z
**O**	-0.028021	2.831549	-0.000005
**N**	-2.498878	1.067030	-0.000003
**N**	-2.479246	-1.170121	0.000000
**C**	-1.164085	0.715401	-0.000002
**C**	-1.177702	-0.710077	0.000001
**C**	0.001976	1.463707	-0.000002
**O**	0.028021	-2.831549	0.000005
**N**	2.498878	-1.067030	0.000003
**N**	2.479246	1.170121	0.000000
**C**	1.164085	-0.715401	0.000002
**C**	1.177702	0.710077	-0.000001
**C**	-0.001976	-1.463707	0.000002
**C**	3.232962	0.088715	0.000000
**C**	-3.232962	-0.088715	0.000000
**H**	2.896628	-1.993418	0.000005
**H**	-2.896628	1.993418	-0.000005
**C**	4.696835	0.056063	0.000000
**C**	5.445300	1.235623	-0.000002
**C**	6.589562	-1.238985	0.000002

**C**	6.829200	1.139666	-0.000002
**H**	4.942347	2.192281	-0.000003
**H**	7.012863	-2.237376	0.000003
**H**	7.439088	2.034007	-0.000003
**C**	-4.696835	-0.056063	0.000000
**C**	-5.445300	-1.235623	0.000002
**C**	-6.589562	1.238985	-0.000002
**C**	-6.829200	-1.139666	0.000002
**H**	-4.942347	-2.192281	0.000003
**H**	-7.012863	2.237376	-0.000003
**H**	-7.439088	-2.034007	0.000003
**C**	7.418503	-0.120831	0.000000
**H**	8.493615	-0.239901	0.000000
**C**	-7.418503	0.120831	0.000000
**H**	-8.493615	0.239901	0.000000
**N**	5.258161	-1.164305	0.000002
**N**	-5.258161	1.164305	-0.000002
**H**	-0.882845	-3.158106	0.000005
**H**	0.882845	3.158106	-0.000005

2,6-di(pyridin-3-yl)benzo[1,2-d:4,5-d']diimidazole-4,8(1H,5H)-dione (**6**)

Coordinates (B3LYP/TZVP, PCM (Water))

Oxidised

*Table S 49: **6** Oxidised - Coordinates (B3LYP/TZVP, PCM (Water))*

Element	x	y	Z
**O**	-0.011638	2.736805	0.246071
**N**	-2.488789	1.086949	0.093482
**N**	-2.525841	-1.126919	-0.103194
**C**	-1.188700	0.683677	0.058386
**C**	-1.238017	-0.695608	-0.064958
**C**	0.014998	1.520937	0.136772
**O**	0.011639	-2.736802	-0.246050
**N**	2.488789	-1.086947	-0.093460
**N**	2.525842	1.126923	0.103200
**C**	1.188700	-0.683675	-0.058356
**C**	1.238017	0.695611	0.064978
**C**	-0.014998	-1.520935	-0.136748
**C**	3.273411	0.031652	0.006897
**C**	-3.273411	-0.031649	-0.006887
**H**	2.791116	-2.044187	-0.207899
**H**	-2.791113	2.044188	0.207936

**C**	4.733282	0.016633	0.003622
**C**	5.469021	-1.152798	0.228793
**C**	5.444992	1.200317	-0.218723
**H**	4.969678	-2.093566	0.434060
**C**	6.829249	1.160511	-0.214757
**H**	4.911153	2.124558	-0.393142
**H**	7.412230	2.055563	-0.385547
**C**	-4.733282	-0.016631	-0.003627
**C**	-5.469022	1.152818	-0.228696
**C**	-5.444992	-1.200338	0.218599
**H**	-4.969682	2.093609	-0.433861
**C**	-6.829249	-1.160536	0.214619
**H**	-4.911152	-2.124594	0.392938
**H**	-7.412228	-2.055606	0.385318
**C**	7.464124	-0.058708	0.006024
**C**	-7.464125	0.058700	-0.006057
**H**	8.546370	-0.123525	0.007611
**H**	-8.546371	0.123514	-0.007654
**N**	6.801048	-1.198774	0.227307
**N**	-6.801049	1.198789	-0.227223

Reduced

*Table S 50: **6** Reduced - Coordinates (B3LYP/TZVP, PCM (Water))*

Element	x	y	Z
**O**	-0.030368	-2.830213	-0.012740
**N**	2.474959	-1.122823	-0.016210
**N**	2.504595	1.112271	-0.003396
**C**	1.146876	-0.737996	-0.011838
**C**	1.192933	0.683341	-0.008288
**C**	-0.034055	-1.462357	-0.011323
**O**	0.030368	2.830213	-0.012737
**N**	-2.474959	1.122823	-0.016206
**N**	-2.504595	-1.112271	-0.003394
**C**	-1.146876	0.737996	-0.011835
**C**	-1.192933	-0.683341	-0.008287
**C**	0.034055	1.462357	-0.011321
**C**	-3.241354	-0.019353	-0.005075
**C**	3.241354	0.019353	-0.005079
**H**	-2.803352	2.072643	0.046212
**H**	2.803352	-2.072643	0.046206
**C**	-4.703189	-0.006974	0.002858
**C**	-5.443236	1.138047	-0.315876
**C**	-5.415093	-1.167226	0.327040

**H**	-4.946345	2.059007	-0.600996
**C**	-6.799382	-1.129665	0.325410
**H**	-4.878995	-2.072772	0.576656
**H**	-7.380524	-2.007836	0.573583
**C**	4.703189	0.006974	0.002853
**C**	5.443236	-1.138047	-0.315884
**C**	5.415093	1.167225	0.327035
**H**	4.946345	-2.059006	-0.601004
**C**	6.799383	1.129665	0.325403
**H**	4.878996	2.072771	0.576653
**H**	7.380525	2.007836	0.573576
**C**	-7.437446	0.065873	0.005057
**C**	7.437446	-0.065873	0.005048
**H**	-8.519898	0.129133	0.002117
**H**	8.519898	-0.129133	0.002107
**N**	-6.776229	1.183403	-0.313559
**N**	6.776229	-1.183403	-0.313569
**H**	-0.946849	-3.140553	-0.011173
**H**	0.946849	3.140553	-0.011170

2,2'-(4,8-dioxo-1,4,5,8-tetrahydrobenzo[1,2-d:4,5-d']diimidazole-2,6-diyl)bis(1-methylpyridin-1-ium) diiodide (**7**)

Coordinates (B3LYP/TZVP, PCM (Water))

Oxidised

*Table S 51: **7** Oxidised - Coordinates (B3LYP/TZVP, PCM (Water))*

Element	x	y	z
**O**	-0.079962	-2.552596	-1.012952
**N**	2.446621	-1.104930	-0.392247
**N**	2.558171	0.965435	0.420543
**C**	1.166961	-0.683963	-0.238260
**C**	1.258443	0.603661	0.272379
**C**	-0.067899	-1.423560	-0.559950
**O**	0.079065	2.548968	1.032091
**N**	-2.448199	1.105521	0.399520
**N**	-2.559044	-0.967834	-0.406187
**C**	-1.168023	0.681902	0.253400
**C**	-1.259294	-0.607192	-0.253606
**C**	0.066780	1.420165	0.578546
**C**	-3.260566	0.080434	-0.005676
**C**	3.260228	-0.080013	0.012631
**H**	-2.728444	1.995335	0.792782

**H**	2.724684	-1.992461	-0.791979
**C**	-4.717172	0.219117	-0.067090
**C**	-5.291449	1.389515	-0.542524
**C**	-6.861653	-0.709571	0.218650
**C**	-6.670939	1.507694	-0.633261
**H**	-4.648967	2.194897	-0.867321
**H**	-7.430523	-1.569501	0.536279
**H**	-7.114424	2.419538	-1.008379
**C**	4.717044	-0.218614	0.056955
**C**	5.295989	-1.408230	0.477708
**C**	6.860865	0.714798	-0.221709
**C**	6.675941	-1.533922	0.542744
**H**	4.657449	-2.224648	0.781821
**H**	7.427651	1.585684	-0.511994
**H**	7.121699	-2.461214	0.874797
**C**	-7.465768	0.435257	-0.255320
**H**	-8.543034	0.473572	-0.318160
**C**	7.468751	-0.449832	0.194837
**H**	8.546739	-0.493570	0.238793
**C**	-4.956407	-2.066721	0.885681
**H**	-4.493274	-2.641993	0.090817
**H**	-4.215142	-1.815217	1.638356
**H**	-5.767671	-2.624123	1.340019
**C**	4.953820	2.107815	-0.798332
**H**	4.200413	1.895714	-1.550800
**H**	4.504973	2.648381	0.028370
**H**	5.762378	2.681178	-1.237449
**N**	-5.520390	-0.812988	0.321909
**N**	5.518378	0.826941	-0.300094

Reduced

*Table S 52: **7** Reduced - Coordinates (B3LYP/TZVP, PCM (Water))*

Element	x	y	z
**O**	-0.034929	2.790840	0.970617
**N**	2.467500	1.208405	0.366353
**N**	2.502797	-0.898830	-0.392028
**C**	1.143995	0.839914	0.235718
**C**	1.193722	-0.497207	-0.256921
**C**	-0.037587	1.515203	0.495497
**O**	0.038256	-2.514472	-1.005728
**N**	-2.462843	-0.930045	-0.411652
**N**	-2.500866	1.176593	0.347862
**C**	-1.138791	-0.559355	-0.279915

**C**	-1.189604	0.776252	0.214494
**C**	0.041436	-1.237640	-0.534213
**C**	-3.223768	0.145286	-0.027834
**C**	3.227757	0.129412	-0.011467
**H**	-2.785997	-1.777937	-0.850196
**H**	2.800057	2.066112	0.780201
**C**	-4.687685	0.204442	-0.095430
**C**	-5.290722	1.339045	-0.623950
**C**	-6.815284	-0.753170	0.246929
**C**	-6.670273	1.418503	-0.722858
**H**	-4.654115	2.140745	-0.966142
**H**	-7.362491	-1.605622	0.618820
**H**	-7.135777	2.300393	-1.140842
**C**	4.688089	0.212283	-0.070122
**C**	5.307020	1.376571	-0.510251
**C**	6.803333	-0.795191	0.186150
**C**	6.688028	1.449891	-0.600202
**H**	4.693001	2.212588	-0.811200
**H**	7.341973	-1.683109	0.478131
**H**	7.162242	2.356425	-0.949942
**C**	-7.442791	0.345208	-0.294671
**H**	-8.520368	0.354681	-0.363542
**C**	7.447006	0.339821	-0.253934
**H**	8.524966	0.342944	-0.316394
**C**	-4.891105	-2.014922	1.017500
**H**	-4.628170	-2.756522	0.266613
**H**	-4.017539	-1.726714	1.592188
**H**	-5.640174	-2.427023	1.684958
**C**	4.858769	-2.109609	0.814423
**H**	4.108961	-1.859857	1.558628
**H**	4.395190	-2.656739	0.000352
**H**	5.651281	-2.694164	1.268384
**N**	-5.469135	-0.816865	0.357978
**N**	5.457962	-0.856702	0.287812
**H**	0.951693	-2.812495	-1.122074
**H**	-0.948540	3.089033	1.085016

4,4'-(4,8-dioxo-1,4,5,8-tetrahydrobenzo[1,2-d:4,5-d']diimidazole-2,6-diyl)bis(1-methylpyridin-1-ium) diiodide (**8**)

Coordinates (B3LYP/TZVP, PCM (Water))

Oxidised

*Table S 53: **8** Oxidised - Coordinates (B3LYP/TZVP, PCM (Water))*

Element	x	y	z
**O**	0.020552	-2.750139	-0.029025
**N**	-2.466679	-1.124752	-0.015596
**N**	-2.535514	1.099722	0.005821
**C**	-1.180500	-0.701536	-0.009529
**C**	-1.246901	0.687507	0.004654
**C**	0.036587	-1.533213	-0.016096
**O**	-0.020552	2.750139	0.029023
**N**	2.466679	1.124752	0.015595
**N**	2.535514	-1.099722	-0.005823
**C**	1.180500	0.701536	0.009529
**C**	1.246901	-0.687506	-0.004655
**C**	-0.036587	1.533213	0.016096
**C**	3.265403	0.010619	0.006151
**C**	-3.265403	-0.010619	-0.006153
**H**	2.747954	2.096075	0.032072
**H**	-2.747954	-2.096075	-0.032069
**C**	4.719685	0.030348	0.006103
**C**	5.464207	1.214525	-0.017409
**C**	5.419048	-1.184344	0.032919
**C**	6.838881	1.158122	-0.014148
**H**	5.004653	2.191123	-0.040172
**C**	6.791171	-1.182939	0.036150
**H**	4.889013	-2.124325	0.054095
**H**	7.449267	2.047712	-0.032686
**H**	7.371124	-2.093147	0.055666
**C**	-4.719685	-0.030348	-0.006104
**C**	-5.464207	-1.214525	0.017405
**C**	-5.419048	1.184344	-0.032917
**C**	-6.838881	-1.158122	0.014146
**H**	-5.004653	-2.191124	0.040165
**C**	-6.791171	1.182939	-0.036147
**H**	-4.889013	2.124325	-0.054091
**H**	-7.449267	-2.047712	0.032683
**H**	-7.371124	2.093147	-0.055660
**N**	7.486886	-0.023610	0.013190
**N**	-7.486886	0.023610	-0.013188

**C**	8.966155	-0.065111	-0.032279
**H**	9.280205	-0.269878	-1.053996
**H**	9.358285	0.892986	0.293164
**H**	9.317025	-0.849453	0.632087
**C**	-8.966155	0.065110	0.032283
**H**	-9.280203	0.269866	1.054003
**H**	-9.317027	0.849459	-0.632074
**H**	-9.358285	-0.892984	-0.293168

Reduced

*Table S 54: **8** Reduced - Coordinates (B3LYP/TZVP, PCM (Water))*

Element	x	y	z
**O**	0.066170	2.831282	-0.018261
**N**	-2.450542	1.164567	-0.014918
**N**	-2.509207	-1.072725	-0.016013
**C**	-1.133444	0.759887	-0.019126
**C**	-1.200175	-0.667439	-0.019030
**C**	0.054448	1.470158	-0.019507
**O**	-0.066170	-2.831282	-0.018310
**N**	2.450542	-1.164566	-0.014939
**N**	2.509207	1.072726	-0.015996
**C**	1.133444	-0.759887	-0.019139
**C**	1.200175	0.667440	-0.019019
**C**	-0.054448	-1.470158	-0.019533
**C**	3.229885	-0.033517	-0.013548
**C**	-3.229885	0.033518	-0.013546
**H**	2.758684	-2.123724	-0.026554
**H**	-2.758684	2.123724	-0.026515
**C**	4.683468	-0.044252	-0.002943
**C**	5.435128	-1.225278	0.028494
**C**	5.381566	1.172806	-0.026378
**C**	6.809209	-1.164093	0.035447
**H**	4.978807	-2.203330	0.049644
**C**	6.752661	1.177017	-0.018923
**H**	4.847249	2.110163	-0.052853
**H**	7.422848	-2.051369	0.059992
**H**	7.329831	2.089111	-0.034854
**C**	-4.683468	0.044252	-0.002940
**C**	-5.381566	-1.172805	-0.026387
**C**	-5.435128	1.225278	0.028509
**C**	-6.752661	-1.177017	-0.018932
**H**	-4.847249	-2.110162	-0.052871
**C**	-6.809209	1.164093	0.035461

**H**	-4.978807	2.203329	0.049669
**H**	-7.329831	-2.089111	-0.034872
**H**	-7.422847	2.051368	0.060015
**N**	7.454034	0.019727	0.010788
**N**	-7.454034	-0.019727	0.010791
**C**	8.931865	0.067465	0.067065
**H**	9.238920	0.283955	1.088586
**H**	9.330297	-0.892412	-0.245476
**H**	9.285593	0.845859	-0.602966
**C**	-8.931865	-0.067466	0.067066
**H**	-9.238921	-0.283978	1.088582
**H**	-9.330297	0.892417	-0.245456
**H**	-9.285593	-0.845847	-0.602982
**H**	-0.983018	-3.141252	-0.017553
**H**	0.983018	3.141252	-0.017500

3,3'-(4,8-dioxo-1,4,5,8-tetrahydrobenzo[1,2-d:4,5-d']diimidazole-2,6-diyl)bis(1-methylpyridin-1-ium) diiodide (**9**)

Coordinates (B3LYP/TZVP, PCM (Water))

Oxidised

*Table S 55: **9** Oxidised - Coordinates (B3LYP/TZVP, PCM (Water))*

Element	x	y	z
**O**	-0.257872	-2.736582	-0.011452
**N**	2.366262	-1.332275	-0.000622
**N**	2.622478	0.876906	0.008257
**C**	1.116199	-0.799744	-0.000568
**C**	1.300247	0.575302	0.004910
**C**	-0.166379	-1.521846	-0.006320
**O**	0.257872	2.736582	0.011459
**N**	-2.366262	1.332274	0.000630
**N**	-2.622478	-0.876906	-0.008251
**C**	-1.116199	0.799744	0.000575
**C**	-1.300247	-0.575302	-0.004903
**C**	0.166379	1.521846	0.006328
**C**	-3.253129	0.290687	-0.004845
**C**	3.253129	-0.290687	0.004849
**H**	-2.564788	2.323742	0.004118
**H**	2.564788	-2.323743	-0.004110
**C**	-4.706209	0.433093	-0.003708
**C**	-5.472115	-0.728332	-0.010350
**C**	-5.372156	1.661240	-0.001097

**H**	-5.015473	-1.705777	-0.013336
**C**	-6.759410	1.689801	-0.001130
**H**	-4.829927	2.596151	0.001219
**H**	-7.297122	2.626172	0.000556
**C**	4.706209	-0.433093	0.003707
**C**	5.472115	0.728332	0.010347
**C**	5.372156	-1.661240	0.001096
**H**	5.015473	1.705777	0.013332
**C**	6.759410	-1.689801	0.001124
**H**	4.829927	-2.596151	-0.001218
**H**	7.297122	-2.626172	-0.000562
**C**	-7.466526	0.507839	-0.006977
**C**	7.466526	-0.507838	0.006968
**H**	-8.544768	0.464672	-0.005738
**H**	8.544768	-0.464672	0.005725
**N**	-6.812348	-0.674609	-0.013061
**N**	6.812348	0.674610	0.013053
**C**	-7.599883	-1.931461	0.024245
**H**	-6.972837	-2.752336	-0.307477
**H**	-7.932866	-2.101908	1.045746
**H**	-8.453822	-1.828992	-0.638976
**C**	7.599883	1.931461	-0.024257
**H**	6.972836	2.752338	0.307457
**H**	8.453819	1.828995	0.638969
**H**	7.932871	2.101902	-1.045757

Reduced

*Table S 56: **9** Reduced - Coordinates (B3LYP/TZVP, PCM (Water))*

Element	x	y	z
**O**	-0.308553	2.813963	-0.023446
**N**	2.346594	1.367928	-0.004034
**N**	2.596850	-0.854239	0.013871
**C**	1.065445	0.850967	-0.003494
**C**	1.252851	-0.561592	0.008128
**C**	-0.179810	1.456871	-0.012322
**O**	0.308553	-2.813963	0.023451
**N**	-2.346594	-1.367928	0.004038
**N**	-2.596850	0.854238	-0.013866
**C**	-1.065445	-0.850967	0.003499
**C**	-1.252851	0.561592	-0.008123
**C**	0.179810	-1.456871	0.012327
**C**	-3.216734	-0.307957	-0.006163
**C**	3.216734	0.307957	0.006165

**H**	-2.572761	-2.349542	0.015205
**H**	2.572761	2.349542	-0.015200
**C**	-4.670354	-0.446522	-0.005586
**C**	-5.436470	0.715502	-0.005683
**C**	-5.339904	-1.673120	-0.010039
**H**	-4.977170	1.691645	-0.003138
**C**	-6.727773	-1.699675	-0.010215
**H**	-4.798814	-2.608635	-0.013676
**H**	-7.266884	-2.635327	-0.014005
**C**	4.670354	0.446522	0.005585
**C**	5.436471	-0.715502	0.005681
**C**	5.339904	1.673120	0.010036
**H**	4.977170	-1.691645	0.003138
**C**	6.727773	1.699675	0.010208
**H**	4.798814	2.608635	0.013673
**H**	7.266884	2.635328	0.013997
**C**	-7.433718	-0.517383	-0.009558
**C**	7.433718	0.517383	0.009551
**H**	-8.511802	-0.471822	-0.008288
**H**	8.511802	0.471822	0.008278
**N**	-6.776439	0.664372	-0.008958
**N**	6.776439	-0.664372	0.008953
**H**	1.249064	-3.041606	0.027267
**H**	-1.249064	3.041606	-0.027261
**C**	-7.562029	1.921583	0.034638
**H**	-6.930600	2.745303	-0.281470
**H**	-8.409651	1.828506	-0.638147
**H**	-7.905320	2.081933	1.054388
**C**	7.562029	-1.921583	-0.034644
**H**	6.930602	-2.745302	0.281468
**H**	7.905316	-2.081934	-1.054395
**H**	8.409654	-1.828505	0.638138

DFT only compounds coordinates

Bis-(1-methylimidazolo)-bis-imidazolo-1,4-benzoquinone (**4**$_{\mathbf{MeImid}}$)

Oxidised

*Table S 57: **4**$_{MeImid}$ Oxidised - Coordinates (B3LYP/TZVP, PCM (Water))*

Element	x	y	z
**O**	0.143665	2.744122	-0.000015
**N**	-2.424821	1.228594	-0.000001
**N**	-2.585910	-0.994313	0.000001

**C**	-1.149654	0.752728	0.000007
**C**	-1.274812	-0.628328	-0.000007
**C**	0.100435	1.523198	0.000020
**O**	-0.143665	-2.744122	0.000014
**N**	2.424821	-1.228594	0.000001
**N**	2.585910	0.994313	0.000001
**C**	1.149654	-0.752728	-0.000007
**C**	1.274812	0.628328	0.000007
**C**	-0.100435	-1.523198	-0.000023
**C**	3.264612	-0.148986	0.000003
**C**	-3.264612	0.148986	-0.000001
**H**	2.715131	-2.197265	-0.000001
**H**	-2.715131	2.197265	0.000000
**C**	4.695621	-0.334285	0.000003
**C**	6.608573	-1.304136	0.000010
**C**	6.854521	0.047911	-0.000005
**H**	7.325354	-2.108959	0.000015
**H**	7.770260	0.613326	-0.000012
**C**	-4.695621	0.334285	-0.000001
**C**	-6.608573	1.304136	0.000004
**C**	-6.854521	-0.047911	-0.000006
**H**	-7.325354	2.108959	0.000007
**H**	-7.770260	-0.613326	-0.000009
**N**	5.630238	0.663994	0.000002
**N**	5.263668	-1.535146	0.000002
**N**	-5.263668	1.535146	-0.000002
**N**	-5.630238	-0.663994	-0.000002
**C**	-5.407879	-2.108864	-0.000002
**H**	-4.846700	-2.401060	-0.884724
**H**	-4.846705	-2.401062	0.884722
**H**	-6.376416	-2.601580	-0.000006
**C**	5.407879	2.108864	0.000000
**H**	4.846700	2.401060	-0.884722
**H**	4.846704	2.401063	0.884724
**H**	6.376416	2.601580	-0.000003

Reduced

*Table S 58: **4**$_{MeImid}$ Reduced - Coordinates (B3LYP/TZVP, PCM (Water))*

Element	x	y	z
**O**	-0.202230	2.823609	-0.000008
**N**	2.406220	1.266244	-0.000002
**N**	2.565933	-0.968922	0.000004
**C**	1.103207	0.806272	-0.000001

**C**	1.230811	-0.611318	0.000002
**C**	-0.119948	1.457148	-0.000004
**O**	0.202230	-2.823609	0.000008
**N**	-2.406220	-1.266244	0.000002
**N**	-2.565933	0.968922	-0.000004
**C**	-1.103207	-0.806272	0.000001
**C**	-1.230811	0.611318	-0.000002
**C**	0.119948	-1.457148	0.000004
**C**	-3.231532	-0.169688	-0.000001
**C**	3.231532	0.169688	0.000001
**H**	-2.720457	-2.223924	0.000004
**H**	2.720457	2.223924	-0.000004
**C**	-4.665783	-0.350809	-0.000002
**C**	-6.586926	-1.310103	-0.000006
**C**	-6.825884	0.042076	-0.000002
**H**	-7.307763	-2.111465	-0.000009
**H**	-7.738309	0.612904	-0.000001
**C**	4.665783	0.350809	0.000002
**C**	6.586926	1.310103	0.000005
**C**	6.825884	-0.042076	0.000002
**H**	7.307763	2.111465	0.000007
**H**	7.738309	-0.612904	0.000002
**N**	-5.596888	0.651576	0.000000
**N**	-5.242017	-1.548358	-0.000005
**N**	5.242017	1.548358	0.000005
**N**	5.596888	-0.651576	0.000000
**C**	5.369120	-2.094769	-0.000003
**H**	4.805789	-2.385016	0.884029
**H**	4.805768	-2.385009	-0.884024
**H**	6.335774	-2.591634	-0.000016
**C**	-5.369120	2.094769	0.000003
**H**	-4.805769	2.385009	0.884025
**H**	-4.805789	2.385016	-0.884028
**H**	-6.335774	2.591634	0.000017
**H**	1.137039	-3.073410	0.000009
**H**	-1.137039	3.073410	-0.000009

Bis-4-pyridino-bis-imidazolo-1,4-benzoquinone Diiodide (**8$_{0Me}$**)

Oxidised

*Table S 59: **8$_{0Me}$** Oxidised - Coordinates (B3LYP/TZVP, PCM (Water))*

Element	x	y	z
**O**	0.007212	-2.748540	0.012129

**N**	2.484562	-1.095565	-0.000241
**N**	2.527295	1.126756	0.007330
**C**	1.187393	-0.688415	0.005293
**C**	1.239380	0.697521	0.008072
**C**	-0.018615	-1.528522	0.009049
**O**	-0.007212	2.748541	0.011953
**N**	-2.484561	1.095566	-0.000321
**N**	-2.527294	-1.126754	0.007392
**C**	-1.187393	0.688417	0.005245
**C**	-1.239380	-0.697520	0.008113
**C**	0.018615	1.528523	0.008952
**C**	-3.271535	-0.025663	0.000727
**C**	3.271535	0.025665	0.000729
**H**	-2.785476	2.060154	0.019101
**H**	2.785478	-2.060152	0.019248
**C**	-4.732499	-0.003283	-0.005620
**C**	-5.445246	-1.180809	0.239592
**C**	-6.852914	1.093591	-0.241796
**C**	-6.831813	-1.139368	0.227873
**H**	-4.923829	-2.106366	0.438053
**H**	-7.436915	1.986667	-0.434265
**H**	-7.401497	-2.042242	0.417732
**C**	4.732499	0.003283	-0.005617
**C**	5.445247	1.180813	0.239575
**C**	6.852912	-1.093598	-0.241771
**C**	6.831814	1.139371	0.227857
**H**	4.923830	2.106374	0.438019
**H**	7.436913	-1.986678	-0.434223
**H**	7.401499	2.042247	0.417700
**N**	-7.540701	-0.027926	-0.005383
**N**	7.540701	0.027923	-0.005378
**C**	-5.465344	1.159724	-0.255838
**H**	-4.988507	2.106000	-0.472793
**C**	5.465343	-1.159730	-0.255813
**H**	4.988503	-2.106008	-0.472752

Reduced

*Table S 60: **8**$_{0Me}$ Reduced - Coordinates (B3LYP/TZVP, PCM (Water))*

Element	x	y	z
**O**	0.040957	2.830166	-0.052811
**N**	-2.468619	1.132815	-0.016218
**N**	-2.506204	-1.101255	0.022108
**C**	-1.143533	0.743709	-0.012044

**C**	-1.195059	-0.678851	0.014246
**C**	0.039642	1.463779	-0.027468
**O**	-0.040957	-2.830165	0.052806
**N**	2.468619	-1.132815	0.016209
**N**	2.506204	1.101255	-0.022109
**C**	1.143533	-0.743708	0.012032
**C**	1.195059	0.678852	-0.014255
**C**	-0.039642	-1.463778	0.027459
**C**	3.239383	0.005446	-0.002781
**C**	-3.239383	-0.005445	0.002779
**H**	2.792895	-2.085401	0.050389
**H**	-2.792895	2.085402	-0.050394
**C**	4.701769	-0.011349	-0.002005
**C**	5.412697	1.192731	0.049165
**C**	6.831462	-1.121125	-0.046099
**C**	6.798677	1.157257	0.049285
**H**	4.886549	2.135834	0.089010
**H**	7.419191	-2.031725	-0.084560
**H**	7.363353	2.082287	0.089204
**C**	-4.701769	0.011349	0.002006
**C**	-5.412697	-1.192733	-0.049135
**C**	-6.831463	1.121125	0.046086
**C**	-6.798677	-1.157259	-0.049251
**H**	-4.886549	-2.135836	-0.088962
**H**	-7.419192	2.031726	0.084529
**H**	-7.363353	-2.082290	-0.089147
**N**	7.515248	0.026279	0.003419
**N**	-7.515248	-0.026280	-0.003405
**C**	5.444046	-1.194972	-0.051720
**H**	4.974924	-2.168338	-0.099276
**C**	-5.444047	1.194973	0.051699
**H**	-4.974925	2.168341	0.099230
**H**	-0.958101	-3.138662	0.059047
**H**	0.958101	3.138663	-0.059055

Bis-methyl-4-pyridinium-bis-methyl-imidazolo-1,4-benzoquinone Diiodide (**20_{4Me}**)

Oxidised

*Table S 61: **8_{4Me}** Oxidised - Coordinates (B3LYP/TZVP, PCM (Water))*

Element	x	y	z
**O**	0.095083	2.732954	0.276644
**N**	-2.487313	1.149824	0.146629
**N**	-2.526260	-1.076899	-0.098675

**C**	-1.189847	0.726712	0.087403
**C**	-1.244052	-0.653958	-0.069116
**C**	0.049839	1.519819	0.162644
**O**	-0.095084	-2.732953	-0.276638
**N**	2.487314	-1.149823	-0.146625
**N**	2.526260	1.076902	0.098676
**C**	1.189847	-0.726711	-0.087399
**C**	1.244051	0.653960	0.069121
**C**	-0.049839	-1.519818	-0.162640
**C**	3.264264	-0.021709	-0.018117
**C**	-3.264265	0.021711	0.018121
**C**	4.724685	0.022339	0.000873
**C**	5.522467	-0.982117	0.555855
**C**	5.364971	1.158728	-0.513602
**C**	6.893398	-0.843394	0.561098
**H**	5.103039	-1.862312	1.016159
**C**	6.733983	1.246651	-0.482492
**H**	4.791447	1.966804	-0.941458
**H**	7.543430	-1.594301	0.982829
**H**	7.271006	2.095824	-0.877405
**C**	-4.724685	-0.022338	-0.000872
**C**	-5.522467	0.982114	-0.555861
**C**	-5.364972	-1.158725	0.513607
**C**	-6.893398	0.843390	-0.561108
**H**	-5.103038	1.862307	-1.016170
**C**	-6.733983	-1.246650	0.482493
**H**	-4.791448	-1.966798	0.941469
**H**	-7.543430	1.594293	-0.982846
**H**	-7.271007	-2.095822	0.877408
**N**	7.482753	0.251527	0.044910
**N**	-7.482753	-0.251529	-0.044917
**C**	8.957764	0.381867	0.036132
**H**	9.230432	1.321422	0.510121
**H**	9.390234	-0.446592	0.586831
**H**	9.305238	0.364827	-0.994328
**C**	-8.957764	-0.381873	-0.036146
**H**	-9.230428	-1.321424	-0.510145
**H**	-9.305242	-0.364845	0.994314
**H**	-9.390234	0.446591	-0.586837
**C**	2.927372	-2.525427	-0.413652
**H**	3.044372	-3.069509	0.522301
**H**	2.174136	-3.017466	-1.018053
**H**	3.868371	-2.499799	-0.954649
**C**	-2.927370	2.525429	0.413657

**H**	-2.174135	3.017464	1.018063
**H**	-3.044364	3.069514	-0.522295
**H**	-3.868370	2.499802	0.954650

Reduced

*Table S 62: **8**$_{4Me}$ Reduced - Coordinates (B3LYP/TZVP, PCM (Water))*

Element	x	y	z
**O**	0.132647	-2.828514	-0.044888
**N**	-2.468758	-1.182379	-0.052289
**N**	-2.500950	1.069381	-0.066526
**C**	-1.142862	-0.774310	-0.061860
**C**	-1.196421	0.650617	-0.067507
**C**	0.060395	-1.465949	-0.061900
**O**	-0.132648	2.828539	-0.044135
**N**	2.468759	1.182405	-0.051956
**N**	2.500948	-1.069351	-0.066784
**C**	1.142863	0.774339	-0.061644
**C**	1.196422	-0.650586	-0.067667
**C**	-0.060396	1.465978	-0.061509
**C**	3.228087	0.032606	-0.044160
**C**	-3.228085	-0.032583	-0.044197
**C**	4.686490	-0.017182	0.014505
**C**	5.350458	-1.093672	-0.592226
**C**	5.463530	0.924611	0.697978
**C**	6.717548	-1.184534	-0.523376
**H**	4.794348	-1.852824	-1.120744
**C**	6.832714	0.785613	0.737895
**H**	5.023433	1.751730	1.231838
**H**	7.270780	-1.987514	-0.986472
**H**	7.465764	1.487828	1.258089
**C**	-4.686491	0.017179	0.014476
**C**	-5.463516	-0.924752	0.697773
**C**	-5.350472	1.093772	-0.592057
**C**	-6.832700	-0.785774	0.737726
**H**	-5.023405	-1.751967	1.231474
**C**	-6.717563	1.184604	-0.523187
**H**	-4.794372	1.853027	-1.120438
**H**	-7.465740	-1.488091	1.257795
**H**	-7.270807	1.987662	-0.986136
**N**	7.444966	-0.250340	0.131460
**N**	-7.444966	0.250284	0.131485
**C**	8.920808	-0.359910	0.142108
**H**	9.197353	-1.389447	0.352626

**H**	9.303059	-0.059485	-0.831427
**H**	9.318744	0.291025	0.913721
**C**	-8.920809	0.359846	0.142173
**H**	-9.197354	1.389327	0.352968
**H**	-9.303068	0.059678	-0.831437
**H**	-9.318736	-0.291296	0.913616
**C**	2.920155	2.566974	-0.202182
**H**	2.180813	3.112615	-0.779415
**H**	3.034394	3.046970	0.769796
**H**	3.865446	2.587172	-0.737848
**C**	-2.920155	-2.566907	-0.202893
**H**	-2.180818	-3.112388	-0.780284
**H**	-3.034382	-3.047172	0.768953
**H**	-3.865451	-2.586955	-0.738555
**H**	-1.066512	3.084575	-0.036231
**H**	1.066511	-3.084553	-0.037047

Bis-methyl-4-pyridinium-bis-dimethyl-imidazolo-1,4-benzoquinone Diiodide (**8**$_{\mathbf{6Me}}$)

Oxidised

*Table S 63: **8**$_{6Me}$ Oxidised - Coordinates (B3LYP/TZVP, PCM (Water))*

Element	x	y	z
**O**	0.001942	-2.727756	-0.226880
**N**	2.524281	-1.088256	-0.120401
**N**	2.522693	1.092278	0.119492
**C**	1.216324	-0.681014	-0.074763
**C**	1.215313	0.683004	0.073881
**C**	0.001100	-1.520653	-0.140975
**O**	-0.001942	2.727756	0.226878
**N**	-2.524281	1.088257	0.120399
**N**	-2.522693	-1.092278	-0.119493
**C**	-1.216324	0.681014	0.074760
**C**	-1.215313	-0.683003	-0.073883
**C**	-0.001100	1.520654	0.140972
**C**	-3.304223	-0.002627	0.000253
**C**	3.304223	0.002628	-0.000253
**C**	-4.775857	-0.002625	-0.000559
**C**	-5.478763	-0.653340	1.011512
**C**	-5.475535	0.652669	-1.010902
**C**	-6.857300	-0.630139	0.987664
**H**	-4.976074	-1.162180	1.820649
**C**	-6.854758	0.645238	-0.981099
**H**	-4.970833	1.158398	-1.820718

**H**	-7.456214	-1.114909	1.743341
**H**	-7.450436	1.133926	-1.736442
**C**	4.775857	0.002626	0.000560
**C**	5.478765	0.653340	-1.011511
**C**	5.475534	-0.652670	1.010903
**C**	6.857301	0.630139	-0.987661
**H**	4.976076	1.162181	-1.820647
**C**	6.854757	-0.645240	0.981101
**H**	4.970831	-1.158399	1.820718
**H**	7.456216	1.114909	-1.743337
**H**	7.450433	-1.133929	1.736444
**N**	-7.518615	0.013907	0.005974
**N**	7.518615	-0.013909	-0.005971
**C**	-9.002837	-0.012909	-0.008096
**H**	-9.324085	-0.930827	-0.496028
**H**	-9.363849	0.018596	1.015115
**H**	-9.364581	0.851086	-0.555533
**C**	9.002837	0.012906	0.008099
**H**	9.324086	0.930832	0.496016
**H**	9.364580	-0.851080	0.555549
**H**	9.363848	-0.018616	-1.015112
**C**	-2.985351	2.477245	0.311743
**H**	-2.409362	2.920064	1.117178
**H**	-2.826819	3.031865	-0.609564
**H**	-4.037700	2.462680	0.572213
**C**	2.985351	-2.477244	-0.311746
**H**	2.826817	-3.031865	0.609561
**H**	2.409363	-2.920063	-1.117182
**H**	4.037700	-2.462679	-0.572214
**C**	-2.981891	-2.481825	-0.310942
**H**	-2.408357	-2.922566	-1.119313
**H**	-2.818607	-3.037341	0.608975
**H**	-4.035351	-2.468734	-0.567098
**C**	2.981891	2.481825	0.310943
**H**	2.818605	3.037343	-0.608974
**H**	2.408358	2.922565	1.119315
**H**	4.035351	2.468735	0.567096

Reduced

*Table S 64: **8**$_{6Me}$ Reduced - Coordinates (B3LYP/TZVP, PCM (Water))*

Element	x	y	z
**O**	-0.026434	2.813082	-0.301378
**N**	-2.502295	1.091676	-0.121094

**N**	-2.501364	-1.100057	0.102265
**C**	-1.168184	0.699506	-0.078096
**C**	-1.167225	-0.707578	0.056621
**C**	-0.003118	1.461738	-0.117948
**O**	0.026457	-2.813085	0.301325
**N**	2.502294	-1.091679	0.121077
**N**	2.501364	1.100056	-0.102269
**C**	1.168184	-0.699507	0.078073
**C**	1.167226	0.707576	-0.056638
**C**	0.003119	-1.461740	0.117920
**C**	3.265450	0.004118	0.008422
**C**	-3.265449	-0.004120	-0.008427
**C**	4.738476	0.004393	0.002453
**C**	5.445859	0.664203	1.005483
**C**	5.435992	-0.660082	-1.003447
**C**	6.824054	0.638169	0.979628
**H**	4.945479	1.182826	1.809740
**C**	6.815033	-0.653181	-0.978464
**H**	4.927940	-1.174463	-1.805621
**H**	7.425537	1.128164	1.729889
**H**	7.408353	-1.148661	-1.731254
**C**	-4.738476	-0.004394	-0.002447
**C**	-5.445864	-0.664214	-1.005467
**C**	-5.435987	0.660093	1.003448
**C**	-6.824060	-0.638178	-0.979605
**H**	-4.945489	-1.182847	-1.809720
**C**	-6.815028	0.653194	0.978472
**H**	-4.927931	1.174483	1.805614
**H**	-7.425546	-1.128181	-1.729859
**H**	-7.408343	1.148682	1.731260
**N**	7.482629	-0.015173	0.002034
**N**	-7.482629	0.015176	-0.002016
**C**	8.966563	0.008456	-0.015063
**H**	9.289557	0.921045	-0.511748
**H**	9.329568	-0.014693	1.007701
**H**	9.325653	-0.861381	-0.555016
**C**	-8.966563	-0.008451	0.015087
**H**	-9.289556	-0.921035	0.511781
**H**	-9.325649	0.861391	0.555035
**H**	-9.329572	0.014690	-1.007675
**C**	2.981763	-2.469772	0.323331
**H**	2.413671	-2.916826	1.131563
**H**	2.840878	-3.039679	-0.591875
**H**	4.033094	-2.441959	0.588121

**C**	-2.981771	2.469767	-0.323344
**H**	-2.840955	3.039654	0.591886
**H**	-2.413635	2.916850	-1.131528
**H**	-4.033085	2.441945	-0.588203
**C**	2.982517	2.475310	-0.318961
**H**	2.376373	2.932696	-1.093365
**H**	2.902129	3.040993	0.606535
**H**	4.017166	2.440381	-0.642513
**C**	-2.982514	-2.475305	0.319004
**H**	-2.902152	-3.041016	-0.606478
**H**	-2.376354	-2.932672	1.093407
**H**	-4.017155	-2.440366	0.642579
**H**	-0.143615	-3.281430	-0.528493
**H**	0.143477	3.281444	0.528464

References

(1) Sheldrick, G. M. SHELXT - Integrated Space-Group and Crystal-Structure Determination. *Acta Crystallogr. Sect. A Found. Crystallogr.* **2015**, *71* (1), 3–8. https://doi.org/10.1107/S2053273314026370.

(2) Sheldrick, G. M. Crystal Structure Refinement with SHELXL. *Acta Crystallogr. Sect. C Struct. Chem.* **2015**, *71* (1), 3–8. https://doi.org/10.1107/S2053229614024218.

(3) Fulmer, G. R.; Miller, A. J. M.; Sherden, N. H.; Gottlieb, H. E.; Nudelman, A.; Stoltz, B. M.; Bercaw, J. E.; Goldberg, K. I. NMR Chemical Shifts of Trace Impurities: Common Laboratory Solvents, Organics, and Gases in Deuterated Solvents Relevant to the Organometallic Chemist. *Organometallics* **2010**, *29* (9), 2176–2179. https://doi.org/10.1021/om100106e.

(4) Elgrishi, N.; Rountree, K. J.; McCarthy, B. D.; Rountree, E. S.; Eisenhart, T. T.; Dempsey, J. L. A Practical Beginner's Guide to Cyclic Voltammetry. *J. Chem. Educ.* **2018**, *95* (2), 197–206. https://doi.org/10.1021/acs.jchemed.7b00361.

(5) Zhao, E. W.; Liu, T.; Jónsson, E.; Lee, J.; Temprano, I.; Jethwa, R. B.; Wang, A.; Smith, H.; Carretero-González, J.; Song, Q.; Grey, C. P. In Situ NMR Metrology Reveals Reaction Mechanisms in Redox Flow Batteries. *Nature* **2020**, *579* (7798), 224–228. https://doi.org/10.1038/s41586-020-2081-7.

(6) Lin, K.; Chen, Q.; Gerhardt, M. R.; Tong, L.; Kim, S. B.; Eisenach, L.; Valle, A. W.; Hardee, D.; Gordon, R. G.; Aziz, M. J.; Marshak, M. P. Alkaline Quinone Flow Battery. *Science* **2015**, *349* (6255), 1529–1532. https://doi.org/10.1126/science.aab3033.

(7) Zhao, E. W.; Jónsson, E.; Jethwa, R. B.; Hey, D.; Lyu, D.; Brookfield, A.; Klusener, P. A. A.; Collison, D.; Grey, C. P. Coupled in Situ NMR and EPR Studies Reveal the Electron Transfer Rate and Electrolyte Decomposition in Redox Flow Batteries. *J. Am. Chem. Soc.* **2021**, *143* (4), 1885–1895. https://doi.org/10.1021/jacs.0c10650.

(8) Stoll, S.; Schweiger, A. EasySpin, a Comprehensive Software Package for Spectral Simulation and Analysis in EPR. *J. Magn. Reson.* **2006**, *178* (1), 42–55. https://doi.org/10.1016/J.JMR.2005.08.013.

(9) Frisch, M. J.; Trucks, G. W.; Schlegel, H. B.; Scuseria, G. E.; Robb, M. A.; Cheeseman, J. R.; Scalmani, G.; Barone, V.; Petersson, G. A.; Nakatsuji, H.; Li, X.; Caricato, M.;

Marenich, A. V.; Bloino, J.; Janesko, B. G.; Gomperts, R.; Mennucci, B.; Hratchian, H. P.; Ortiz, J. V.; Izmaylov, A. F.; Sonnenberg, J. L.; Williams-Young, D.; Ding, F.; Lipparini, F.; Egidi, F.; Goings, J.; Peng, B.; Petrone, A.; Henderson, T.; Ranasinghe, D.; Zakrzewski, V. G.; Gao, J.; Rega, N.; Zheng, G.; Liang, W.; Hada, M.; Ehara, M.; Toyota, K.; Fukuda, R.; Hasegawa, J.; Ishida, M.; Nakajima, T.; Honda, Y.; Kitao, O.; Nakai, H.; Vreven, T.; Throssell, K.; Montgomery, J. A., J.; Peralta, J. E.; Ogliaro, F.; Bearpark, M. J.; Heyd, J. J.; Brothers, E. N.; Kudin, K. N.; Staroverov, V. N.; Keith, T. A.; Kobayashi, R.; Normand, J.; Raghavachari, K.; Rendell, A. P.; Burant, J. C.; Iyengar, S. S.; Tomasi, J.; Cossi, M.; Millam, J. M.; Klene, M.; Adamo, C.; Cammi, R.; Ochterski, J. W.; Martin, R. L.; Morokuma, K.; Farkas, O.; Foresman, J. B.; Fox, D. J. Gaussian. Gaussian, Inc., Wallingford CT 2016. http://gaussian.com/citation/.

(10) Perdew, J. P.; Burke, K.; Ernzerhof, M. Generalized Gradient Approximation Made Simple. *Phys. Rev. Lett.* **1996**, *77* (18), 3865–3868. https://doi.org/10.1103/PhysRevLett.77.3865.

(11) Perdew, J. P.; Burke, K.; Ernzerhof, M. Erratum: Generalized Gradient Approximation Made Simple (Physical Review Letters (1996) 77 (3865)). *Phys. Rev. Lett.* **1997**, *78* (8), 1396. https://doi.org/10.1103/PhysRevLett.78.1396.

(12) Schäfer, A.; Huber, C.; Ahlrichs, R. Fully Optimized Contracted Gaussian Basis Sets of Triple Zeta Valence Quality for Atoms Li to Kr. *J. Chem. Phys.* **1994**, *100* (8), 5829–5835. https://doi.org/10.1063/1.467146.

(13) Schäfer, A.; Horn, H.; Ahlrichs, R. Fully Optimized Contracted Gaussian Basis Sets for Atoms Li to Kr. *J. Chem. Phys.* **1992**, *97* (4), 2571–2577. https://doi.org/10.1063/1.463096.

(14) Grimme, S.; Antony, J.; Ehrlich, S.; Krieg, H. A Consistent and Accurate Ab Initio Parametrization of Density Functional Dispersion Correction (DFT-D) for the 94 Elements H-Pu. *J. Chem. Phys.* **2010**, *132* (15), 154104. https://doi.org/10.1063/1.3382344.

(15) Becke, A. D. Density-Functional Thermochemistry. III. The Role of Exact Exchange. *J. Chem. Phys.* **1993**, *98* (7), 5648–5652. https://doi.org/10.1063/1.464913.

(16) Scalmani, G.; Frisch, M. J. Continuous Surface Charge Polarizable Continuum Models of Solvation. I. General Formalism. *J. Chem. Phys.* **2010**, *132* (11), 114110.

https://doi.org/10.1063/1.3359469.

(17) Marenich, A. V.; Cramer, C. J.; Truhlar, D. G. Universal Solvation Model Based on Solute Electron Density and on a Continuum Model of the Solvent Defined by the Bulk Dielectric Constant and Atomic Surface Tensions. *J. Phys. Chem. B* **2009**, *113* (18), 6378–6396. https://doi.org/10.1021/jp810292n.

(18) Atkins, P.; de Paula, J. *Atkins' Physical Chemistry*, Seventh.; Oxford University Press: New York, 2002.

(19) Chong, D. P. *Recent Advances in Density Functional Methods, p. 2*; Chong, D. P., Ed.; Singapore World Scientific: Singapore, 1997.

(20) Yanai, T.; Tew, D. P.; Handy, N. C. A New Hybrid Exchange-Correlation Functional Using the Coulomb-Attenuating Method (CAM-B3LYP). *Chem. Phys. Lett.* **2004**, *393* (1–3), 51–57. https://doi.org/10.1016/j.cplett.2004.06.011.

(21) Bauernschmitt, R.; Ahlrichs, R. Treatment of Electronic Excitations within the Adiabatic Approximation of Time Dependent Density Functional Theory. *Chem. Phys. Lett.* **1996**, *256* (4–5), 454–464. https://doi.org/10.1016/0009-2614(96)00440-X.

(22) Casida, M. E.; Jamorski, C.; Casida, K. C.; Salahub, D. R. Molecular Excitation Energies to High-Lying Bound States from Time-Dependent Density-Functional Response Theory: Characterization and Correction of the Time-Dependent Local Density Approximation Ionization Threshold. *J. Chem. Phys.* **1998**, *108* (11), 4439–4449. https://doi.org/10.1063/1.475855.

(23) Stratmann, R. E.; Scuseria, G. E.; Frisch, M. J. An Efficient Implementation of Time-Dependent Density-Functional Theory for the Calculation of Excitation Energies of Large Molecules. *J. Chem. Phys.* **1998**, *109* (19), 8218–8224. https://doi.org/10.1063/1.477483.

(24) Van Caillie, C.; Amos, R. D. Geometric Derivatives of Excitation Energies Using SCF and DFT. *Chem. Phys. Lett.* **1999**, *308* (3–4), 249–255. https://doi.org/10.1016/S0009-2614(99)00646-6.

(25) Van Caillie, C.; Amos, R. D. Geometric Derivatives of Density Functional Theory Excitation Energies Using Gradient-Corrected Functionals. *Chem. Phys. Lett.* **2000**, *317* (1–2), 159–164. https://doi.org/10.1016/S0009-2614(99)01346-9.

(26) Furche, F.; Ahlrichs, R. Adiabatic Time-Dependent Density Functional Methods for Excited State Properties. *J. Chem. Phys.* **2002**, *117* (16), 7433–7447. https://doi.org/10.1063/1.1508368.

(27) Scalmani, G.; Frisch, M. J.; Mennucci, B.; Tomasi, J.; Cammi, R.; Barone, V. Geometries and Properties of Excited States in the Gas Phase and in Solution: Theory and Application of a Time-Dependent Density Functional Theory Polarizable Continuum Model. *J. Chem. Phys.* **2006**, *124* (9), 094107. https://doi.org/10.1063/1.2173258.

(28) Sato, M.; Takeda, T.; Hoshino, N.; Akutagawa, T. Electronic and Crystal Structures of 1,2,3-Triazole-Fused p-Benzoquinone Derivatives. *CrystEngComm* **2017**, *19* (6), 910–917. https://doi.org/10.1039/C6CE02167K.

(29) Cao, J.; Tian, J.; Xu, J.; Wang, Y. Organic Flow Batteries: Recent Progress and Perspectives. *Energy and Fuels* **2020**, *34* (11), 13384–13411. https://doi.org/10.1021/acs.energyfuels.0c02855.

(30) Bunzen, H.; Lamp, A.; Grzywa, M.; Barkschat, C.; Volkmer, D. Bistriazole-p-Benzoquinone and Its Alkali Salts: Electrochemical Behaviour in Aqueous Alkaline Solutions. *Dalt. Trans.* **2017**, *46* (37), 12537–12543. https://doi.org/10.1039/c7dt02803b.

(31) Frisch, M. J.; Trucks, G. W.; Schlegel, H. B.; Scuseria, G. E.; Robb, M. A.; Cheeseman, J. R.; Scalmani, G.; Barone, V.; Petersson, G. A.; Nakatsuji, H.; Li, X.; Caricato, M.; Marenich, A. V.; Bloino, J.; Janesko, B. G.; Gomperts, R.; Mennucci, B.; Hratchian, H. P.; Ortiz, J. V.; Izmaylov, A. F.; Sonnenberg, J. L.; Williams-Young, D.; Ding, F.; Lipparini, F.; Egidi, F.; Goings, J.; Peng, B.; Petrone, A.; Henderson, T.; Ranasinghe, D.; Zakrzewski, V. G.; Gao, J.; Rega, N.; Zheng, G.; Liang, W.; Hada, M.; Ehara, M.; Toyota, K.; Fukuda, R.; Hasegawa, J.; Ishida, M.; Nakajima, T.; Honda, Y.; Kitao, O.; Nakai, H.; Vreven, T.; Throssell, K.; Montgomery, J. A., J.; Peralta, J. E.; Ogliaro, F.; Bearpark, M. J.; Heyd, J. J.; Brothers, E. N.; Kudin, K. N.; Staroverov, V. N.; Keith, T. A.; Kobayashi, R.; Normand, J.; Raghavachari, K.; Rendell, A. P.; Burant, J. C.; Iyengar, S. S.; Tomasi, J.; Cossi, M.; Millam, J. M.; Klene, M.; Adamo, C.; Cammi, R.; Ochterski, J. W.; Martin, R. L.; Morokuma, K.; Farkas, O.; Foresman, J. B.; Fox, D. J. *Creating UV/Visible Plots from the Results of Excited States Calculations*. Gaussian Inc Pittsburgh PA. https://gaussian.com/uvvisplot/ (accessed 2022-01-03).

(32) Manivannan, R.; Ciattini, S.; Chelazzi, L.; Elango, K. P. Benzoquinone-Imidazole Hybrids as Selective Colorimetric Sensors for Cyanide in Aqueous, Solid and Gas Phases. *RSC Adv.* **2015**, *5* (106), 87341–87351. https://doi.org/10.1039/c5ra13597d.

(33) Wang, Z.; Peng, Q.; Huang, X.; Ma, Q.; Shen, Q.; Shao, J. Linear Acenaphthene-Imide-Fused Pyrazinacene-Quinone: Synthesis and Photophysical Properties. *Mater. Technol.* **2020**, 1–8. https://doi.org/10.1080/10667857.2020.1810925.

(34) Winkelmann, E. 2,3,5,6-Tetra-Amino-1,4-Benzochinon (TABC). *Tetrahedron* **1969**, *25* (12), 2427–2454. https://doi.org/10.1016/S0040-4020(01)82792-7.

(35) Liu, Y.; Phan, H.; Herng, T. S.; Gopalakrishna, T. Y.; Ding, J.; Wu, J. Toward Benzobis(Thiadiazole)-Based Diradicaloids. *Chem. - An Asian J.* **2017**, *12* (17), 2177–2182. https://doi.org/10.1002/asia.201700732.

(36) Neidlein, R.; Tran-Viet, D.; Gieren, A.; Kokkinidis, M.; Wilckens, R.; Geserich, H. -P; Ruppel, W. Synthesen, Konstitutionsaufklärung, Röntgenstrukturanalyse Und Elektrische Eigenschaften von Di- Und Polychalkogendiimiden, 1. Mitteilung. *Chem. Ber.* **1982**, *115* (8), 2898–2904. https://doi.org/10.1002/cber.19821150820.

(37) Manivannan, R.; Elango, K. P. Spectral and Electrochemical Studies on Anion Recognition by Ferrocene Based Imidazoles Possessing Different Electron Acceptor Moieties. *J. Organomet. Chem.* **2015**, *799–800,* 99–107. https://doi.org/10.1016/j.jorganchem.2015.09.029.

(38) Ansari, M. A.; Beyer, K.; Schwederski, B.; Kaim, W.; Lahiri, G. K. Diruthenium Complexes of *p* -Benzoquinone-Imidazole Hybrid Ligands: Innocent or Noninnocent Behavior of the Quinone Moiety. *Chem. - An Asian J.* **2018**, *13* (19), 2947–2955. https://doi.org/10.1002/asia.201801101.

(39) Karayannidis, G. P.; Sideridou-Karayannidou, I. Use of Aromatic Dialdehydes in the Preparation of Poly(P-benzoquinono)Diimidazoles. *J. Polym. Sci. Part A Polym. Chem.* **1987**, *25* (9), 2335–2341. https://doi.org/10.1002/pola.1987.080250902.

(40) Reznichenko, O.; Quillévéré, A.; Martins, R. P.; Loaëc, N.; Kang, H.; Lista, M. J.; Beauvineau, C.; González-García, J.; Guillot, R.; Voisset, C.; Daskalogianni, C.; Fåhraeus, R.; Teulade-Fichou, M. P.; Blondel, M.; Granzhan, A. Novel Cationic Bis(Acylhydrazones) as Modulators of Epstein–Barr Virus Immune Evasion Acting

through Disruption of Interaction between Nucleolin and G-Quadruplexes of EBNA1 MRNA. *Eur. J. Med. Chem.* **2019**, *178*, 13–29. https://doi.org/10.1016/J.EJMECH.2019.05.042.
